# Synthesis of Carborane–Thiazole Conjugates as Tyrosinase and 11β-Hydroxysteroid Dehydrogenase Inhibitors: Antiproliferative Activity and Molecular Docking Studies

**DOI:** 10.3390/molecules29194716

**Published:** 2024-10-05

**Authors:** Beata Donarska, Joanna Cytarska, Dominika Kołodziej-Sobczak, Renata Studzińska, Daria Kupczyk, Angelika Baranowska-Łączkowska, Karol Jaroch, Paulina Szeliska, Barbara Bojko, Daria Różycka, Agnieszka B. Olejniczak, Wojciech Płaziński, Krzysztof Z. Łączkowski

**Affiliations:** 1 Department of Chemical Technology and Pharmaceuticals, Faculty of Pharmacy, Collegium Medicum, Nicolaus Copernicus University, Jurasza 2, 85-089 Bydgoszcz, Poland; beata.donarska@cm.umk.pl (B.D.); cytar@cm.umk.pl (J.C.); d.kolodziejsobczak@gmail.com (D.K.-S.); 2 Department of Organic Chemistry, Faculty of Pharmacy, Collegium Medicum, Nicolaus Copernicus University, Jurasza 2, 85-089 Bydgoszcz, Poland; rstud@cm.umk.pl; 3 Department of Medical Biology and Biochemistry, Faculty of Medicine, Collegium Medicum, Nicolaus Copernicus University, Karłowicza 24, 85-092 Bydgoszcz, Poland; dariak@cm.umk.pl; 4 Faculty of Physics, Kazimierz Wielki University, Powstańców Wielkopolskich 2, 85-090 Bydgoszcz, Poland; 5 Department of Pharmacodynamics and Molecular Pharmacology, Faculty of Pharmacy, Collegium Medicum, Nicolaus Copernicus University, Jurasza 2, 85-089 Bydgoszcz, Poland; karol.jaroch@cm.umk.pl (K.J.); p.modrakowska@cm.umk.pl (P.S.); bbojko@cm.umk.pl (B.B.); 6 Screening Laboratory, Institute of Medical Biology, Polish Academy of Sciences, Lodowa 106, 93-232 Lodz, Poland; drozycka@cbm.pan.pl (D.R.); aolejniczak@cbm.pan.pl (A.B.O.); 7 Jerzy Haber Institute of Catalysis and Surface Chemistry, Polish Academy of Sciences, Niezapominajek 8, 30-239 Cracow, Poland; wojtek_plazinski@tlen.pl; 8 Department of Biopharmacy, Medical University of Lublin, Chodzki 4a, 20-093 Lublin, Poland

**Keywords:** carborane, thiazole, tyrosinase, antiproliferative activity, 11β-hydroxysteroid dehydrogenase, molecular docking

## Abstract

The presented study depicts the synthesis of 11 carborane–thiazole conjugates with anticancer activity, as well as an evaluation of their biological activity as inhibitors of two enzymes: tyrosinase and 11β-hydroxysteroid dehydrogenase type 1 (11β-HSD1). The overexpression of tyrosinase results in the intracellular accumulation of melanin and can be observed in melanoma. The overexpression of 11β-HSD1 results in an elevation of glucocorticoid levels and has been associated with the aggravation of metabolic disorders such as type II diabetes mellitus and obesity. Recently, as the comorbidity of melanomas and metabolic disorders is being recognized as an important issue, the search for new therapeutic options has intensified. This study demonstrates that carborane–thiazole derivatives inhibit both enzymes, exerting beneficial effects. The antiproliferative action of all newly synthesized compounds was evaluated using three cancer cell lines, namely A172 (human brain glioblastoma), B16F10 (murine melanoma) and MDA-MB-231 (human breast adenocarcinoma), as well as a healthy control cell line of HUVEC (human umbilical vein endothelial cells). The results show that 9 out of 11 newly synthesized compounds demonstrated similar antiproliferative action against the B16F10 cell line to the reference drug, and three of these compounds surpassed it. To the best of our knowledge, this study is the first to demonstrate dual inhibitory action of carborane–thiazole derivatives against both tyrosinase and 11β-HSD1. Therefore, it represents the first step towards the simultaneous treatment of melanoma and comorbid diseases such as type II diabetes mellitus.

## 1. Introduction

Cancer is one of the main causes of morbidity and mortality worldwide and was responsible for approximately 9.7 million deaths in 2022 [1]. According to estimates by Global Cancer Data (GLOBOCAN), in 2022, there were about 20 million new cancer cases, and by 2050, a total of 35 million new cases will be diagnosed per year [2]. Currently, the most frequently diagnosed cancer is breast cancer in women, which accounts for 11.7% of new cases, followed by lung (11.4%), colorectal (10.0%), prostate (7.3%), and gastric (5.6%) cancer [2]. Compared to the abovementioned cancers, melanoma—an aggressive type of skin cancer—is less often diagnosed (estimated as 5% of all new cases in 2024 [3]), but due to its rapid growth and potential to spread to any organ, it is a serious health threat. The increased activity of tyrosinase in this type of cancer leads to the accumulation of melanin in the cells, which protects it from radiotherapy due to the high capacity of melanin to absorb radiation [4]. The antioxidant properties of melanin may reduce the effectiveness of chemotherapy, and the intermediates formed in the melanogenesis process, such as 3,4-dihydroxy-L-phenylalanine (L-DOPA) and quinones, may also have an immunosuppressive effect, which makes the applied immunotherapy ineffective. A possible solution to this problem is the use of tyrosinase inhibitors, which inhibit the conversion of L-tyrosine to L-DOPA and then to L-DOPA-quinone, which is one of the precursors of melanin [5].

Diabetes is now one of the major diseases worldwide, and the high prevalence of the main risk factors for developing diabetes, namely obesity, a poor diet, and physical inactivity, causes the further growth of the number of diagnosed cases. It is estimated that 463 million people had diabetes in 2019, and this number could rise to 578 million in 2030 and up to 700 million in 2045 [6]. Type II diabetes mellitus (T2DM), which accounts for about 90% of cases, is associated with the progressive impairment of insulin secretion by pancreatic β-cells and the development of peripheral tissue resistance to insulin (insulin resistance) and consequently elevated blood glucose levels [7]. Chronic hyperglycaemia and the associated dysfunction of protein, lipid, and carbohydrate metabolism lead to numerous microvascular and macrovascular disorders, such as neuropathies, retinopathies, nephropathies and atherosclerosis, which worsen patients’ quality of life and also increase the risk of other serious diseases [8].

11β-Hydroxysteroid dehydrogenases (11β-HSD) are prereceptor enzymes that regulate the intracellular ability of active glucocorticoids (GC) to bind and activate the glucocorticoid receptor (GR). There are two types (isoforms): 11β-HSD1 and 11β-HSD2. They are products of two different genes, are present in different tissues, and have different physiological roles. 11β-HSD2, the first to be discovered, converts active glucocorticoids, such as cortisol (in humans) or corticosterone (in mice/rats), to inactive forms, such as cortisone (in humans) or 11-dihydrocorticosterone (DHC in rodents). The second enzyme isoform, 11β-HSD1, occurs in key metabolic tissues such as the liver, adipose tissue, and muscles, and works bidirectionally—it is capable of acting as both a reductase (activating GC) and dehydrogenase (deactivating GC) [9]. However, in intact cells such as hepatocytes and adipocytes, 11β-HSD1 has a higher affinity for cortisone than for cortisol. Excess glucocorticoids lead to abdominal obesity, insulin resistance, dyslipidemia, and hypertension, which are the most commonly recognized risk factors for metabolic syndrome [10]. Lowering the level of glucocorticoids may be a potential therapy for insulin resistance and other disorders in patients with T2DM. The inhibition of 11β-HSD1 activity causes a decrease in glucocorticoid levels. In consequence, it may result in a decrease in body fat mass, a decrease in glucose levels in the blood of patients with T2DM, and a reduction in the total cholesterol level [11].

An increasing number of scientific studies indicate that cancer and diabetes mellitus are commonly coexisting illnesses. Postmenopausal breast cancer, endometrial cancer, and colorectal cancer closely correlate with diabetes [12]. Recent studies also suggest that type II diabetes mellitus is associated with increased cutaneous melanoma aggressiveness at diagnosis [13]. Also, evidence suggests that cancer and diabetes patients have a higher mortality rate, so a multi-pronged approach is important in treating patients.

Previous studies on the search for tyrosinase and selective 11β-HSD1 inhibitors, including our group’s research, were directed towards several groups of compounds, i.e., flavones, coumarins, chalcones, thiosemicarbazones, thiazole, kojic acid derivatives, cyrene^TM^, 2-(cyclopentylamino)thiazol-4(5H)-one, 2-amino-1,3-thiazol-4(5*H*)-ones, and thiazolo[3,2-a]pyrimidine-5-one derivatives [14,15,16,17,18,19,20]. However, to the best of our knowledge, carborane derivatives have never been tested as tyrosinase and 11β-HSD1 inhibitors.

Carboranes (dicarba-*closo*-dodecaboranes, C_2_H_12_B_10_) belong to the class of boron clusters in which BH vertices are substituted with neutral CH groups [21]. The interest in the use of carboranes in different areas of medicinal chemistry has grown over the years due to their properties including their abiotic origin, unique interaction properties with biomolecules, hydrophobicity or amphiphilicity, nearly spherical geometry, established chemistry, chemical stability and biological stability in vivo, resistance to catabolism, and low toxicity [22,23]. So far, many compounds bearing carborane clusters have revealed promising activity in several fields, such as in boron neutron capture therapy (BNCT) and the synthesis of nucleosides [24], amino acids [25], and potent antiviral [26], antimicrobial [27,28], and antiparasitic agents [29]. Biological enzymes have also been targeted using carborane derivatives [22,23]. Such conjugates show promising activity against cancer-associated carbonic anhydrase IX (CA IX) [30], hypoxia-inducible factor-1 (HIF-1) [31,32], nicotinamide phosphoribosyltransferase (NAMPT) [33], cyclooxygenase (COX) [34,35], lipoxygenase (LO) [36,37], and indoleamine-2,3-dioxygenase-1 (IDO1) [38].

Considering the abovementioned findings, we decided to design and synthesize hybrid structures incorporating both the carborane moiety and thiazole ring, the latter being a very good scaffold for obtaining antityrosinase activity, as confirmed by numerous studies by our group [39,40,41]. Additionally, the use of electron-donating, electron-withdrawing, and bulky character substituents enabled us to modify the hydrophilic and hydrophobic properties of the designed molecules. Next, the antiproliferative activity of these compounds against three human cancer cells lines, namely brain glioblastoma A172, murine melanoma B16F10, and human breast adenocarcinoma MDA-MB-231, as well as healthy human umbilical vein endothelial cells (HUVEC), was evaluated. To recognize the possible mechanism of action of the products, the tyrosinase inhibitory potential with an inhibitory mechanism was assessed, followed by molecular docking studies. Finally, we tested the inhibitory potential of our compounds against 11β-HSD1 and 11β-HSD2.

## 2. Results and Discussion

### 2.1. Chemistry

**Scheme 1:**
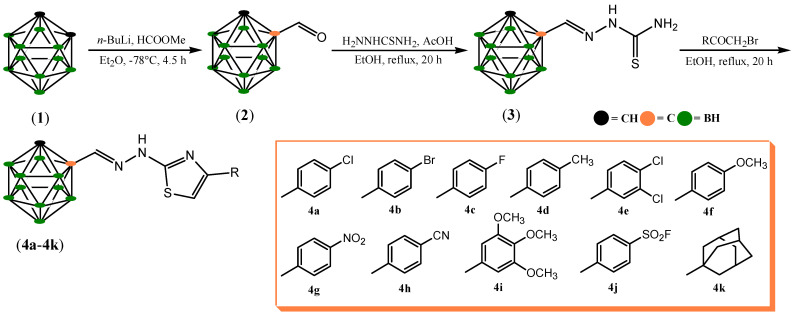
Synthesis of the carborane–thiazoles **4a**–**4k**.

The synthesis of the title compounds was accomplished following the well-known Hantzsch cyclization reaction. In the first step, C-formyl-*o*-carborane (**2**) was obtained by means of the formylation reaction of *o*-carborane (**1**) through treatment with *n*-BuLi at −78 °C and the subsequent addition of methyl formate. Hydrazonecarbothioamide **3** was synthesized with 89% yield through condensation of C-formyl-*o*-carborane (**2**) with thiosemicarbazide in absolute ethyl alcohol containing a catalytic amount of glacial acetic acid. In the next step, cyclization of hydrazonecarbothioamide **3** with a variety of substituted bromoacetophenones in ethanolic solution and under reflux produced 2-(2-(1,2-dicarba-*closo*-dodecaboranylmethylene)hydrazinyl)thiazole **4a**–**4k** with a high yield (60–99%). The reaction pathway is summarized in Scheme 1. All compounds were fully characterized spectroscopically using ^1^H, ^13^C, and ^11^B NMR and ESI-HRMS analysis. The ^1^H NMR spectrum of hydrazonecarbothioamide (**3**) showed three characteristic peaks derived from NH_2_ and NH groups at 8.08, 8.49, and 11.58 ppm, respectively. Carborane–thiazoles showed characteristic singlets derived from the CH group of the *o*-carborane moiety at 5.35–5.45 ppm and from the NH proton at about 12.17–12.60 ppm, whereas in the ^13^C NMR spectrum, we observe characteristic peaks at about 101–109 ppm and at about 170 ppm derived from C=N group.

### 2.2. Antiproliferative Activity

The antiproliferative activity of all newly synthesized carborane–thiazole derivates was evaluated on three cancer cell lines, namely A172 (human brain glioblastoma), B16F10 (murine melanoma) and MDA-MB-231 (human breast adenocarcinoma), as well as a healthy control cell line, HUVEC (human umbilical vein endothelial cells). As presented in Table 1, particularly good results were observed for the carborane–thiazole derivatives numbered **4a**–**4e**, **4g**, and **4j**. For all of these compounds, the selectivity index (SI) based on the ratios of recorded IC_50_ values was favorable. That is, the concentrations of inhibitors required to prevent proliferation of cancer cell lines were notably lower than the concentrations required to prevent the growth of healthy cell line (above 1.0). The following was also observed, albeit not in all cases for compounds **4f**, **4h**, and **4i**. In these cases, for compound **4f**, favorable SI values were observed only for the B16F10 and MDA-MB-231 cell lines, while for compounds **4h** and **4i**, they were only observed for the B16F10 cell line. On the contrary, unfavorable results were observed for compound **4k**, which was not effective against any of the cancer cell lines.

Considering the effects of investigated compounds on particular cell lines, the most favorable SI values were observed for the murine melanoma cell line. Therefore, the evaluated compounds are most suitable for the treatment of this cancer type. Moreover, the activity of all newly synthesized compounds (except for **4d** and **4k**) is comparable to the activity of the reference inhibitor of chlorambucil. Especially favorable were some of the recorded IC_50_ values, which surpassed the results observed for the reference inhibitor. Such was the case with compounds **4a** (5.03 ± 0.69 µM), **4b** (5.37 ± 0.11 µM), and **4g** (5.11 µM). Finally, the results of this study showed that the activity of the newly synthesized compounds was similar against all of the evaluated cancer cell lines, with all recorded IC_50_ values in similar ranges: for A172, from 5.77 ± 0.96 to 7.88 ± 0.98 µM; for B16F10, from 5.03 ± 0.69 to 5.88 µM; and for MDA-MB-231, from 6.02 ± 0.08 to 7.32 ± 1.26 µM. This is an especially beneficial trait, as it suggests universal applicability of the evaluated compounds.

The SAR analysis showed that the differences in activity between individual substituents were small, at around 2 µM. However, it can be easily seen that (-3,4,5-tri-OCH_3_) and adamantyl substituents drastically reduce the antiproliferative activity, most likely due to their bulky structure and high lipophilicity, especially for the compound **4k** containing an adamantyl substituent.

### 2.3. Mushroom Tyrosinase Inhibitory Effect and Kinetic Analysis of Compounds

The studies of inhibition activity against the mushroom tyrosinase and enzyme inhibition kinetics were performed for all synthesized compounds. The recorded IC_50_ values (shown in Table 2) ranged from 11.27 ± 5.90 µM for compound **4h** to 360.51 ± 118.59 µM for compound **4j**. After compound **4h**, the second most effective compound was **4f** with an IC_50_ value of 49.36 ± 8.01 µM; however, it appears to be a tyrosinase activator.

**Figure 1:**
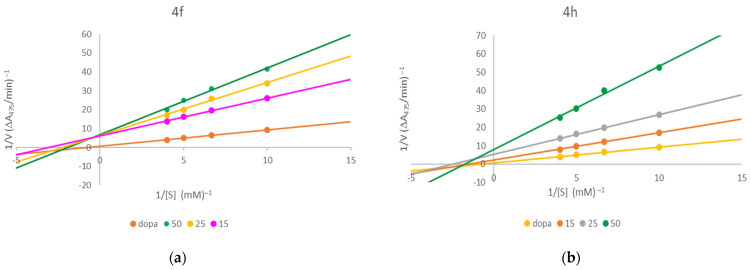
Lineweaver–Burk plots for tyrosinase inhibition for compounds **4f** (**a**) and **4h** (**b**).

Based on the Lineweaver–Burk double reciprocal plots presented in Figure 1, the inhibition mode for these two compounds was established as mixed type inhibition. Furthermore, out of all of the investigated compounds, **4h** and **4f** were the only ones determined to be more active against mushroom tyrosinase than the reference inhibitor of kojic acid. However, it should be stressed that the gold standard of mushroom tyrosinase inhibition is displayed by ascorbic acid rather than kojic acid. Remarkably, all of the synthesized compounds surpassed this widely established reference as enzyme inhibitors.

Summarizing the structure–activity relationship (SAR) findings, it can be seen that the type of halogen substituent, i.e., electron-withdrawing substituents (-Cl, -Br, -F), did not cause significant differences in the tyrosinase inhibition capacity of compounds **4a**–**4c** (IC_50_ = 130–144 mM). Also, the addition of a second chlorine atom did not lead to increased activity (compound **4e** (IC_50_ = 142 mM)). However, compound **4h**, containing an electron-withdrawing group –CN, showed the highest inhibitory activity (IC_50_ = 11 mM). This can be explained by the linear shape of this group, the strong electronegativity of the nitrogen atom, and the most favorable binding mode with the Cu ions present in the protein structure. The molecular docking confirms that this group has the lowest binding energy with tyrosinase (−7.9 kcal/mol). Compounds with electron-donating (-CH_3_) and bulky (adamantyl) groups showed similar activities (**4d** (IC_50_ = 121 mM) and **4k** (IC_50_ = 134 mM), respectively). Superior activity is shown by compound **4f** (IC_50_ = 49 mM) containing an electron-donating (-OCH_3_) group. At the same time, compounds containing the (-OCH_3_) or (-CN) ligands display the highest values of binding energy (−7.3–−7.9 kcal/mol).

### 2.4. Inhibition of 11β-HSD1

The seven derivatives were tested for their inhibition of 11β-hydroxysteroid dehydrogenase type 1. The inhibition of the dehydrogenase activity was determined as a percentage of the inhibition at the concentration of potential inhibitor of 10 μM. All tested derivatives showed inhibitory activity in the range of 55.54 to 73.51% at a concentration of 10 μM (see Table 3). The IC_50_ for all analyzed compounds was lower than 10 µM (in the range 3.90–9.35 µM). The known 11β-HSD1 inhibitor, carbenoxolone, is characterized by a higher percent of inhibition of this enzyme activity and a lower IC_50_ value. Furthermore, the analyzed compounds are not selective. They also inhibit, albeit to a lesser extent (in the range from 19.34 to 32.55% at a concentration of 10 μM), the activity of isoform 2 of the enzyme (11β-HSD2). However, carborane derivatives are a group of compounds that have not been previously tested in the literature for 11β-HSD1 inhibition. Therefore, the fact that all analyzed compounds showed significant inhibitory activity in relation to this enzyme isoform, and less activity in relation to isoform 2, demonstrates the possibility of searching for a selective 11β-HSD1 inhibitor in this group of compounds.

Summarizing the structure–activity relationship (SAR), it can be concluded that the obtained values of inhibition of 11β-HSD1 are not very dependent on the type of substituent and are very similar to each other. However, the best activity is observed for the compounds with electron-withdrawing substituents (-Cl, -Br, -NO_2_) for compounds **4a**, **4b** and **4g** (IC_50_ = 6.90–7.05 mM), while the second group, containing substituents such as -F and -diCl, including compounds **4c** and **4e** (IC_50_ = 7.90–8.10), is characterized by slightly lower activity, probably due to the high electronegativity of these substituents. However, the lowest activity was shown by compounds **4d** and **4f** containing substituents with electron-donating groups (-CH_3_, -OCH_3_), with IC_50_ = 9.05 mM and 9.35 mM, respectively.

### 2.5. Molecular Docking Study

**Figure 2:**
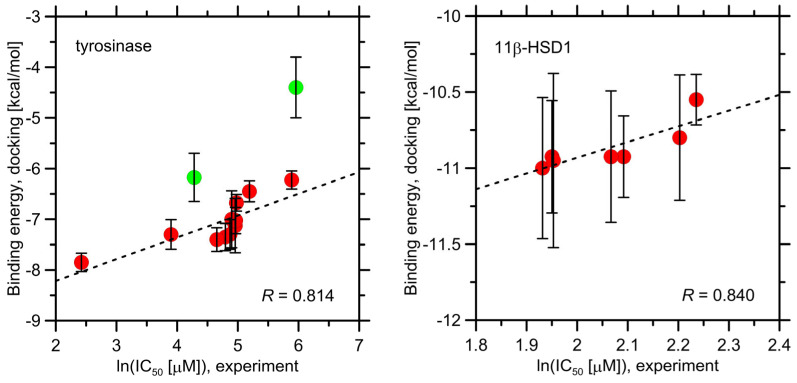
The correlation between the binding energies calculated during docking studies for a series of ligands to the two studied proteins and the experimental values of ln(IC_50_). The vertical bars denote the standard deviation values calculated with respect to various crystal structures of the same protein. The ligands containing the *o*-carborane moiety are represented by red dots whereas kojic acid and ascorbic acid by green dots. The corresponding results for carbenoxolone are not shown due to magnitude mismatch.

The binding energies found during docking simulations are given in Table 4 and graphically illustrated in Figure 2. In the case of tyrosinase, apart from the two ligands that do not belong to the *o*-carborane family (i.e., ascorbic acid and kojic acid), the relatively limited magnitude of the obtained binding energies can be noticed (−6.2–−7.9 kcal/mol). The strongest binding is exhibited by **4h**, whereas the weakest is displayed by **4i** and **4j**, which correctly reflects the limiting behavior found in the experimental studies. More quantitative analysis reveals that the binding energies calculated for *o*-carborane derivatives are strongly correlated with the experimentally determined values of ln(IC_50_) (see Figure 2). The ascorbic acid and kojic acid do not follow this trend, probably due to divergent molecular topologies when compared to the *o*-carborane ligands, which enhances the possible docking-related errors. Nevertheless, the docking results still correctly predict the weakest potency of ascorbate ions in relation to all remaining ligands. In the case of 11β-HSD1, the scatter of the obtained binding energy values is even narrower (−11.0–−10.6 kcal/mol), which partially results from the smaller pool of considered compounds. As in the previous case, the theoretically determined binding energies correlate reasonably well with the experimental ln(IC_50_) values, including the correct predictions of the most (**4a**, **4b** and **4g**) and least potent (**4f**) compounds. However, one can note that this apparently good correlation is a consequence of slightly distinct binding energies obtained for **4f** and **4d**, whereas the remaining compounds display extremely similar affinities (see Table 4). Carbenoxolone, exhibiting a unique molecular structure in comparison to remaining ligands, differs also with respect to the magnitude of determined binding energy (Table 4) and does not follow the above-described correlation.

In summary, one can conclude that a reasonably good agreement between the theoretical and experimental, affinity-related results exists.

**Figure 3:**
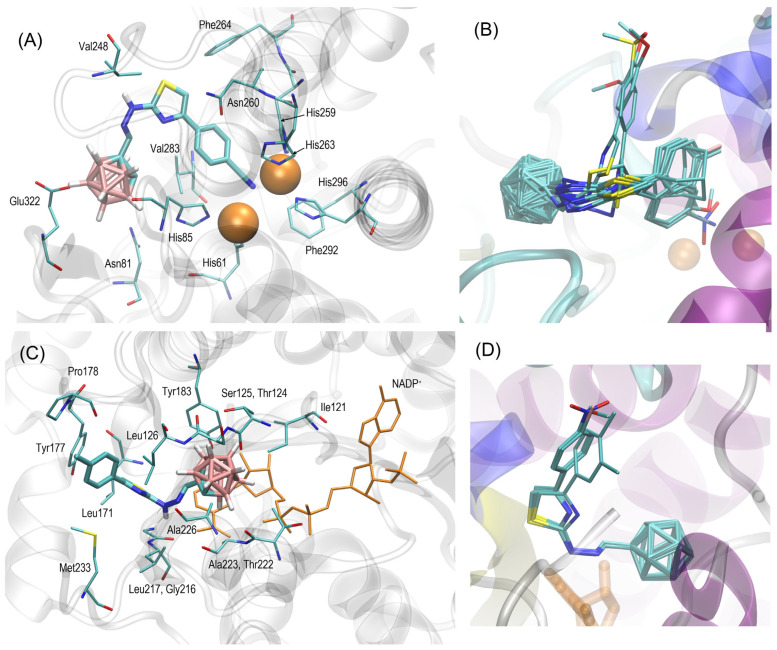
(**A**) The location of the **4h** ligand molecule bound to the tyrosinase (PDB:2y9x) structure and found in the docking procedure. The ligand molecule is shown as thick sticks, whereas all the closest amino acid residues (of a distance no larger than 0.38 nm) are represented by thin sticks. Copper ions are shown as orange balls. The description of the interaction types is given in the text. (**B**) The superposition of the most favorable poses of all ligands in the tyrosinase-containing complexes. Compounds **4i** and **4j** exhibit the orientation in the binding cavity, which is notably different from those of remaining ligands; compounds **4f**, **4g**, and **4h** are located in the closest vicinity of Cu ions present in the protein structure. (**C**) The location of the **4a** ligand molecule bound to the 11β-HSD1 (PDB:3czr) structure and found in the docking procedure. The NADP^+^ molecule is highlighted in orange. Other details as in (**A**). (**D**) The superposition of the most favorable poses of ligands **4a**–**4g** in the 11β-HSD1-containing complexes.

The results of the docking studies were analyzed with respect to the mechanistic interaction pattern that may be significant in the context of interpretation of the obtained binding energies. The summary given below relies on analyzing the ligand–protein contacts that take place if the distance between any corresponding atom pair is smaller than the arbitrarily accepted value of 0.38 nm. The description provided below concerns (1) in the case of tyrosinase, the most potent compound (**4h**), and (2) in the case of 11β-HSD1, compound **4a**. A similar interaction pattern is seen for the given protein and most of the studied carborane–thiazole derivatives; the exceptions are compounds **4i** and **4j** interacting with tyrosinase. The graphical illustration of the docking results (on example of **4h** and **4a**) is given in Figure 3.

The tyrosinase-containing complexes are considered in the first order. The most favorable binding mode is correlated with the intensive interactions of the nucleophilic substituent of the phenyl group (e.g., the –CN moiety) with the Cu ions present in the protein structure. The corresponding interatomic distances are close to the van der Waals radii; thus, a strong contribution of the electrostatic interactions or, possibly, coordination bonding can be expected to play a role in the ligand–protein interactions. Additionally, the presence of cluster composed of positively charged sidechains of histidines (His259, His263, His61, His85, and His296) favorably influences the interactions of nucleophilic substituents attached to the phenyl ring. The phenyl group interacts with Val283 and Asn260 via the π-stacking interactions involving either the aliphatic hydrogen atoms of Val or the lone electron pairs of Asn. The thiazole ring, being the central part of the ligand molecule, is in contact with Asn260 and Phe264. The latter interactions, driven by the CH–π forces, is characteristic also of other ligands containing a thiazole moiety [39]. Surprisingly, the *o*-carborane group is not involved in any type of non-polar interactions. Instead, it maintains close contact with the hydrophilic sidechains of Glu322, Asn81, and the backbone fragment of His85. Moreover, this part of the ligand molecule adopts a fixed position across the whole set of docked molecules, indicating the conserved character of the involved interactions.

When considering the complete series of ligands, the same pattern of interactions with tyrosinase as that discussed above can be observed for **4g** and **4f**, i.e., ligands that, together with **4h**, display the highest values of binding energy (−7.3–−7.9 kcal/mol). All of these ligands bear nucleophilic substituents of relatively small dimensions attached to the phenyl ring. The role of the –CN group is played by either the –NO_2_ or -OMe moieties instead. The ligands with moderate binding energies exhibit a slight reorientation of the phenyl (or aliphatic, in the case of **4k**) group, which is moved further from the Cu ions and closer to Phe264. Finally, ligands with the smallest binding energies (**4i** and **4j**) are reoriented in such a way that the phenyl moiety and its substituent are located closer to Phe264 and Val248, whereas the interactions with Cu ions are less intensive due to a much larger (>0.4 nm) ligand–ion distance. We speculate that such drastic reorientation is the result of larger dimensions of the corresponding substituents which cannot be accommodated within the relatively narrow subcavity created by Cu ions and neighboring histidines.

In the case of ligands interacting with 11β-HSD1, the most intensive contacts are observed in the region of the *o*-carborane group. This group maintains contacts with the aromatic ring and furanose residue of the NADP+ molecule, also present in the 11β-HSD1 structure, as well as with sidechains of Ala223, Ala226, Thr222, Ile121, and Tyr183. Moreover, this interaction also involves backbone fragments of residues 124–126. The chemical character of the abovementioned residues is different, however; the non-polar contacts seem to be preferred, in contrast with the results found for tyrosinase. The amine group of ligands is located close to the backbone carbonyl atoms of Ala226 and Gly216, which indicates the possibility of hydrogen bonding. The thiazole ring interacts via CH–π or π–π forces with Leu171, Leu126, Leu217, and Tyr177. Some parts of these residues (namely Leu126, Tyr177, and Leu171) are also involved in the aromatic ring of the ligand molecule. Moreover, additional amino acids display contacts with this group: Met233 and Pro178. For sterical reasons, it can also be expected that this region of the ligand molecule can interact with water, which is not explicitly present in our system. Overall, the intensity of the interactions in which the ring substituents differing between compounds are involved is rather moderated and mainly includes Tyr177 and Pro178 (and possibly a solvent). This may explain the narrow range within which the binding energies vary. Finally, it is worth noting that the scatter of the ligand position in the binding cavity is much smaller than in the case of tyrosinase; this may result from both the smaller pool of considered compounds but also from the location of the crucial substituent which interacts with the protein less intensively when compared to the remaining parts of the molecule.

## 3. Materials and Methods

### 3.1. Chemistry

All reactions were performed under an argon atmosphere. All reagents and starting materials were purchased from commercial suppliers and used without further purification. The dichloromethane was dried over calcium hydride. ^1^H NMR (400 MHz) and ^13^C NMR (100 MHz) spectra were recorded on a Bruker Avance III multinuclear instrument. High-resolution mass spectrometry (HRMS) measurements were performed using a Synapt G2-Si mass spectrometer (Waters) equipped with an ESI source and quadrupole-Time-of-Flight mass analyzer. The mass spectrometer was operated in the positive- or negative-ion detection modes. To ensure accurate mass measurements, data were collected in centroid mode, and the mass was corrected during acquisition using a leucine encephalin solution as an external reference, Lock-SprayTM, (Waters Corp., Milford, MA, USA), which generated reference ions at *m*/*z* 554.2615 ([M − H]^−^) in negative-ESI mode and at *m*/*z* 556.2771 Da ([M + H]^+^) in positive-ESI mode. The results of the measurements were processed using the MassLynx 4.1 software (Waters). Melting points were determined in open glass capillaries and were uncorrected. Analytical TLC was performed using Macherey-Nagel Polygram Sil G/UV_254_ 0.2 mm plates. The malonyl dichloride and appropriate thioureas were commercial materials (Merck, Darmstadt, Germany).

#### 3.1.1. C-Formyl-*o*-Carborane (**2**)

*o*-Carborane (144 mg, 1 mmol) was dissolved in dry diethyl ether (3 mL) at room temperature and cooled to −78 °C under an argon atmosphere. To the stirred solution of *o*-carborane was slowly added *n*-BuLi (0.69 mL, 1.1 mmol, 1.6 M in hexane) over 10 min and stirring was continued for another 2.5 h at the same temperature. Methyl formate (0.22 mL, 3.6 mmol) was added to the reaction mixture, and stirring was continued for an additional 2 h. The reaction was quenched with dilute HCl (4%, 1.8 mL) at −78 °C and warmed to room temperature. Excess ether was removed under reduced pressure, the aqueous layer was extracted with hexane (3 × 15 mL), and the combined organic layers were dried over MgSO_4_. Subsequently, the solvent was evaporated to dryness under vacuum, and the product was purified by means of column chromatography on silica gel (230–400 mesh) using 100% hexane and a gradient of CHCl_3_ in hexane (0–50%) to afford a pure product, yield 0.127 g, 74%, eluent (25% CH_2_Cl_2_/hexane − 3×), R_f_ = 0.52 [42].

#### 3.1.2. 2-(1,2-Dicarba-*closo*-Dodecaboranylmethylene)Hydrazinecarbothioamide (**3**)

Thiosemicarbazide (0.292 g, 3.20 mmol) was added to a stirred solution of C-formyl-*o*-carborane (**2**) (0.547 g, 3.20 mmol) in absolute ethyl alcohol (20 mL) under an argon atmosphere and then acetic acid (0.2 mL) was added. The reaction mixture was stirred under reflux for 20 h. The product was filtered off to yield 0.69 g, 89%; mp 195–197 °C; eluent: dichloromethane/methanol (95:15), R_f_  =  0.58. ^1^H NMR (DMSO-d_6_, 400 MHz); δ (ppm); 1.42–2.90 (bs, 10H, 10B-H); 5.83 (bs, 1H, CH-carborane); 7.45 (s, 1H, CH = N); 8.08 (s, 1H, NH); 8.49 (s, 1H, NH); 11.58 (bs, 1H, NH). ^13^C NMR (DMSO-d_6_, 100 MHz), δ (ppm): 59.15 (C); 72.32 (C); 133.97 (C); 179.07 (C). ^11^B NMR (DMSO-d_6_, 224 MHz); δ (ppm); −2.52; −4.00; −9.07; −12.16.

#### 3.1.3. 4-(4-Chlorophenyl)-2-(2-(1,2-Dicarba-*closo*-Dodecaboranylmethylene)Hydrazinyl)Thiazole (**4a**)—Typical Procedure

2-(1,2-Dicarba-*closo*-dodecaboranylmethylene)hydrazinecarbothioamide (**3**) (0.086 g, 0.35 mmol) was added under an argon atmosphere to a stirred solution of 2-bromo-1-(4-chlorophenyl)ethanone (0.082 g, 0.35 mmol) in absolute ethyl alcohol (10 mL). The reaction mixture was stirred under reflux for 20 h. Next, the reaction mixture was added to water (50 mL) and neutralized with NaHCO_3_ solution. The separate precipitate was collected by means of filtration and dried in vacuum to afford the desired product: 0.120 g, 90%; mp 218–220 °C, eluent: dichloromethane/methanol (95:5); R_f_ = 0.93. ^1^H NMR (DMSO-d_6_, 400 MHz); δ (ppm); 1.37–3.09 (bs, 10H, 10B-H); 5.44 (bs, 1H, CH-carborane); 7.42–7.49 (m, 4H, 4CH); 7.84 (d, 2H, 2CH, J = 8.4 Hz); 12.57 (bs, 1H, NH). ^13^C NMR (DMSO-d_6_, 100 MHz), δ (ppm): 61.60 (C); 73.16 (C); 106.15 (C); 127.70 (2C); 129.13 (2C); 132.58 (C); 132.94 (C); 133.60 (C); 149.63 (C); 167.99 (C). ^11^B NMR (DMSO-d_6_, 224 MHz); δ (ppm); −3.13; −9.71; −11.96. ESI-HRMS (*m*/*z*) average mass for C_12_H_19_B_10_ClN_3_S: 382.1951 [M + H]^+^. Found: 382.1946 [M + H]^+^.

#### 3.1.4. 4-(4-Bromophenyl)-2-(2-(1,2-Dicarba-*closo*-Dodecaboranylmethylene)Hydrazinyl)Thiazole (**4b**)

Yield: 0.14 g, 93%, (dichloromethane/methanol (95:5), R_f_ = 0.75); mp 223–225 °C. ^1^H NMR (DMSO-d_6_, 400 MHz); δ (ppm); 1.45–3.10 (bs, 10H, 10B-H); 5.44 (bs, 1H, CH-carborane); 7.43 (s, 1H, CH); 7.48 (s, 1H, CH); 7.59 (d, 2H, 2CH, J = 8.8 Hz); 7.78 (d, 2H, 2CH, J = 8.8 Hz); 12.44 (bs, 1H, NH). ^13^C NMR (DMSO-d_6_, 100 MHz), δ (ppm): 61.60 (C); 73.15 (C); 106.24 (C); 121.17 (C); 128.02 (2C); 132.05 (2C); 132.97 (C); 133.95 (C); 149.71 (C); 168.01 (C). ^11^B NMR (DMSO-d_6_, 224 MHz); δ (ppm); −2.52; −4.04; −9.32; −11.05; −12.29. ESI-HRMS (*m*/*z*) average mass for C_12_H_19_B_10_BrN_3_S: 426.1460 [M + H]^+^. Found: 426.1454 [M + H]^+^.

#### 3.1.5. 2-(2-(1,2-Dicarba-*closo*-Dodecaboranylmethylene)Hydrazinyl)-4-(4-Fluorphenyl)Thiazole (**4c**)

Yield: 0.12 g, 94%, (dichloromethane/methanol (95:5), R_f_ = 0.87); mp 237–238 °C. ^1^H NMR (DMSO-d_6_, 400 MHz); δ (ppm); 1.43–2.94 (bs, 10H, 10B-H); 5.44 (bs, 1H, CH-carborane); 7.23 (t, 2H, 2CH, J = 9.2 Hz); 7.38 (s, 1H, CH); 7.42 (s, 1H, CH); 7.86 (q, 2H, 2CH, J = 9.2 Hz); 12.42 (bs, 1H, NH). ^13^C NMR (DMSO-d_6_, 100 MHz), δ (ppm): 61.63 (C); 73.18 (C); 105.09 (C); 115.94 (d, 2C, J_C-F_ = 21.6 Hz); 128.00 (d, 2C, J_C-F_ = 7.6 Hz); 131.34 (d, C, J_C-F_ = 2.9 Hz); 132.90 (C); 149.79 (C); 162.13 (d, C, J_C-F_ = 243.8 Hz); 167.96 (C). ^11^B NMR (DMSO-d_6_, 224 MHz); δ (ppm); −3.19; −9.83; −12.51. ESI-HRMS (*m*/*z*) average mass for C_12_H_19_B_10_FN_3_S: 366.2235 [M + H]^+^. Found: 366.2230 [M + H]^+^.

#### 3.1.6. 2-(2-(1,2-Dicarba-*closo*-Dodecaboranylmethylene)Hydrazinyl)-4-p-Tolylthiazole (**4d**)

Yield: 0.10 g, 79%, (dichloromethane/methanol (95:5), R_f_ = 0.85); mp 249–251 °C. ^1^H NMR (DMSO-d_6_, 400 MHz); δ (ppm); 1.42–3.06 (bs, 10H, 10B-H); 2.31 (s, 3H, CH_3_); 5.44 (bs, 1H, CH-carborane); 7.20 (d, 2H, 2CH, J = 8.4 Hz); 7.30 (s, 1H, CH); 7.43 (s, 1H, CH); 7.71 (d, 2H, 2CH, J = 8.4 Hz); 12.42 (bs, 1H, NH). ^13^C NMR (DMSO-d_6_, 100 MHz), δ (ppm): 21.26 (C); 61.64 (C); 73.24 (C); 104.36 (C); 125.97 (2C); 129.67 (2C); 132.03 (C); 132.84 (C); 137.51 (C); 150.72 (C); 167.85 (C). ^11^B NMR (DMSO-d_6_, 224 MHz); δ (ppm); −2.52; −4.00; −9.20; −11.19; −12.46. ESI-HRMS (*m*/*z*) average mass for C_13_H_22_B_10_N_3_S: 362.2487 [M + H]^+^. Found: 362.2484 [M + H]^+^.

#### 3.1.7. 2-(2-(1,2-Dicarba-*closo*-Dodecaboranylmethylene)Hydrazinyl)-4-(3,4-Dichlorophenyl)Thiazole (**4e**)

Yield: 0.12 g, 83%, (dichloromethane/methanol (95:5), R_f_ = 0.74); mp 239–241 °C. ^1^H NMR (DMSO-d_6_, 400 MHz); δ (ppm); 1.49–2.99 (bs, 10H, 10B-H); 5.45 (bs, 1H, CH-carborane); 7.45 (s, 1H, CH); 7.62 (s, 1H, CH); 7.66 (d, 1H, CH, J = 8.4 Hz); 7.81 (dd, 1H, CH, J_1_ = 2.0 Hz J_2_ = 8.4 Hz); 8.05 (d, 1H, CH, J = 2.4 Hz); 12.52 (bs, 1H, NH). ^13^C NMR (DMSO-d_6_, 100 MHz), δ (ppm): 61.57 (C); 73.10 (C); 107.60 (C); 126.03 (C); 127.62 (C); 130.36 (C); 131.35 (C); 131.94 (C); 133.16 (C); 135.31 (C); 148.35 (C); 168.11 (C). ^11^B NMR (DMSO-d_6_, 224 MHz); δ (ppm); −2.48; −3.94; −9.16; −11.07; −12.45. ESI-HRMS (*m*/*z*) average mass for C_12_H_18_B_10_Cl_2_N_3_S: 416.1567 [M + H]^+^. Found: 416.1562 [M + H]^+^.

#### 3.1.8. 2-(2-(1,2-Dicarba-*closo*-Dodecaboranylmethylene)Hydrazinyl)-4-(4-Methoxyphenyl)Thiazole (**4f**)

Yield: 0.13 g, 99%, (dichloromethane/methanol (95:5), R_f_ = 0.67); mp 228–230 °C. ^1^H NMR (DMSO-d_6_, 400 MHz); δ (ppm); 1.70–2.70 (bs, 10H, 10B-H); 3.75 (s, 3H, CH_3_); 5.41 (bs, 1H, CH-carborane); 6.93 (d, 2H, 2CH, J = 8.4 Hz); 7.18 (s, 1H, CH); 7.40 (s, 1H, CH); 7.72 (d, 2H, 2CH, J = 8.4 Hz); 12.17 (bs, 1H, NH). ^13^C NMR (DMSO-d_6_, 100 MHz), δ (ppm): 55.63 (C); 61.63 (C); 73.24 (C); 103.12 (C); 114.52 (2C); 127.38 (2C); 127.48 (C); 132.86 (C); 150.43 (C); 159.43 (C); 167.81 (C). ^11^B NMR (DMSO-d_6_, 224 MHz); δ (ppm); −2.58; −4.09; −9.21; −11.15; −12.42. ESI-HRMS (*m*/*z*) average mass for C_13_H_22_B_10_N_3_OS: 378.2437 [M + H]^+^. Found: 378.2429 [M + H]^+^.

#### 3.1.9. 2-(2-(1,2-Dicarba-*closo*-Dodecaboranylmethylene)Hydrazinyl)-4-(4-Nitrophenyl)Thiazole (**4g**)

Yield: 0.12 g, 88%, (dichloromethane/methanol (95:5), R_f_ = 0.85); mp 248–249 °C. ^1^H NMR (DMSO-d_6_, 400 MHz); δ (ppm); 1.70–2.71 (bs, 10H, 10B-H); 5.43 (bs, 1H, CH-carborane); 7.42 (s, 1H, CH); 7.77 (s, 1H, CH); 8.06 (d, 2H, 2CH, J = 9.1 Hz); 8.25 (d, 2H, 2CH, J = 9.1 Hz); 12.56 (bs, 1H, NH). ^13^C NMR (DMSO-d_6_, 100 MHz), δ (ppm): 61.56 (C); 73.05 (C); 110.18 (C); 124.58 (2C); 126.84 (2C); 133.30 (C); 140.73 (C); 146.78 (C); 148.91 (C); 168.31 (C). ^11^B NMR (DMSO-d_6_, 224 MHz); δ (ppm); −3.12; −9.96; −12.36. ESI-HRMS (*m*/*z*) average mass for C_12_H_19_B_10_N_4_O_2_S: 393.2181 [M + H]^+^. Found: 393.2172 [M + H]^+^.

#### 3.1.10. 4-(2-(2-(1,2-Dicarba-*closo*-Dodecaboranylmethylene)Hydrazinyl)Thiazol-4-yl)Benzonitrile (**4h**)

Yield: 0.10 g, 77%, (dichloromethane/methanol (95:5), R_f_ = 0.70); mp 244–246 °C. ^1^H NMR (DMSO-d_6_, 400 MHz); δ (ppm); 1.69–2.80 (bs, 10H, 10B-H); 5.42 (bs, 1H, CH-carborane); 7.41 (s, 1H, CH); 7.69 (s, 1H, CH); 7.84 (d, 2H, 2CH, J = 8.4 Hz); 7.98 (d, 2H, 2CH, J = 8.4 Hz); 12.53 (bs, 1H, NH). ^13^C NMR (DMSO-d_6_, 100 MHz), δ (ppm): 61.57 (C); 73.07 (C); 109.21 (C); 110.25 (C); 119.38 (C); 126.59 (2C); 133.08 (C); 133.19 (2C); 138.85 (C); 149.24 (C); 168.19 (C). ^11^B NMR (DMSO-d_6_, 224 MHz); δ (ppm); −3.13; −9.78; −12.37. ESI-HRMS (*m*/*z*) average mass for C_13_H_19_B_10_N_4_S: 373.2283 [M + H]^+^. Found: 373.2272 [M + H]^+^.

#### 3.1.11. 2-(2-(1,2-Dicarba-*closo*-Dodecaboranylmethylene)Hydrazinyl)-4-(3,4,5-Trimethoxyphenyl)Thiazole (**4i**)

Yield: 0.14 g, 92%, (dichloromethane/methanol (95:5), R_f_ = 0.63); mp 192–194 °C. ^1^H NMR (DMSO-d_6_, 400 MHz); δ (ppm); 1.64–2.70 (bs, 10H, 10B-H); 3.65 (s, 3H, CH_3_); 3.79 (s, 6H, 2CH_3_); 5.42 (bs, 1H, CH-carborane); 7.10 (s, 2H, 2CH); 7.37 (s, 1H, CH); 7.39 (s, 1H, CH); 12.53 (bs, 1H, NH). ^13^C NMR (DMSO-d_6_, 100 MHz), δ (ppm): 56.35 (2C); 60.55 (C); 61.61 (C); 73.24 (C); 103.41 (2C); 104.89 (C); 130.37 (C); 132.75 (C); 137.77 (C); 150.55 (C); 153.49 (2C); 167.62 (C). ^11^B NMR (DMSO-d_6_, 224 MHz); δ (ppm); −2.59; −4.14; −9.22; −11.14; −12.52. ESI-HRMS (*m*/*z*) average mass for C_15_H_26_B_10_N_3_O_3_S: 438.2652 [M + H]^+^. Found: 438.2652 [M + H]^+^.

#### 3.1.12. 4-(2-(2-(1,2-Dicarba-*closo*-Dodecaboranylmethylene)Hydrazinyl)Thiazol-4-yl)Benzene-1-Sulfonyl Fluoride (**4j**)

Yield: 0.13 g, 93%, (dichloromethane/methanol (95:5), R_f_ = 0.68); mp 216–218 °C. ^1^H NMR (DMSO-d_6_, 400 MHz); δ (ppm); 1.28–3.15 (bs, 10H, 10B-H); 5.43 (bs, 1H, CH-carborane); 7.43 (s, 1H, CH); 7.84 (s, 1H, CH); 8.11–8.21 (m, 4H, 4CH); 12.60 (bs, 1H, NH). ^13^C NMR (DMSO-d_6_, 100 MHz), δ (ppm): 61.61 (C); 73.02 (C); 110.94 (C); 127.31 (2C); 129.58 (2C); 133.40 (C); 141.88 (C); 148.58 (C); 168.39 (C). ^11^B NMR (DMSO-d_6_, 224 MHz); δ (ppm); −3.05; −9.87; −12.38. ESI-HRMS (*m*/*z*) average mass for C_12_H_19_B_10_FN_3_O_2_S_2_: 430.1857 [M + H]^+^. Found: 430.1852 [M + H]^+^.

#### 3.1.13. 4-(Adamant-1-yl)-2-(2-(1,2-Dicarba-*closo*-Dodecaboranylmethylene)Hydrazinyl)Thiazole (**4k**)

Yield: 0.09 g, 60%, (dichloromethane/methanol (95:5), R_f_ = 0.84); mp > 260 °C. ^1^H NMR (DMSO-d_6_, 400 MHz); δ (ppm); 1.84–2.42 (bs, 10H, 10B-H); 1.63–1.71 (m, 6H, 3CH_2_); 1.79–1.81 (m, 6H, 3CH_2_); 1.96–1.99 (m, 3H, 3CH); 5.35 (bs, 1H, CH-carborane); 6.34 (s, 1H, CH); 7.30 (s, 1H, CH); 12.31 (bs, 1H, NH). ^13^C NMR (DMSO-d_6_, 100 MHz), δ (ppm): 28.29 (3C); 36.28 (3C); 36.70 (3C); 41.59 (C); 61.51 (C); 73.26 (C); 101.28 (C); 133.50 (C); 160.19 (C); 168.31 (C). ^11^B NMR (DMSO-d_6_, 224 MHz); δ (ppm); −2.58; −4.09; −9.23; −11.18; −12.49. ESI-HRMS (*m*/*z*) average mass for C_16_H_30_B_10_N_3_S: 406.3117 [M + H]^+^. Found: 406.3113 [M + H]^+^.

### 3.2. Biological Activity

#### 3.2.1. Antiproliferative Activity

All cell lines were obtained from ATCC, and all chemicals were purchased from Sigma-Aldrich, St. Louis, MO, USA. Briefly, cells were cultivated in a controlled atmosphere using DMEM medium with fetal bovine serum (FBS) at a final concentration of 10% (A-172), an L15 medium with 15% FBS, cultivated without CO_2_ (MDA-MB-231), F12K with a heparin final concentration of 0.1 mg/mL, endothelial cell growth supplement with a final concentration of 0.03 mg/mL (HUVEC), and RPMI with 5% FBS. All media were supplemented with antibiotic and antimycotic solutions, according to the manufacturers’ instructions. MTT cytotoxicity assessments were performed as described previously [40,41]. Briefly, cell lines A172, B16F10, MDA-MB-231, and HUVEC were seeded 24 h prior to the addition of the tested compounds. The cells counted using an automated cell counter (Countess^®^ II FL, Invitrogen by Thermo Fisher Scientific Inc., Waltham, MA, USA) were plated in 96-well plates (Nunc Edge 2F, Thermo Fisher Scientific Inc.) at a density of 0.1 × 10^4^ per well. After an additional 72 h, cells underwent an MTT cytotoxicity assay (see below). The IC_50_ values were calculated using ED50plus v 1.0 freeware software (Instituto Nacional de Enfermedades Respiratorias, Mexico City, Mexico). Each cell line was tested in three independent replicates.

Twenty microliters of an MTT solution (MTT: 3-(4,5-dimethylthiazol-2-yl)-2,5-diphenyl tetrazolium bromide, stock solution: 5 mg/mL) was added to each cell line for 2 h (A172, B16F10, MDA-MB-231, and HUVEC). After the incubation time was complete, the medium was removed and crystals of formazan were solubilized in pure isopropanol. The absorbance readout was conducted immediately using a plate reader, Multiskan Spectrum, at 570 nm (Thermo Fisher Scientific Inc., Waltham, MA, USA). The background optical density was measured in the wells filled with the culture medium, without any cells.

#### 3.2.2. Mushroom Tyrosinase Inhibition Assay

The mushroom tyrosinase (Sigma-Aldrich) inhibition was performed following previously reported methods [43]. All the assays were carried out with solutions containing phosphate buffer (50 mM, pH 6.8), L-DOPA (0.17 mM), EDTA (0.022 mM), tyrosinase (50–100 units) and varying concentrations of compounds and were performed in triplicate at room temperature. The inhibitor solutions were prepared in DMSO with an initial concentration of 1 mM. Different aliquots were added to the solution containing the buffer, L-DOPA, and EDTA, with the enzyme being added last. The formation of dopachromone was determined by monitoring the absorbance at 475 nm with a T60U spectrophotometer (PG Instruments) equipped with quartz cells of 1 cm path length. Ascorbic acid was used as a reference inhibitor with an initial concentration of 1 mM. The IC_50_ values were calculated from the equation generated via exponential fit of the experimental data. The effectiveness of inhibition was expressed for the investigated compounds as the percentage of concentration necessary to achieve 50% inhibition (IC_50_), calculated using Equation (1):

% of Inhibition = {[(B30 − B0) − (A30 − A0)]/(B30 − B0)} × 100,(1)

where B0 = absorbance of L-DOPA + tyrosinase at t = 0 min, B30 = absorbance of L-DOPA + tyrosinase at t = 30 min, A0 = absorbance of L-DOPA + tyrosinase + inhibitor at t = 0 min, and A30 = absorbance of L-DOPA + tyrosinase + inhibitor at t = 30 min.

#### 3.2.3. Kinetic Analysis of the Inhibition of Tyrosinase

A series of experiments were performed to determine the inhibition kinetics of compounds by following the already reported method [44]. The inhibitor concentrations for compounds were 50 and 100 µM. The substrate L-DOPA concentration was between 100 and 250 µM in all kinetic studies. Maximal initial velocity was determined from the initial linear portion of absorbance up to ten minutes after the addition of the enzyme. The inhibition type of the enzyme, Michaelis constant (KM), and maximal velocity (Vmax) were determined by Lineweaver–Burk plots of the inverse of velocities (1/V) versus the inverse of substrate concentration 1/[L-DOPA] mM^−1^.

#### 3.2.4. Reagents and Solvents

18-beta-Glycyrrhetinic acid was obtained from Acros Organic; phosphate buffer powder, cortisone, NADPH tetrasodium salt, and NAD Cofactor were obtained from Sigma-Aldrich; dimethylsulfoxide was obtained from POCh Poland; and carbenoxolone (sodium salt) was obtained from Cayman Chemical Company, Ann Arbor, Michigan, USA. Pooled human liver microsomes, mixed gender, 1 mL, 20 mg/mL, Lot No. 1410013, were obtained from XenoTech. Cortisol Elisa Ref DKO001, Lot No. 5148A was obtained from DiaMetra. The Enzyme-Linked Immunosorbent Assay (ELISA) Kit for 11-Beta-Hydroxysteroid Dehydrogenase Type 1, Lot No. L160706125, was obtained from Cloud-Clone Corp. PBS, Lot No. H161008, was obtained from Pan Biotech. Human kidney microsomes, mixed gender, 0.5 mL, 10 mg/mL, Lot No. 1710160, were obtained from XenoTech. The Enzyme-Linked Immunosorbent Assay (ELISA) Kit for 11-Beta-Hydroxysteroid Dehydrogenase Type 2, Lot No. L191113457, was obtained from Cloud-Clone Corp. 

#### 3.2.5. Inhibition of 11β-HSD1 Assays

The reduction reaction of cortisone to cortisol was carried out on 96-well microtiter plates in the presence of the enzyme 11β-HSD1 in a total volume of 100 µL. To this end, 20 μL of a cortisone/NADPH mixture with a final concentration of 200 nM/2 mM, 10 µL of a microsome (1.13 µg/mL 11β-HSD1) solution in PBS (final quantity 2.5 µg), 60 µL of phosphate buffer (pH 7.4), and 10 µL of the appropriate inhibitor solution (solvent DMSO/water 1/99, final concentration 10 µM) were placed in each well. The microplates were incubated on an orbital shaker for 150 min at 37 °C. The reaction was stopped via the addition of 10 µL of a solution containing 100 µM 18β-glycyrrhetinic acid in PBS. The quantity of cortisol formed from cortisone was determined using a cortisol ELISA kit, DiaMetra.

#### 3.2.6. Inhibition of 11β-HSD2 Assays

The oxidation reaction of cortisol to cortisone was carried out on 96-well microtiter plates in the presence of the enzyme 11β-HSD2 in a total volume of 100 µL. To this end, 60 µL of phosphate buffer (pH 7.4), 10 µL of a microsomes(0.12 µg/mL 11β-HSD1) solution in PBS (final quantity 2.5 µg), 20 µL of substrate mixture cortisol/NAD (final concentration 200 nM/2 mM), and 10 µL of the appropriate inhibitor solution (solvent DMSO/water 1/99, final concentration 10 µM) were placed in each well. The microplates were incubated on an orbital shaker for 150 min at 37 °C. The reaction was stopped by the addition of 10 µL solution containing 100 µM carbenoxolone in PBS. The quantity of unreacted cortisol was determined using a cortisol ELISA kit DiaMetra.

### 3.3. Molecular Docking Study

The ligand molecules were drawn by using the Avogadro 1.1.1 software [45] and initially optimized within the UFF force field [46] (5000 steps, steepest descent algorithm). Further geometrical optimization was carried out at the level of the ab initio calculations at the DFT/B3LYP/6-31G(d,p) level of theory [47,48,49] by using the Gaussian09 package [50]. Flexible, optimized ligands molecules were docked into the binding pocket of (1) the tyrosinase structures found in the 2y9x PDB entry (tyrosinase from *Agaricus bisporus*, X-ray resolution: 0.278 nm). All four protein structures present in this PDB record were used in the four independent docking procedures; (2) the 11β-HSD1 protein structures found in the following PDB entries: 3crz, 3qqp, 4bb5, 4c7j, and 4hfr. These five structures were used for five independent docking procedures.

The AutoDock Vina 1.1.2 software [51] was applied for docking simulations. Due to the lack of boron-related parameters in nearly all available docking programs, we applied the procedure proposed and validated in [52], relying on directly adopting the parameters corresponding to the carbon atoms, characterized by the same molecular topology as the boron atoms of interest. Additionally, in order to increase the accuracy, the atomic partial charges for the o-carborane moiety were assigned according to the Mulliken scheme [53], on the basis of the previous DFT calculations. This protocol was initially validated for the case of human dihydrofolate reductase complexed with 2,4-diamino-5-(1-o-carboranylmethyl)-6-methylpyrimidine (PDB: 2c2s). Both the default parameters (i.e., boron treated as carbon) and the parameters with boron-characteristic atomic charges derived from the DFT calculations provided a similar, satisfactory quality of docking. The graphical illustration of the validation results is given in the Supporting Information as Figure S1. In the context of other, non-carborane ligands, the validation methodology and the corresponding results are given in our previous works [39].

The procedure of docking was carried out within the cuboid regions of dimensions of 18 × 18 × 18 Å^3^ (tyrosinase) or 22 × 22 × 22 Å^3^ (11β-HSD1), which cover all the originally co-crystallized ligands present in the considered PDB structures as well as the closest amino acid residues that exhibit contact with those ligands. All the default procedures and algorithms implemented in AutoDock Vina were applied during docking procedure. The predicted binding energies were averaged over all four or five considered protein structures. The calculations of the RMSD value for each pose of the docked ligand (after aligning with respect to the given structure) were carried out as well in order to ensure that the partial energies contributing the final, averaged, binding energy value corresponded to the structurally analogous orientations. Only the lowest average energy values, corresponding to the most favorable complexes, were considered during subsequent analysis.

## 4. Conclusions

The presented study depicted the synthesis of 11 carborane–thiazole conjugates, as well as their in-depth evaluation involving the assessment of enzymatic activity, antiproliferative activity, and molecular docking study.

The antiproliferative activity was tested for all compounds with the use of three cancer cell lines (A172, B16F10 and MDA-MB-231) and with a healthy control cell line (HUVEC). The results suggest that the newly synthesized carborane derivatives were most active against the murine melanoma cells. Moreover, in addition to the efficacy, the selectivity (affecting the safety profile) of the evaluated compounds was also the most beneficial for the murine melanoma cells.

Furthermore, compounds **4a**–**4g** were investigated for their activity against the two isoforms of 11β-hydroxysteroid dehydrogenase, namely 11β-HSD1 and 11β-HSD2. The results show that in the same way as the reference inhibitor of carbenoxolone, the newly synthesized compounds unselectively inhibit both isoforms of the enzyme, with bias towards the 11β-HSD1 isoform. While the lowest IC_50_ value could be observed for the compound **4b**, the greatest percentage of 11β-HSD1 inhibition could be observed for the compound **4g**, which is especially advantageous.

The analysis of mushroom tyrosinase inhibition determined compounds **4h** and **4f** to be the two most active of the analyzed compounds. The recorded IC_50_ values were particularly low and surpassed the results recorded for the reference inhibitor, kojic acid, by over 6.4-fold, while they were over 34.2 times lower than the results recorded for the other reference inhibitor, ascorbic acid.

Molecular docking analysis suggested that the *o*-carborane group is not involved in any type of non-polar interactions when considering the interactions with tyrosinase. Instead, it maintains close contact with the hydrophilic sidechains of Glu322 and Asn81, as well as with the backbone fragment of His85. What is more, this fragment of molecule upholds a fixed position across the whole set of docked molecules, indicating the conserved character of interactions. In the case of the binding energy, the most important is the effect exerted by the nucleophilic substituent of the phenyl group such as the –CN moiety, which is present in the compound **4h**. This ensures the close proximity between the molecule and copper ions present within the protein, as well as preventing the unfavorable reorientation of the phenyl group with its substituent towards the Phe264 and Val248.

The pattern of interactions obtained for 11β-HSD1 is qualitatively different and indicates that less polar contacts are preferred in the case of the *o*-carborane group. The divergences in binding affinities are a consequence of interactions of various chemical characters, involving Tyr177, Pro178, and, possibly, a solvent.

Considering all the results, compound **4h** excels as the most promising carborane–thiazole conjugate and is set for further detailed evaluation. Moreover, it should be noted that the presented study demonstrated for the first time that carborane–thiazole derivatives are endowed with the ability to inhibit both tyrosinase and 11β-hydroxysteroid dehydrogenase type 1. This stands out as an unprecedented opportunity for simultaneous treatment of melanoma and comorbid diseases such as type II diabetes mellitus. This seems especially timely, as type II diabetes mellitus has recently been associated with more advanced forms of melanoma and an increased risk for melanoma recurrence [13,54,55]. These findings provide valuable insights for the rational design and optimization of novel tyrosinase and 11β-HSD1 inhibitors with enhanced therapeutic potential.

## Figures and Tables

**Table 1:** Effect of newly synthetized carborane–thiazole derivatives on the proliferation of human brain glioblastoma (A172), murine melanoma (B16F10), human breast adenocarcinoma (MDA-MB-231), and human umbilical vein endothelial cells (HUVEC) (shown as the IC_50_ values).

Thiazole Derivatives	IC_50_ ± SD [µM]				SI		
	A172	B16F10	MDA-MB-231	HUVEC	HUVEC/A172	HUVEC/ B16F10	HUVEC/ MDA-MB-231
**4a**	5.77 ± 0.96	5.03 ± 0.69	6.54 ± 0.52	6.87 ± 0.33	1.19	1.37	1.05
**4b**	6.15 ± 0.26	5.37 ± 0.11	6.56 ± 0.12	7.46 ± 0.13	1.21	1.39	1.14
**4c**	7.10 ± 1.90	5.57 ± 0.39	6.02 ± 0.08	7.51 ± 0.64	1.06	1.35	1.25
**4d**	7.88 ± 0.98	>10	6.85 ± 0.86	8.32 ± 0.09	1.06	–	1.21
**4e**	6.44 ± 0.60	5.58 ± 0.35	6.99 ± 2.27	7.86 ± 0.61	1.22	1.41	1.12
**4f**	>10	5.81 ± 0.82	7.32 ± 1.26	>10	–	>1.72	>1.37
**4g**	6.07 ± 1.06	5.11	6.96 ± 0.74	>10	>1.65	>1.96	>1.44
**4h**	7.72 ± 1.25	5.56 ± 0.30	6.64 ± 0.81	6.17 ± 0.35	0.80	1.11	0.93
**4i**	>10	5.88	>10	>10	–	>1.70	–
**4j**	6.40 ± 0.71	5.75 ± 0.46	6.43 ± 0.23	>10	>1.56	>1.74	>1.56
**4k**	>10	>10	>10	5.86 ± 0.34	–	–	–
chlorambucil	>10	5.44 ± 0.19	>10	7.43 ± 0.54	–	1.37	–

Selectivity index (SI) = IC_50_ of compound in a normal cell line/IC_50_ of the compound in cancer cell line.

**Table 2:** Inhibition activity towards the mushroom tyrosinase and enzyme inhibition kinetics of the evaluated compounds.

Compound at 0.05 mM	IC_50_ ± SD [µM]	Mechanism of Inhibition	V_max_ [mM/min]	K_M_ [mM]
**4a**	130.49 ± 20.94	Mixed	0.1656	0.4368
**4b**	144.88 ± 14.36	Activator	0.0912	0.1868
**4c**	143.09 ± 9.38	Activator	0.0810	0.9103
**4d**	120.91 ± 21.88	Mixed	0.3820	0.5246
**4e**	142.21 ± 12.98	Activator	0.1575	0.4651
**4f**	49.36 ± 8.01	Activator	0.1472	0.5206
**4g**	104.96 ± 9.08	Uncompetitive	0.0521	0.1890
**4h**	11.27 ± 5.90	Mixed	0.1258	0.5711
**4i**	179.78 ± 7.83	Mixed	0.5474	0.5868
**4j**	360.51 ± 118.59	Mixed	0.7876	0.5072
**4k**	134.11 ± 5.84	Mixed	0.7863	0.6933
Ascorbic acid	386.50 ± 11.96	―	―	―
Kojic acid	72.27 ± 3.15	―	―	―

**Table 3:** Inhibitory activity against the 11β-hydroxysteroid dehydrogenases type 1 and type 2 of the evaluated compounds.

Thiazole Derivatives	% of 11β-HSD1 Inhibition 10 μM	IC_50_ ± SD (µM) ^1^ 11*β*-HSD1	% of 11β-HSD2 Inhibition 10 μM
**4a**	60.07	7.03 ± 0.53	25.47
**4b**	64.44	6.90 ± 1.20	27.36
**4c**	55.74	8.10 ± 2.00	21.70
**4d**	55.78	9.05 ± 0.25	27.83
**4e**	62.17	7.90 ± 1.40	19.34
**4f**	55.54	9.35 ± 1.85	29.25
**4g**	73.51	7.05 ± 0.25	32.55
Carbenoxolone	88.93	<0.5	
18β-Glycyrrhetinic acid			43.40

^1^ Values were obtained with 5 inhibitor concentrations from three independent determinations at 10 µM.

**Table 4:** The binding energies recovered during docking with respect to the studied ligands. The calculation results were averaged over 4 or 5 structures available for the given type of protein; the corresponding standard deviations are given.

Compound	Binding Energy [kcal/mol]	
	Tyrosinase	11*β*-HSD1
**4a**	−7.3 ± 0.3	−10.9 ± 0.4
**4b**	−6.7 ± 0.2	−11.0 ± 0.5
**4c**	−7.0 ± 0.3	−10.9 ± 0.3
**4d**	−7.4 ± 0.3	−10.8 ± 0.4
**4e**	−7.1 ± 0.5	−10.9 ± 0.4
**4f**	−7.3 ± 0.3	−10.6 ± 0.2
**4g**	−7.4 ± 0.2	−11.0 ± 0.6
**4h**	−7.9 ± 0.2	
**4i**	−6.2 ± 0.2	
**4j**	−6.2 ± 0.2	
**4k**	−7.0 ± 0.6	
Ascorbic acid ^1^	−4.4 ± 0.6 ^1^	
Kojic acid	−6.2 ± 0.5	
Carbenoxolone		−9.2 ± 0.4

^1^ Value additionally averaged over the three tautomeric forms of ascorbate ion.

## Data Availability

The original contributions presented in this study are included in this article/the Supplementary Materials; further inquiries can be directed to the corresponding author.

## Supplementary Materials

The following supporting information can be downloaded at: https://www.mdpi.com/article/10.3390/molecules29194716/s1, Figure S1: The graphical illustration of the validation results.
