# Supplementary material for: Synthesis of Carborane–Thiazole Conjugates as Tyrosinase and 11β-Hydroxysteroid Dehydrogenase Inhibitors: Antiproliferative Activity and Molecular Docking Studies
Source: Molecules. 2024 Oct 5;29(19):4716. doi: 10.3390/molecules29194716 (PMC11477717; doi:10.3390/molecules29194716)
Supplement: Supplementary file 1 [file molecules-29-04716-s001.zip › molecules-3201712-supplementary.pdf]

Supporting Information

Synthesis of Carborane-Thiazole Conjugates as Tyrosinase and 11β-Hydroxysteroid Dehydrogenase Inhibitors: Antiproliferative Activity and Molecular Docking Studies

Beata Donarska[1], Joanna Cytarska[1], Dominika Kołodziej-Sobczak[1], Renata Studzińska[2], Daria Kupczyk[3], Angelika Baranowska-Łączkowska[4*], Karol Jaroch[5], Paulina Szeliska[5], Barbara Bojko[5], Daria Różycka[6], Agnieszka B. Olejniczak[6], Wojciech Płaziński[7,8], Krzysztof Z. Łączkowski[1,*]

1 Department of Chemical Technology and Pharmaceuticals, Faculty of Pharmacy, Collegium Medicum, Nicolaus Copernicus University, Jurasza 2, 85-089 Bydgoszcz, Poland
2 Department of Organic Chemistry, Faculty of Pharmacy, Collegium Medicum, Nicolaus Copernicus University, Jurasza 2, 85-089 Bydgoszcz, Poland
3 Department of Medical Biology and Biochemistry, Faculty of Medicine, Collegium Medicum, Nicolaus Copernicus University, Karłowicza 24, 85-092 Bydgoszcz, Poland
4 Faculty of Physics, Kazimierz Wielki University, Powstańców Wielkopolskich 2, 85-090, Bydgoszcz, Poland
5 Department of Pharmacodynamics and Molecular Pharmacology, Faculty of Pharmacy, Collegium Medicum, Nicolaus Copernicus University, Jurasza 2, 85-089 Bydgoszcz, Poland
6 Screening Laboratory, Institute of Medical Biology, Polish Academy of Sciences, Lodowa 106, 93-232 Lodz, Poland
7 Jerzy Haber Institute of Catalysis and Surface Chemistry, Polish Academy of Sciences, Niezapominajek 8, 30-239, Cracow, Poland
8 Department of Biopharmacy, Medical University of Lublin, Chodzki 4a, 20-093 Lublin, Poland

*Correspondence: Angelika Baranowska-Łączkowska, e-mail: anxela@ukw.edu.pl
Krzysztof Z. Łączkowski, e-mail: krzysztof.laczkowski@cm.umk.pl

Contents

1. Figure S1. The graphical illustration of the validation results.

2. ^{1}H, ^{13}C NMR and ESI-HRMS spectra of compounds 3 and 4a-4k.

3. The Lineweaver-Burk plots for tyrosinase inhibition for compounds 4a-4k. The secondary plots of the slope and the intercept of the straight lines versus the concentration of compound 4f i 4h.

4. The plots of % inhibition versus the log concentration of the tyrosinase inhibitors 4a-4k.

Figure S1. The graphical illustration of the validation results.

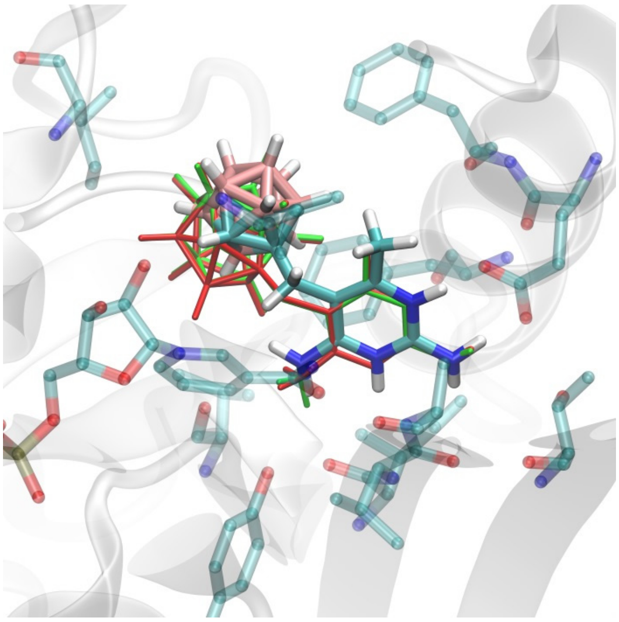

Compound 3

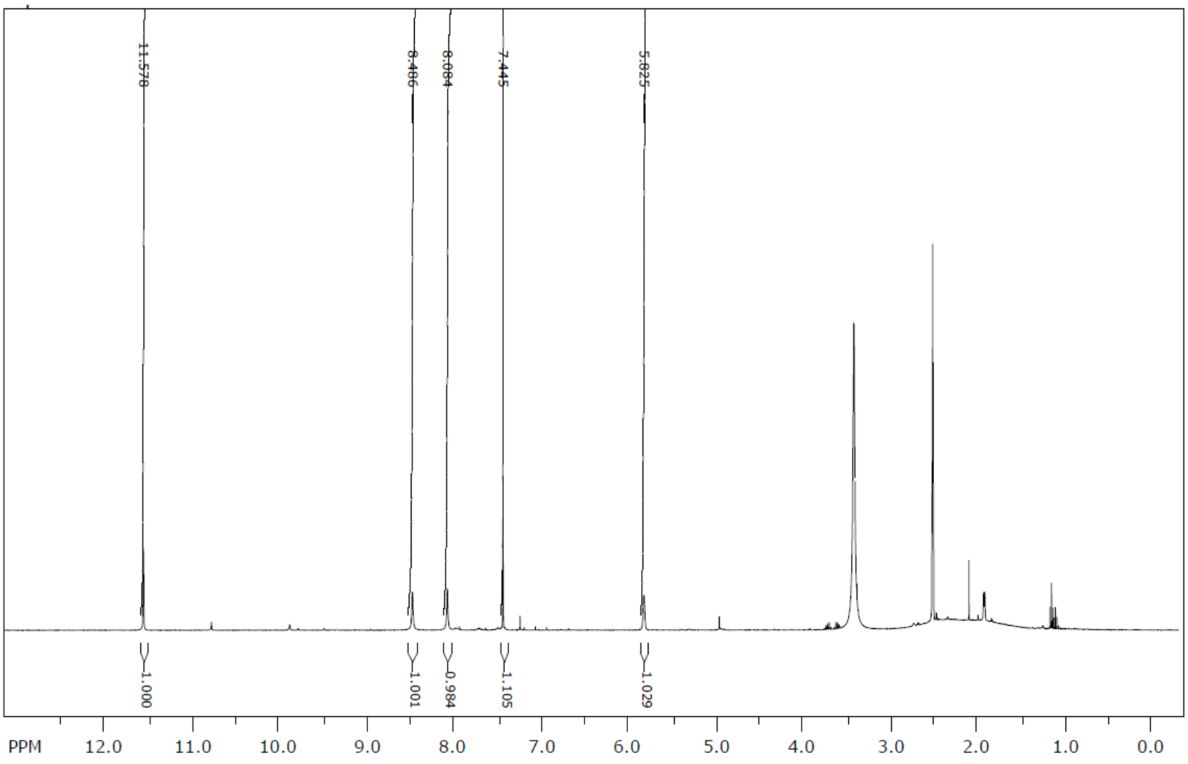

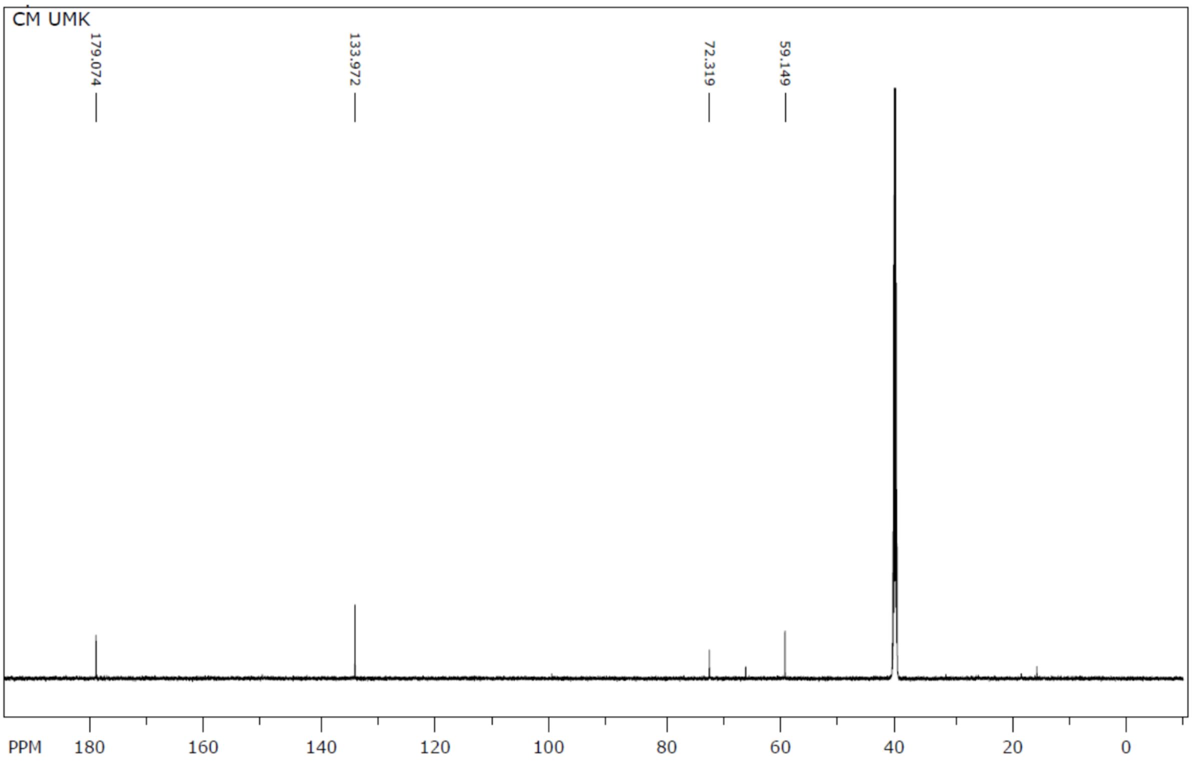
CM UMK
179.074
133.972
72.319
59.149
PPM 180 160 140 120 100 80 60 40 20 0

Compound 4a

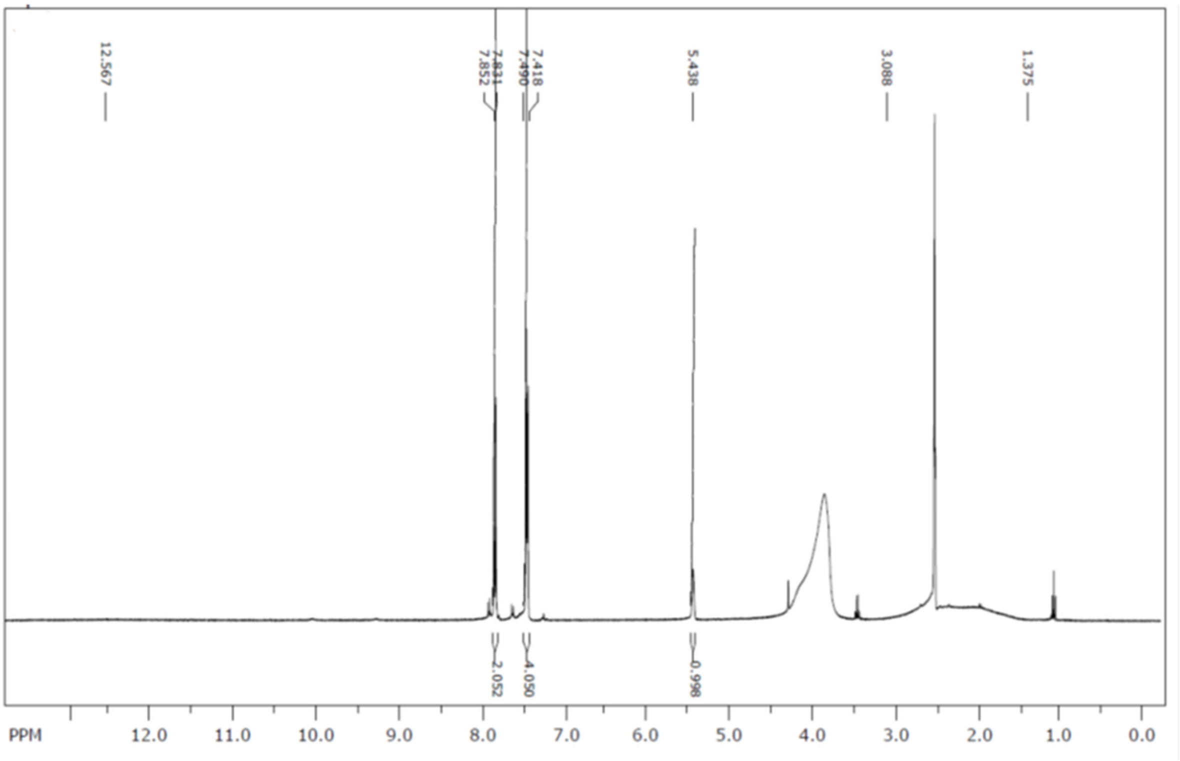
12.567
7.852
7.831
7.490
7.418
5.438
3.088
1.375
2.052
4.050
0.998
PPM 12.0 11.0 10.0 9.0 8.0 7.0 6.0 5.0 4.0 3.0 2.0 1.0 0.0

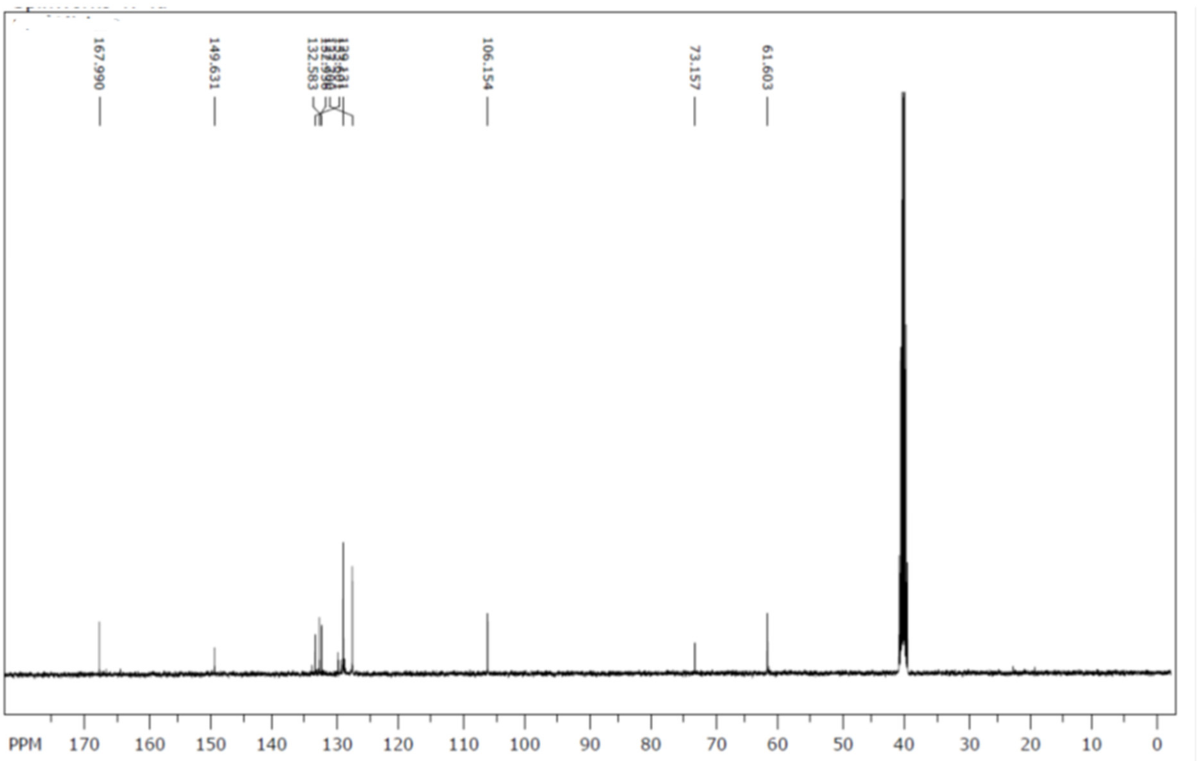
167.990
149.631
132.583
106.154
73.157
61.603
PPM 170 160 150 140 130 120 110 100 90 80 70 60 50 40 30 20 10 0

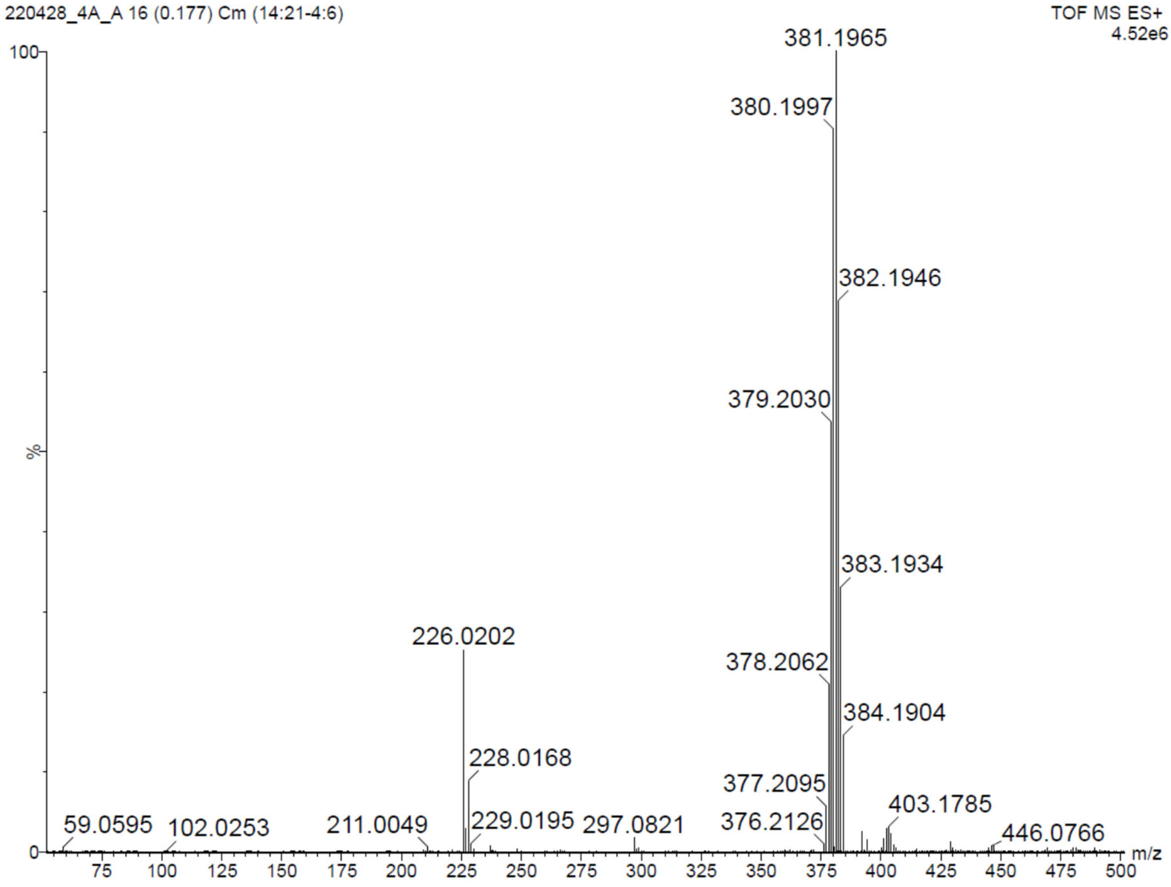
220428_4A_A 16 (0.177) Cm (14:21-4:6)
TOF MS ES+
4.52e6
381.1965
380.1997
382.1946
379.2030
383.1934
226.0202
378.2062
384.1904
228.0168
377.2095
403.1785
59.0595
102.0253
211.0049
229.0195
297.0821
376.2126
446.0766
100
%
0
75 100 125 150 175 200 225 250 275 300 325 350 375 400 425 450 475 500
m/z

Compound 4b

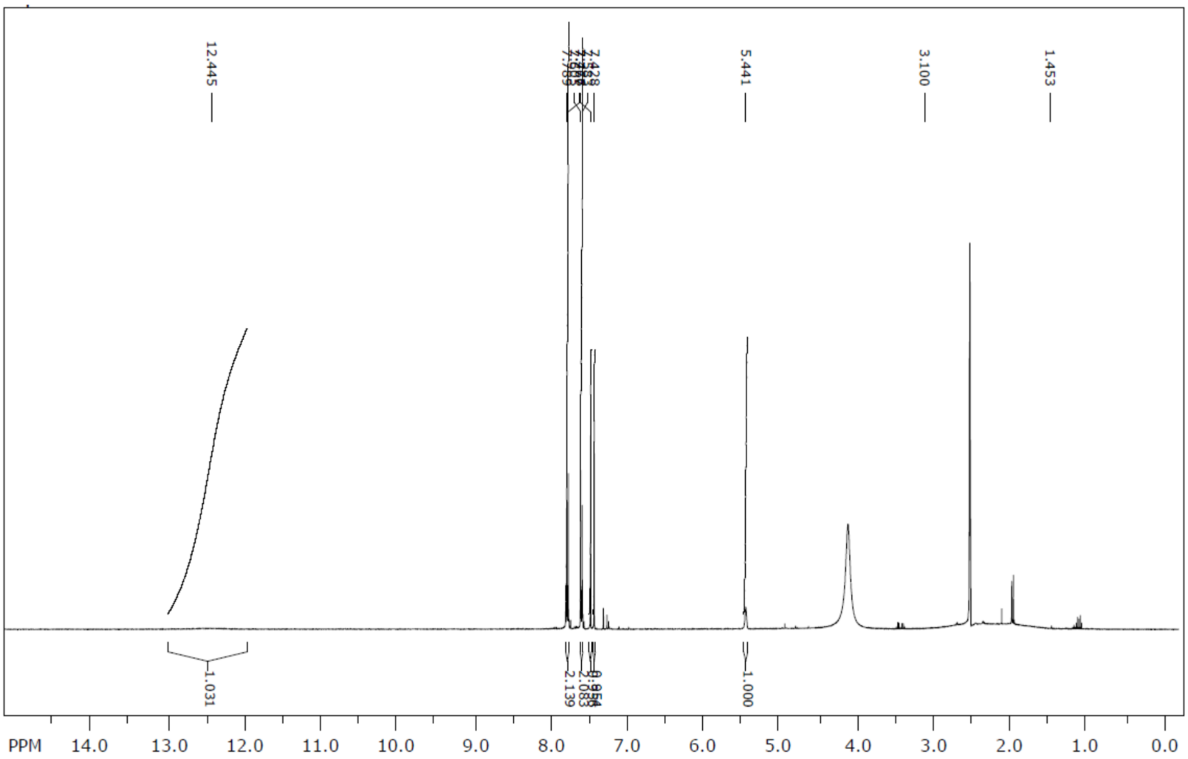
12.445
5.441
3.100
1.453
1.031
2.139
1.000
PPM 14.0 13.0 12.0 11.0 10.0 9.0 8.0 7.0 6.0 5.0 4.0 3.0 2.0 1.0 0.0

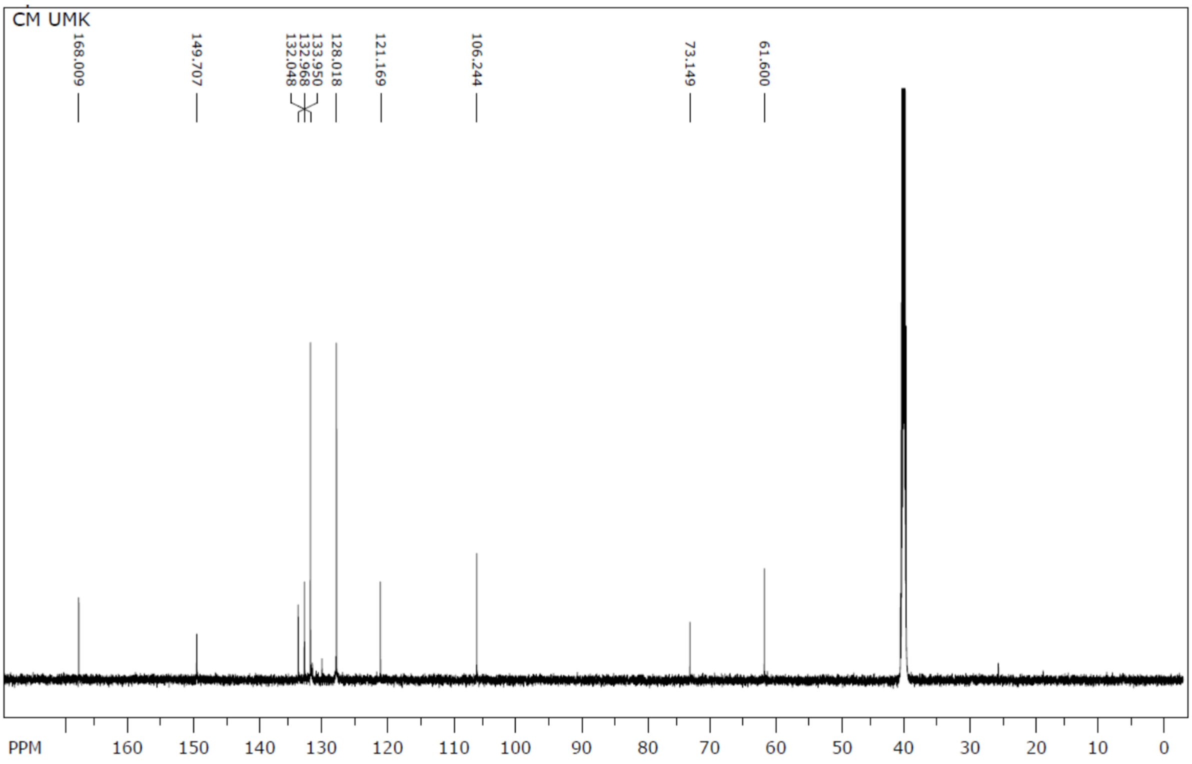
CM UMK
168.009
149.707
133.950
132.968
132.048
128.018
121.169
106.244
73.149
61.600
PPM 160 150 140 130 120 110 100 90 80 70 60 50 40 30 20 10 0

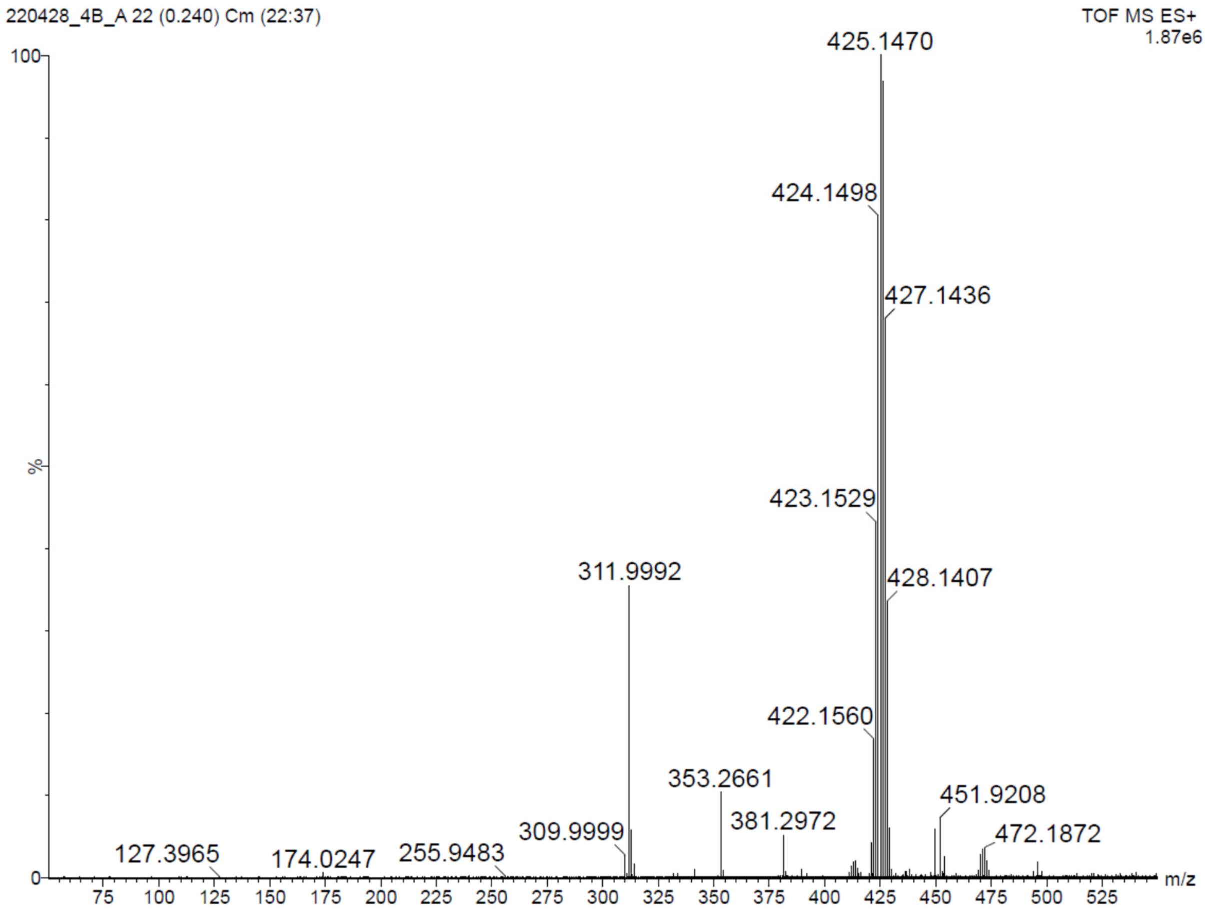
220428_4B_A 22 (0.240) Cm (22:37)
TOF MS ES+
1.87e6
425.1470
424.1498
427.1436
423.1529
428.1407
311.9992
422.1560
353.2661
451.9208
381.2972
472.1872
309.9999
127.3965
174.0247
255.9483
%
m/z

Compound 4c

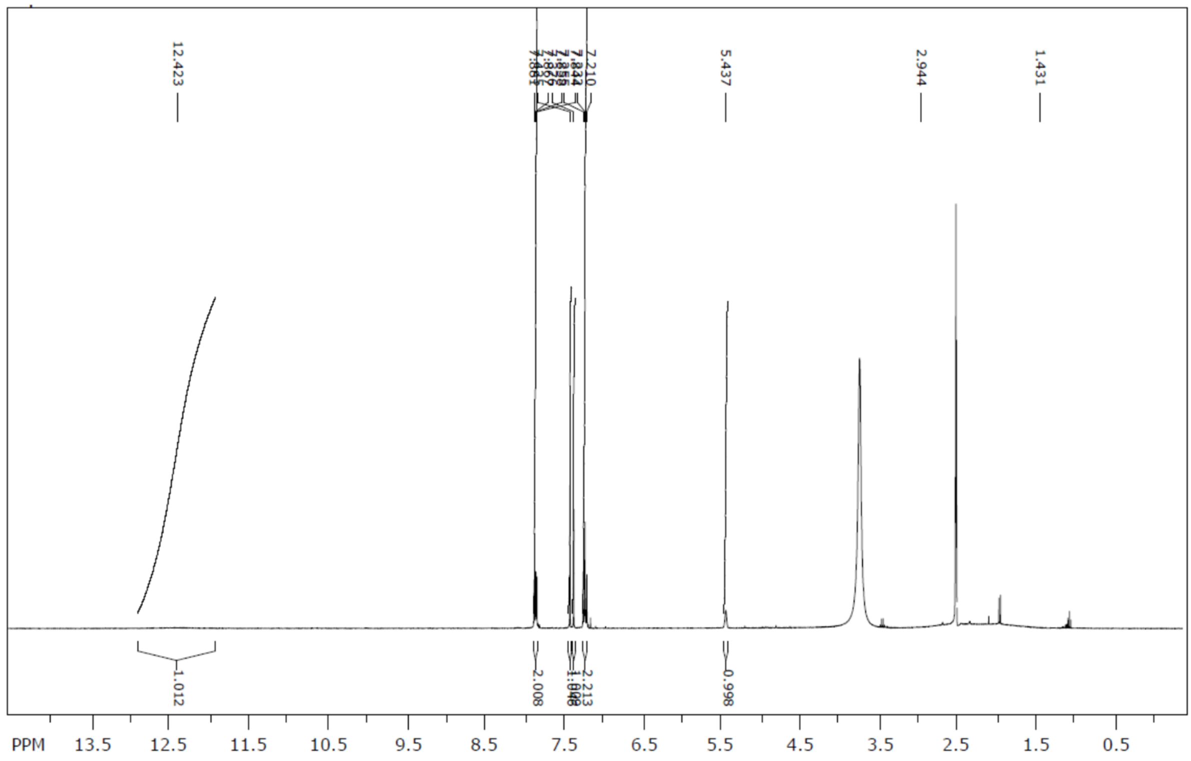
12.423
7.210
5.437
2.944
1.431
1.012
2.008
2.213
0.998
PPM

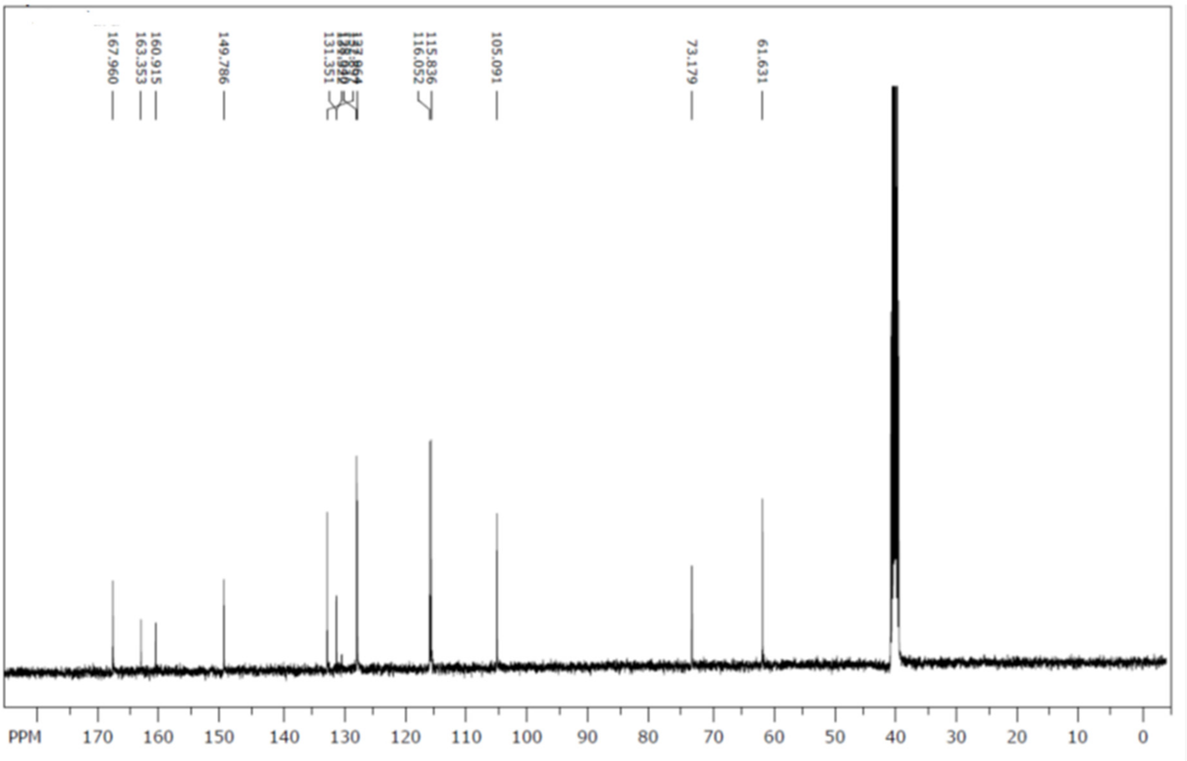
167.960
163.353
160.915
149.786
131.351
115.836
116.052
105.091
73.179
61.631
PPM 170 160 150 140 130 120 110 100 90 80 70 60 50 40 30 20 10 0

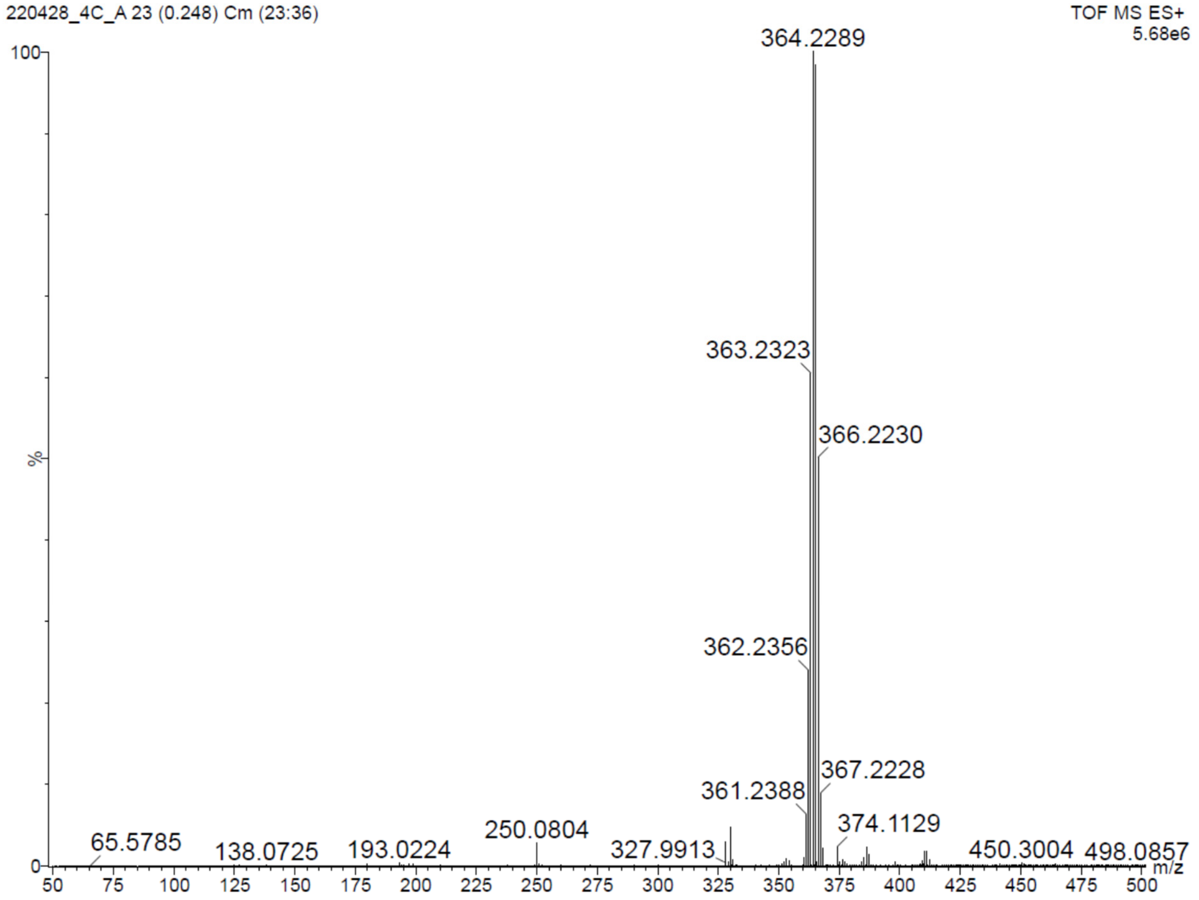
220428_4C_A 23 (0.248) Cm (23:36)
TOF MS ES+
5.68e6
364.2289
363.2323
366.2230
362.2356
367.2228
361.2388
374.1129
65.5785
138.0725
193.0224
250.0804
327.9913
450.3004
498.0857
%
m/z
100
0

Compound 4d

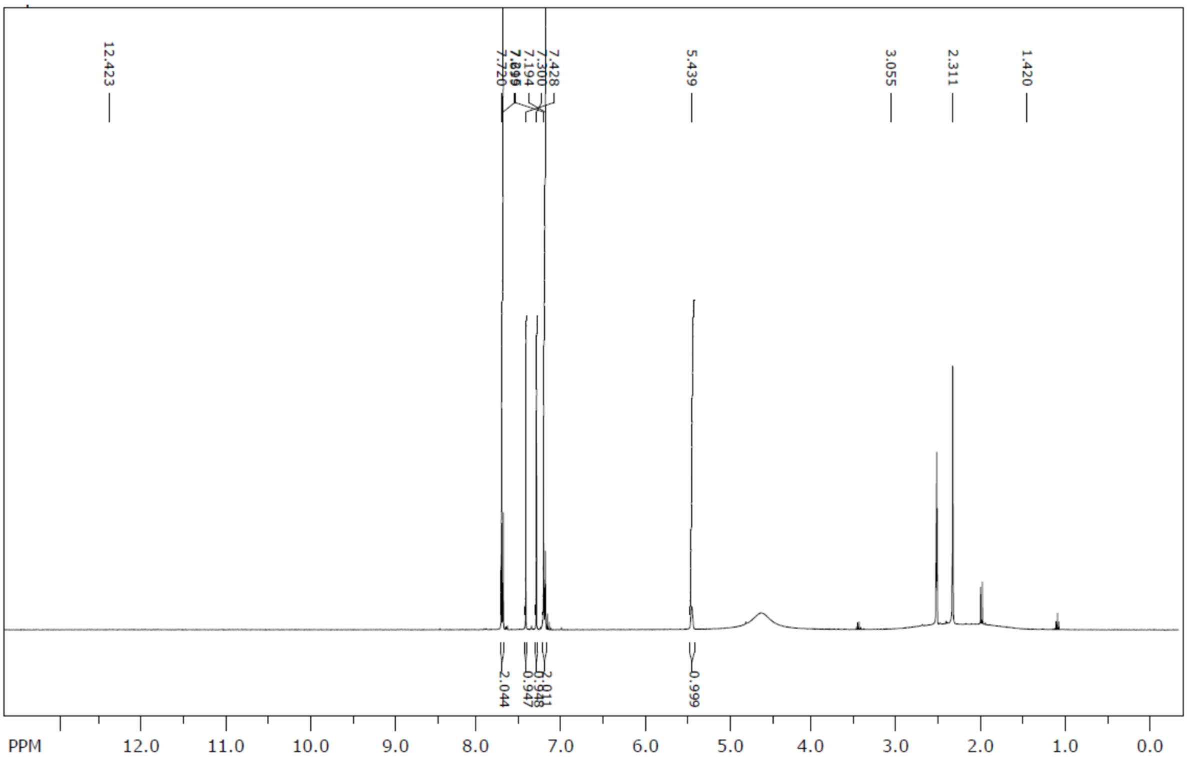

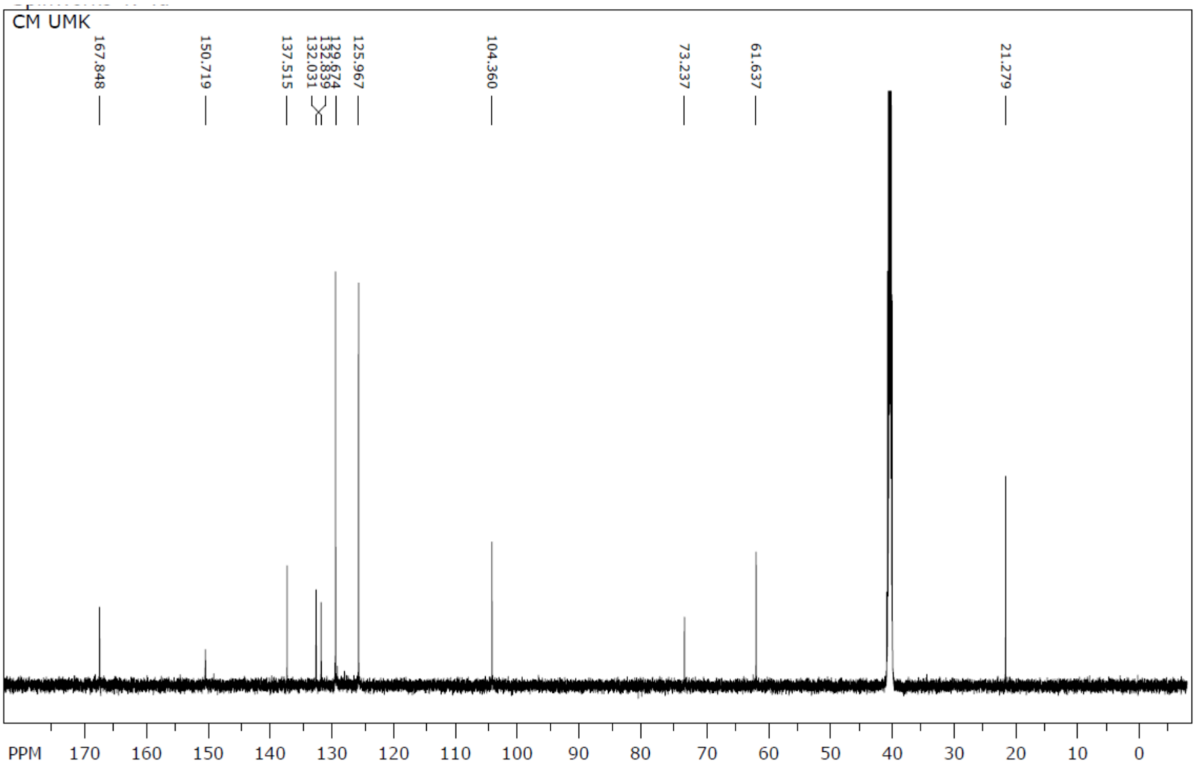

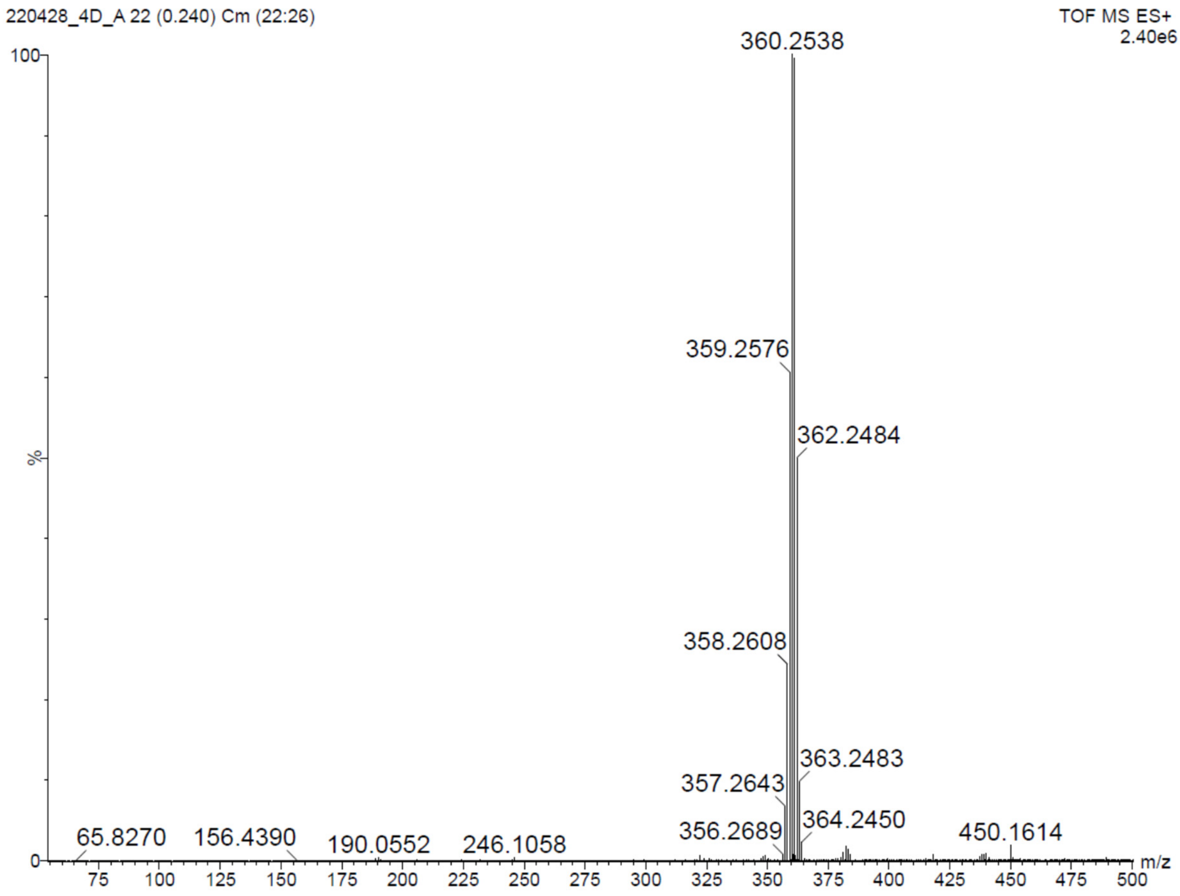
220428_4D_A 22 (0.240) Cm (22:26)
TOF MS ES+
2.40e6
360.2538
359.2576
362.2484
358.2608
363.2483
357.2643
364.2450
356.2689
450.1614
65.8270
156.4390
190.0552
246.1058
%
m/z

Compound 4e

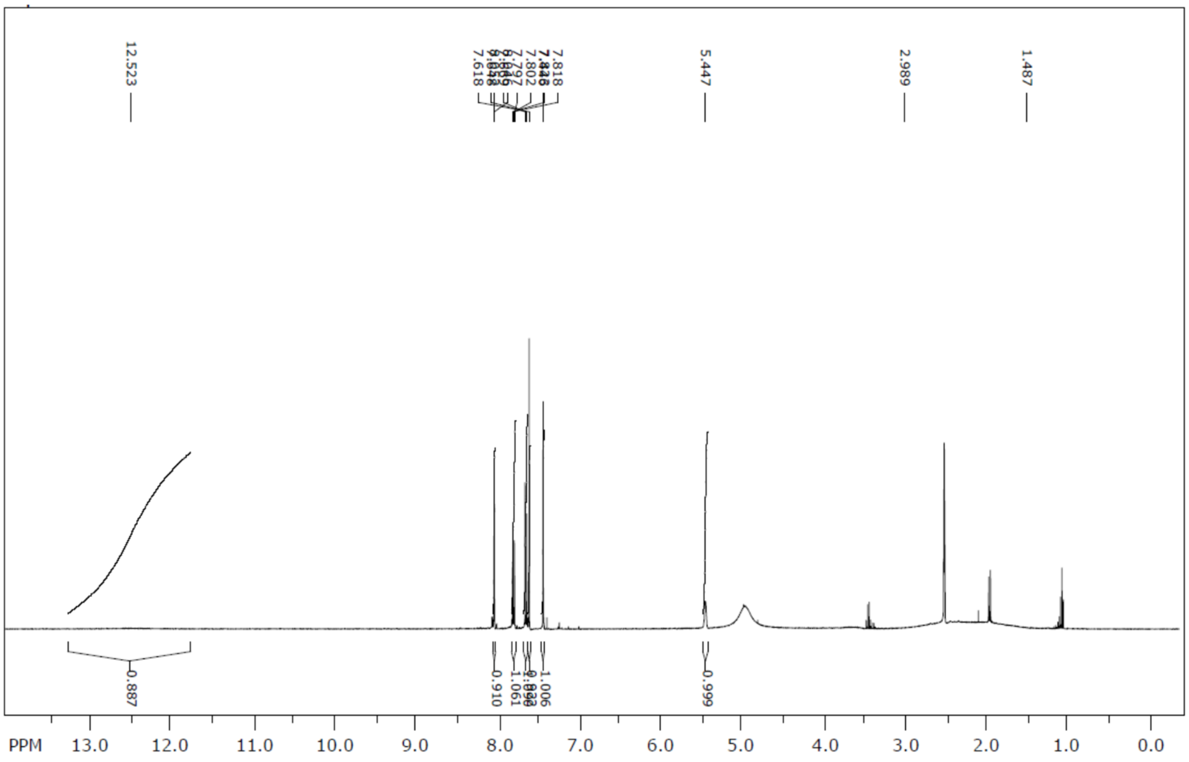
12.523
5.447
2.989
1.487
0.887
0.910
1.061
1.006
0.999
PPM

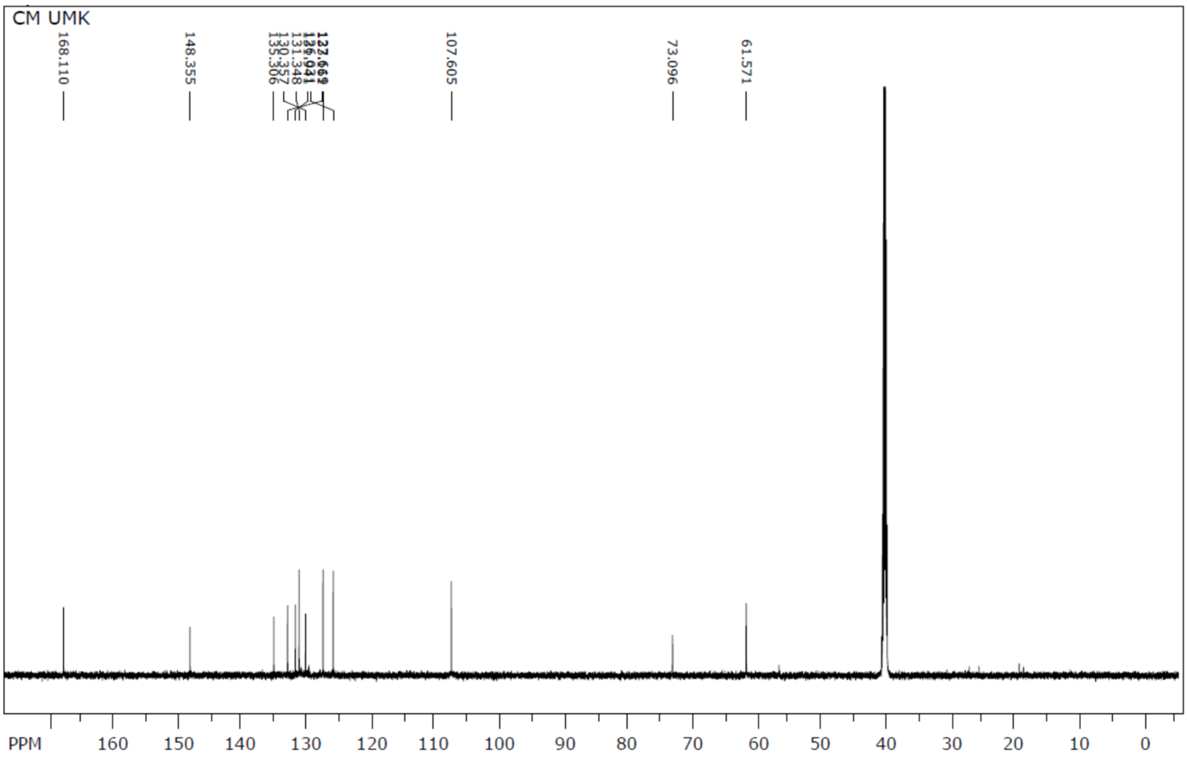
CM UMK
168.110
148.355
135.306
130.357
131.548
127.669
107.605
73.096
61.571
PPM
160
150
140
130
120
110
100
90
80
70
60
50
40
30
20
10
0

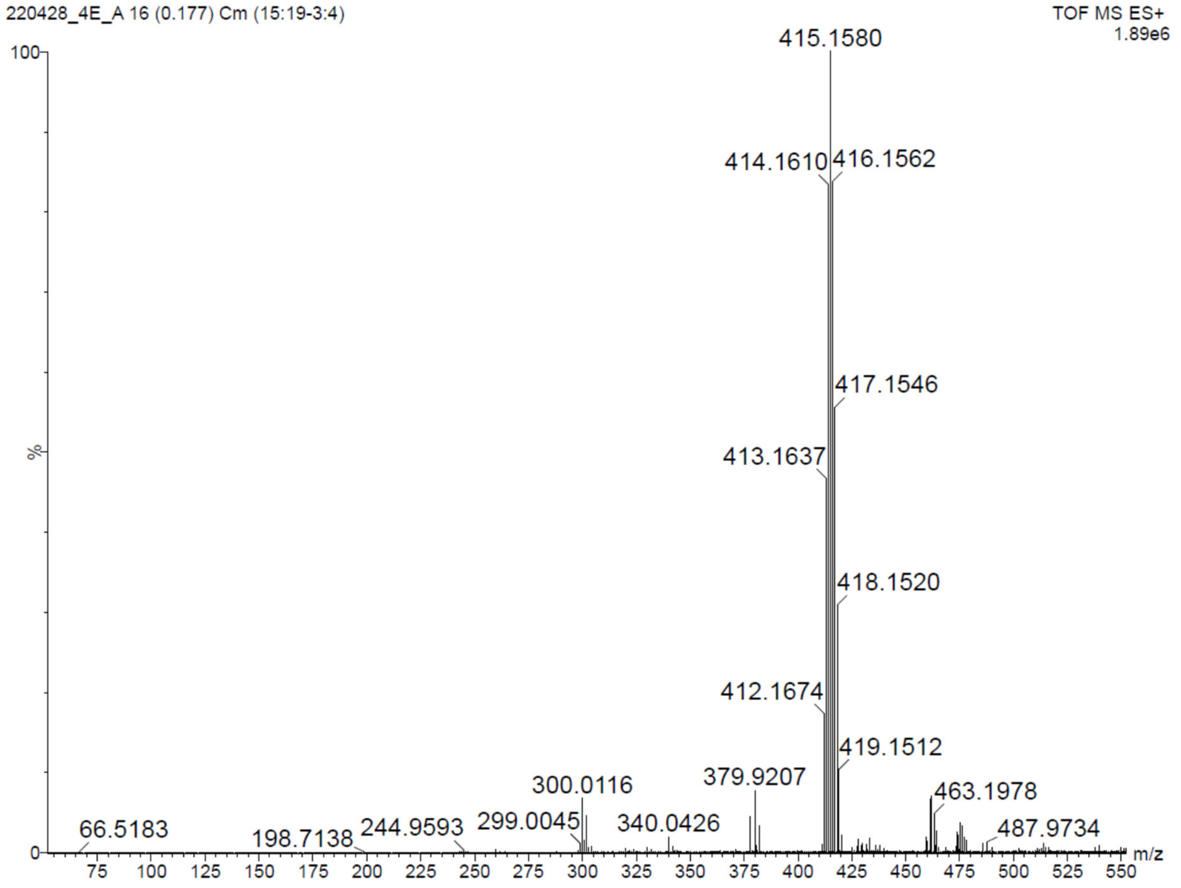
220428_4E_A 16 (0.177) Cm (15:19-3:4)
TOF MS ES+
1.89e6
415.1580
414.1610
416.1562
417.1546
413.1637
418.1520
412.1674
419.1512
300.0116
379.9207
463.1978
66.5183
198.7138
244.9593
299.0045
340.0426
487.9734
100
%
0
m/z

Compound 4f

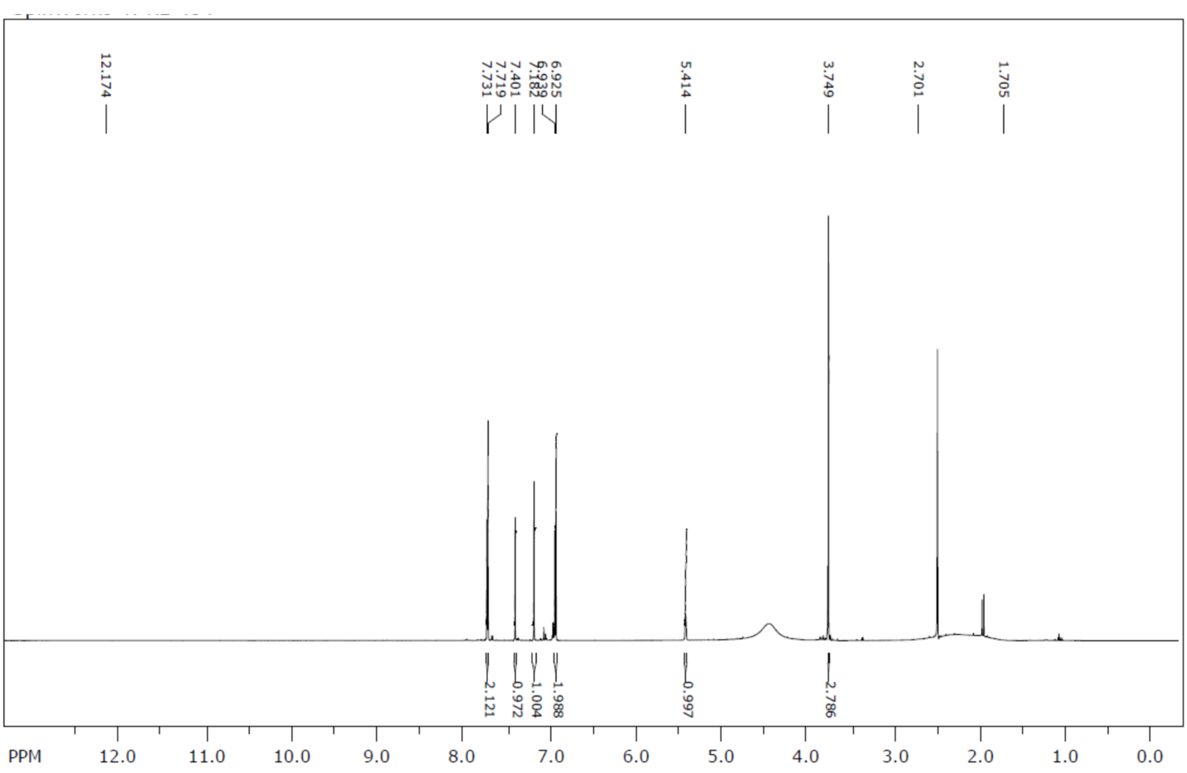
12.174
7.731
7.719
7.401
7.182
6.939
6.925
5.414
3.749
2.701
1.705
2.121
0.972
1.004
1.988
0.997
2.786
PPM 12.0 11.0 10.0 9.0 8.0 7.0 6.0 5.0 4.0 3.0 2.0 1.0 0.0

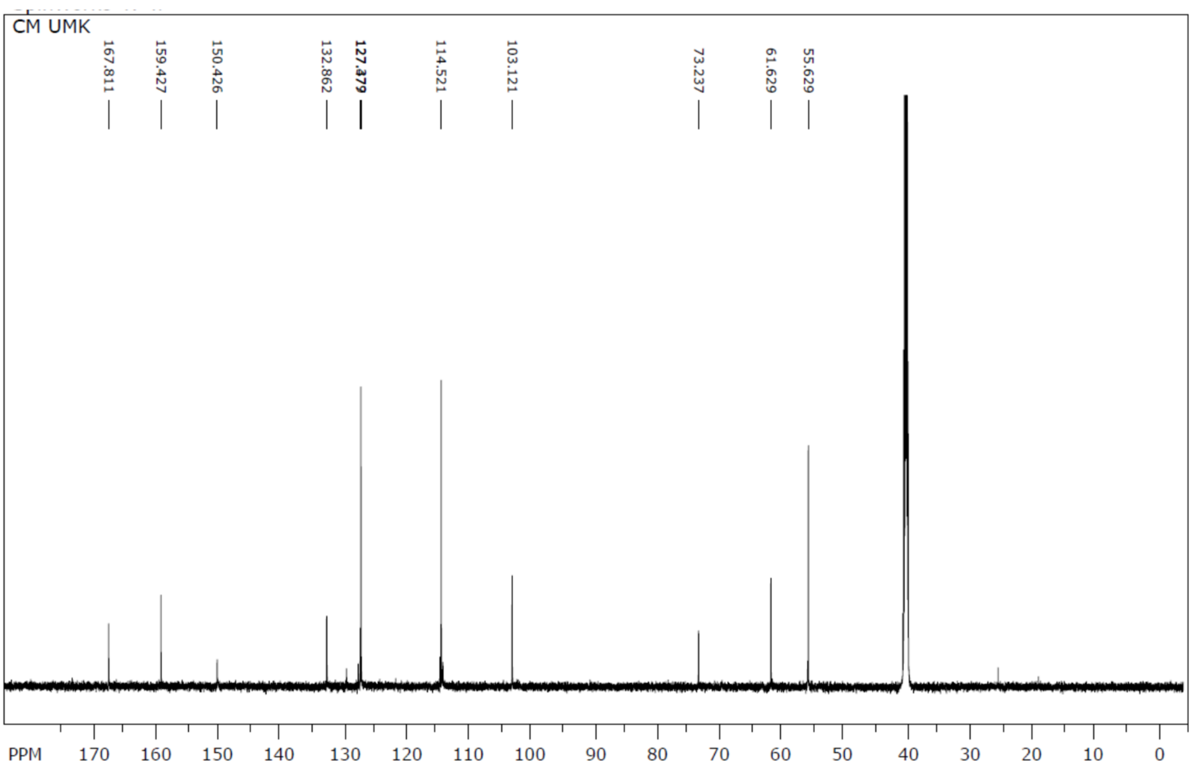
CM UMK
167.811
159.427
150.426
132.862
127.379
114.521
103.121
73.237
61.629
55.629
PPM 170 160 150 140 130 120 110 100 90 80 70 60 50 40 30 20 10 0

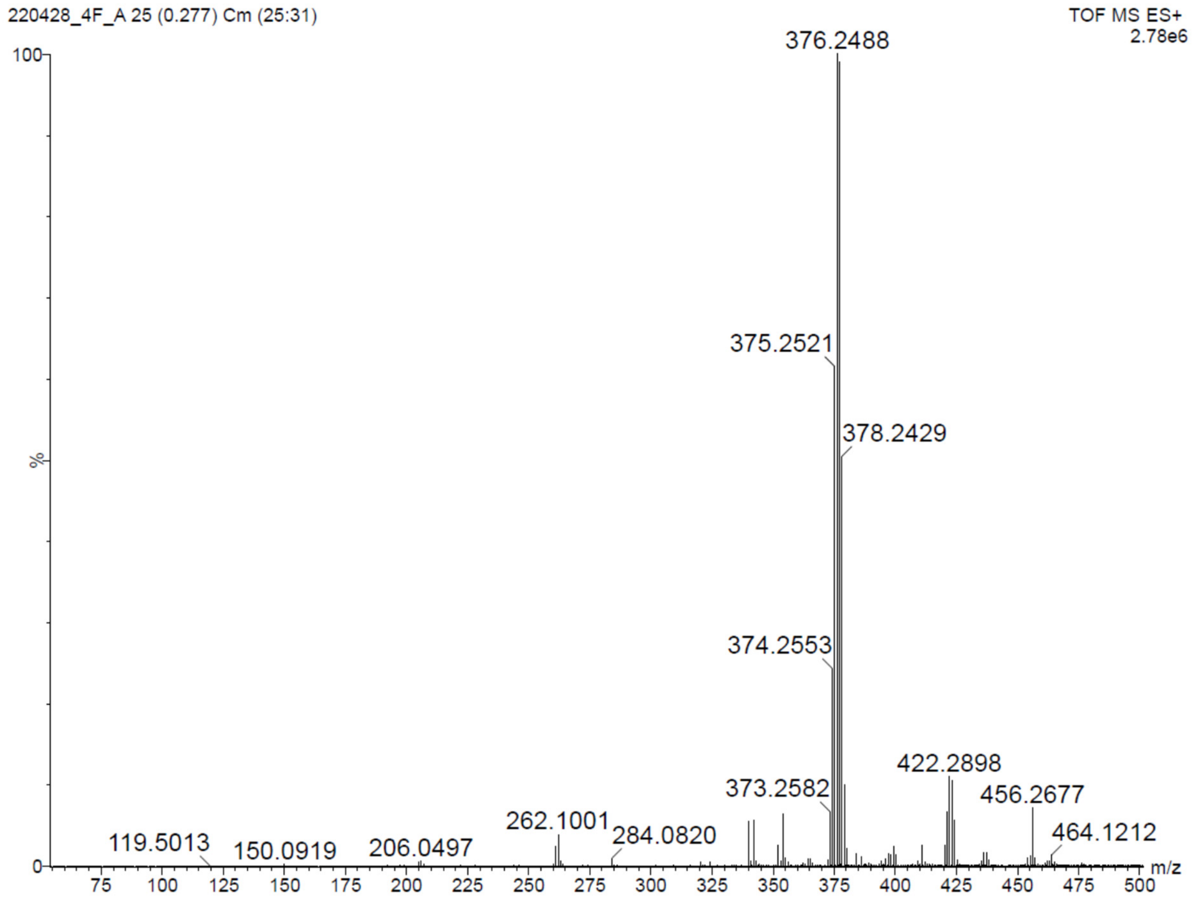

220428_4F_A 25 (0.277) Cm (25:31)
TOF MS ES+
2.78e6
376.2488
375.2521
378.2429
374.2553
373.2582
422.2898
456.2677
464.1212
262.1001
284.0820
119.5013
150.0919
206.0497
%
m/z

Compound 4g

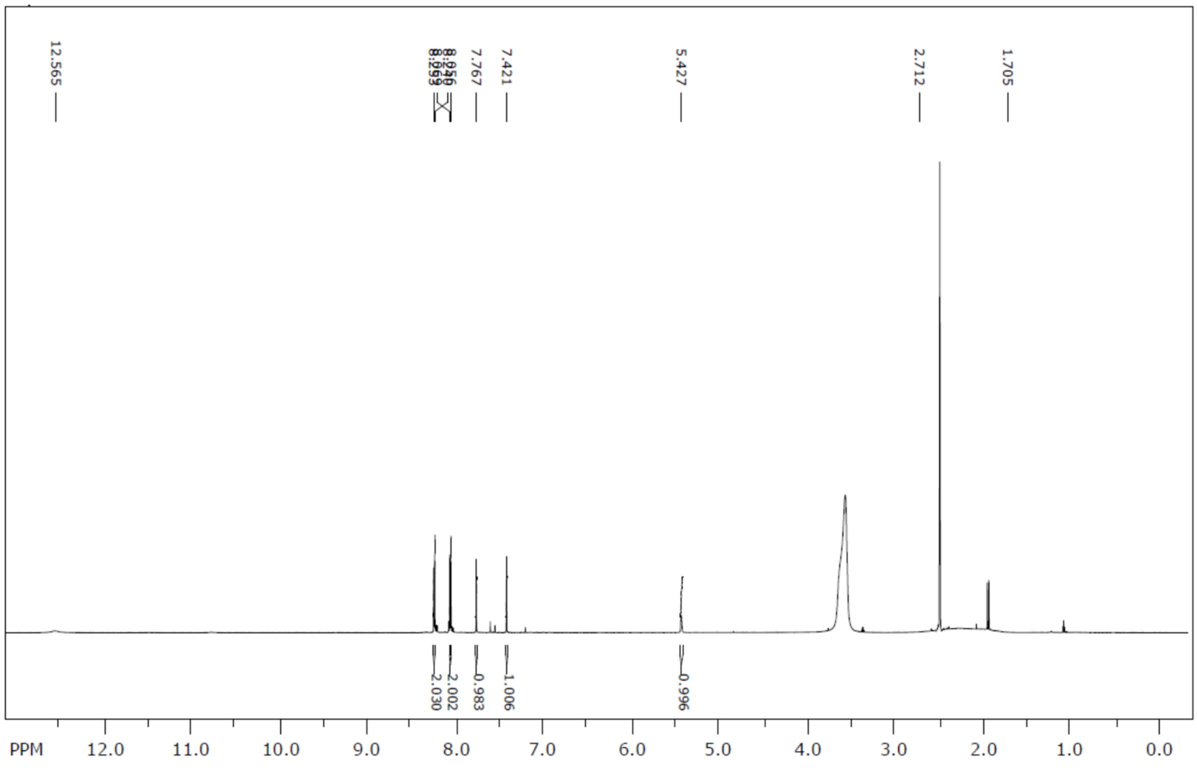

12.565
7.767
7.421
5.427
2.712
1.705
2.030
2.002
0.983
1.006
0.996
PPM

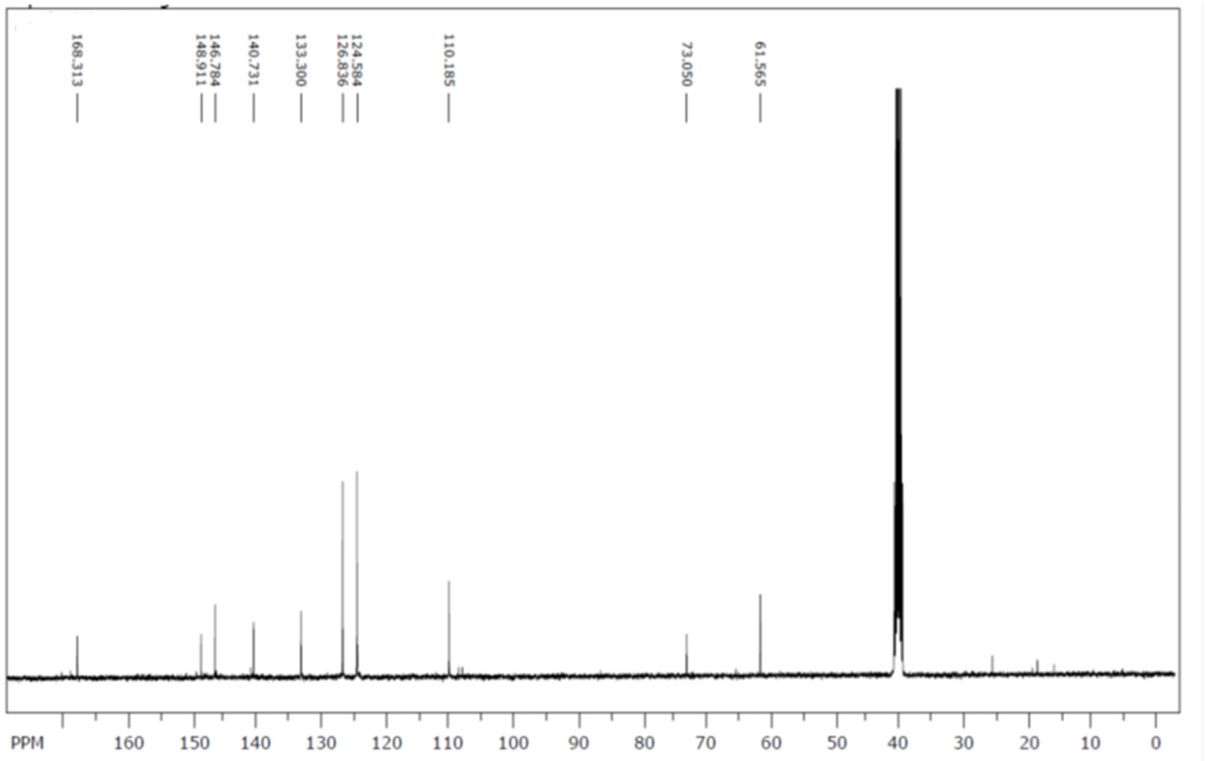

168.313
148.911
146.784
140.731
133.300
126.836
124.584
110.185
73.050
61.565
PPM
160
150
140
130
120
110
100
90
80
70
60
50
40
30
20
10
0

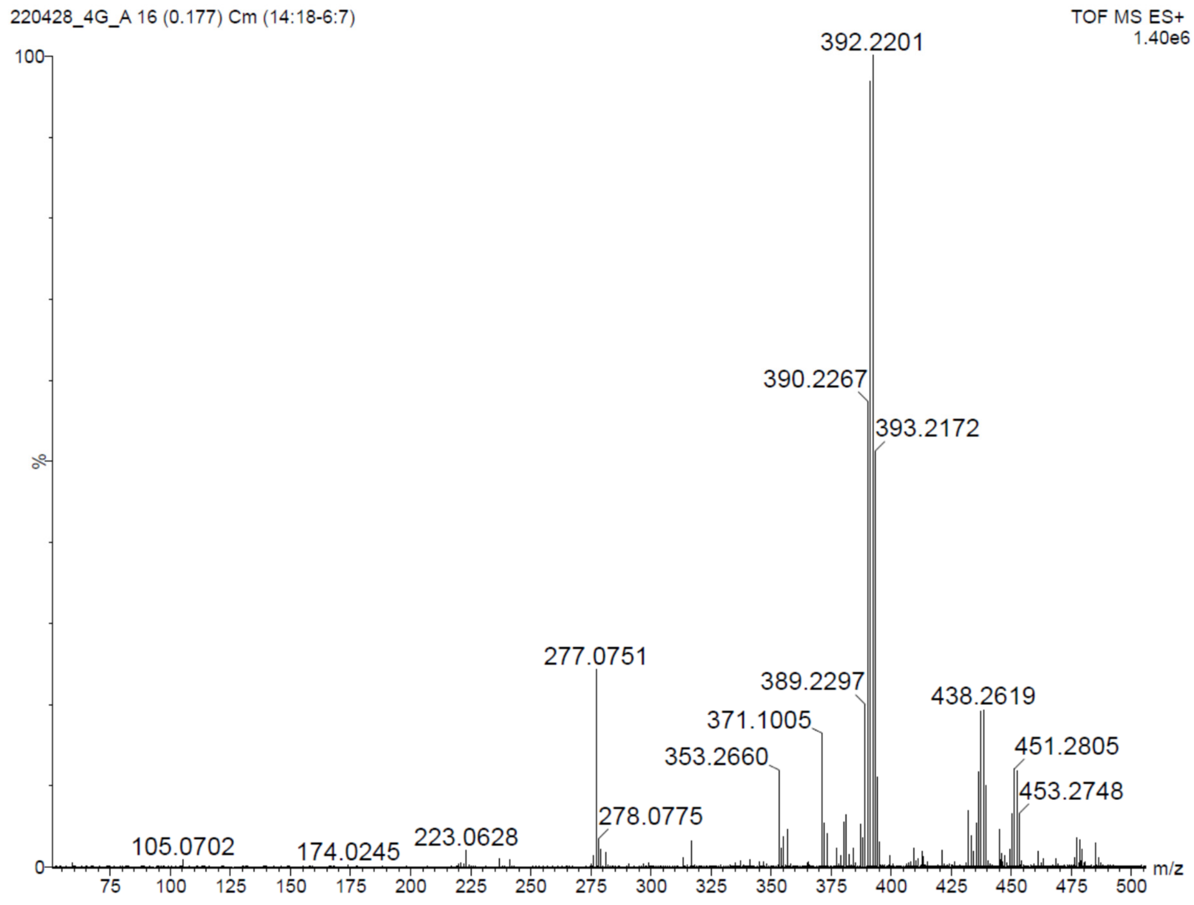

220428_4G_A 16 (0.177) Cm (14:18-6:7)
TOF MS ES+
1.40e6
392.2201
390.2267
393.2172
277.0751
389.2297
438.2619
371.1005
451.2805
353.2660
453.2748
278.0775
223.0628
105.0702
174.0245
100
%
0
m/z

Compound 4h

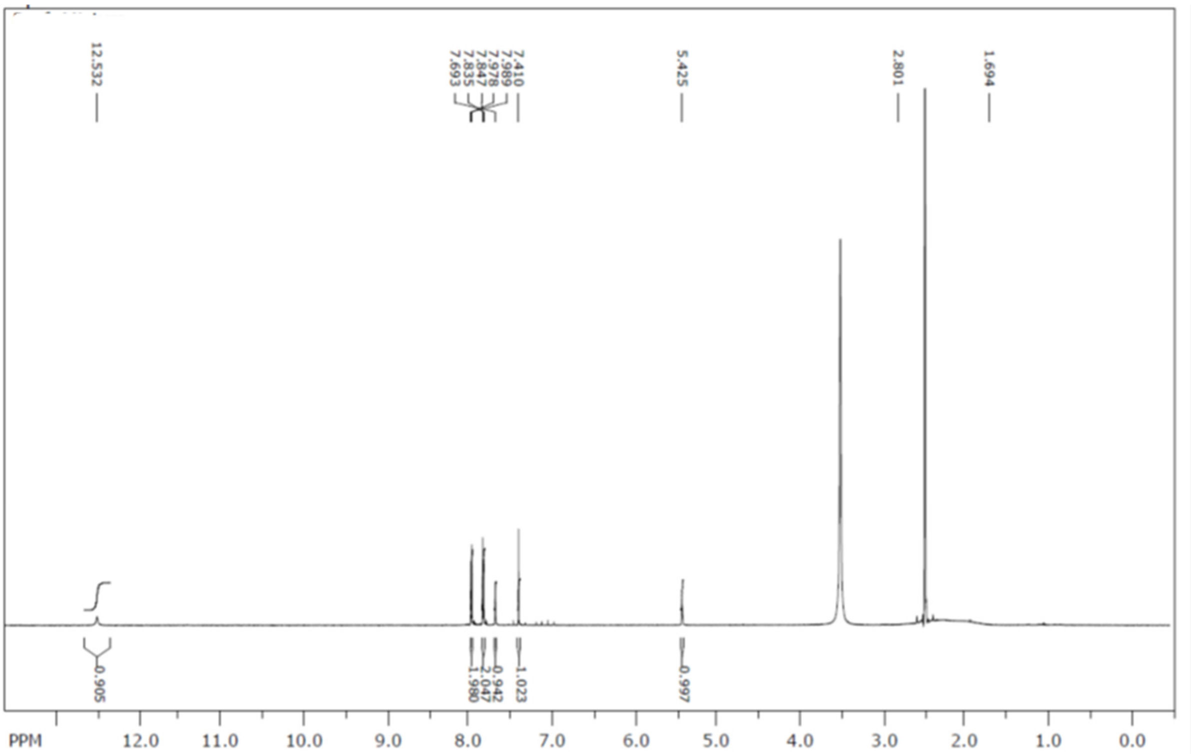
12.532
7.410
7.989
7.978
7.847
7.835
7.693
5.425
2.801
1.694
0.905
1.980
2.047
0.942
1.023
0.997
PPM
12.0
11.0
10.0
9.0
8.0
7.0
6.0
5.0
4.0
3.0
2.0
1.0
0.0

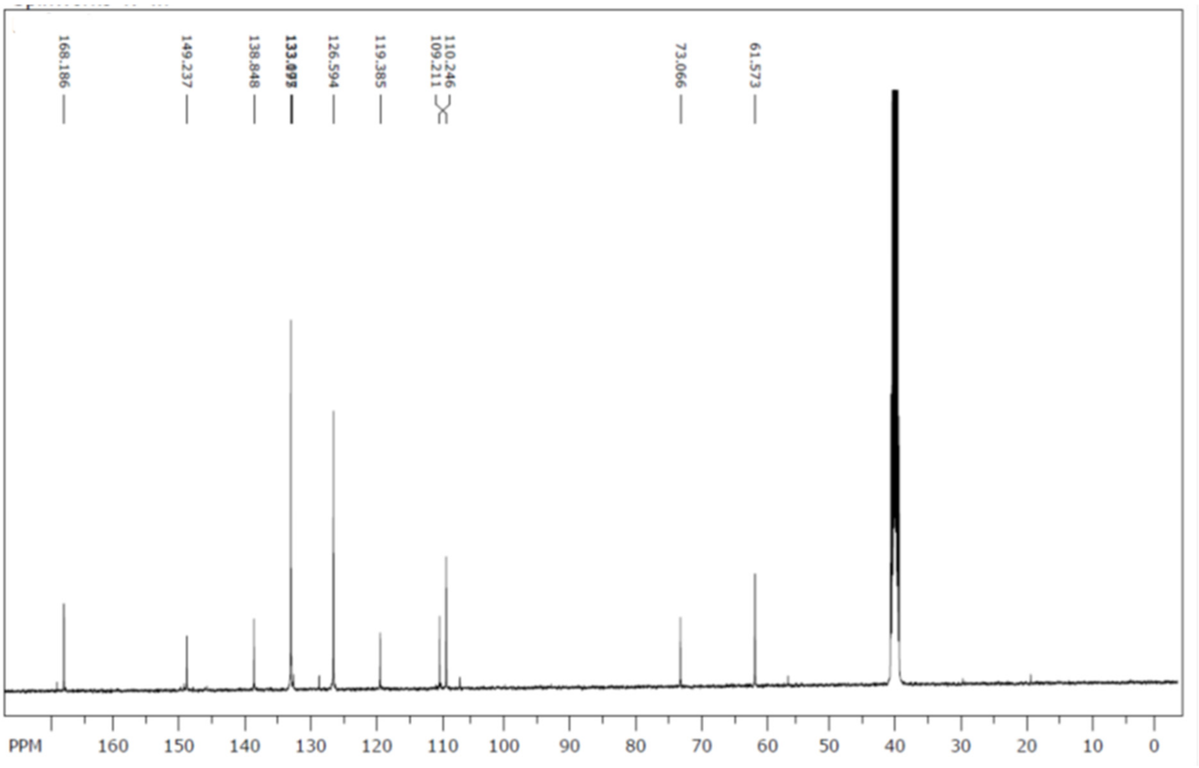
168.186
149.237
138.848
126.594
119.385
110.246
109.211
73.066
61.573
PPM
160
150
140
130
120
110
100
90
80
70
60
50
40
30
20
10
0

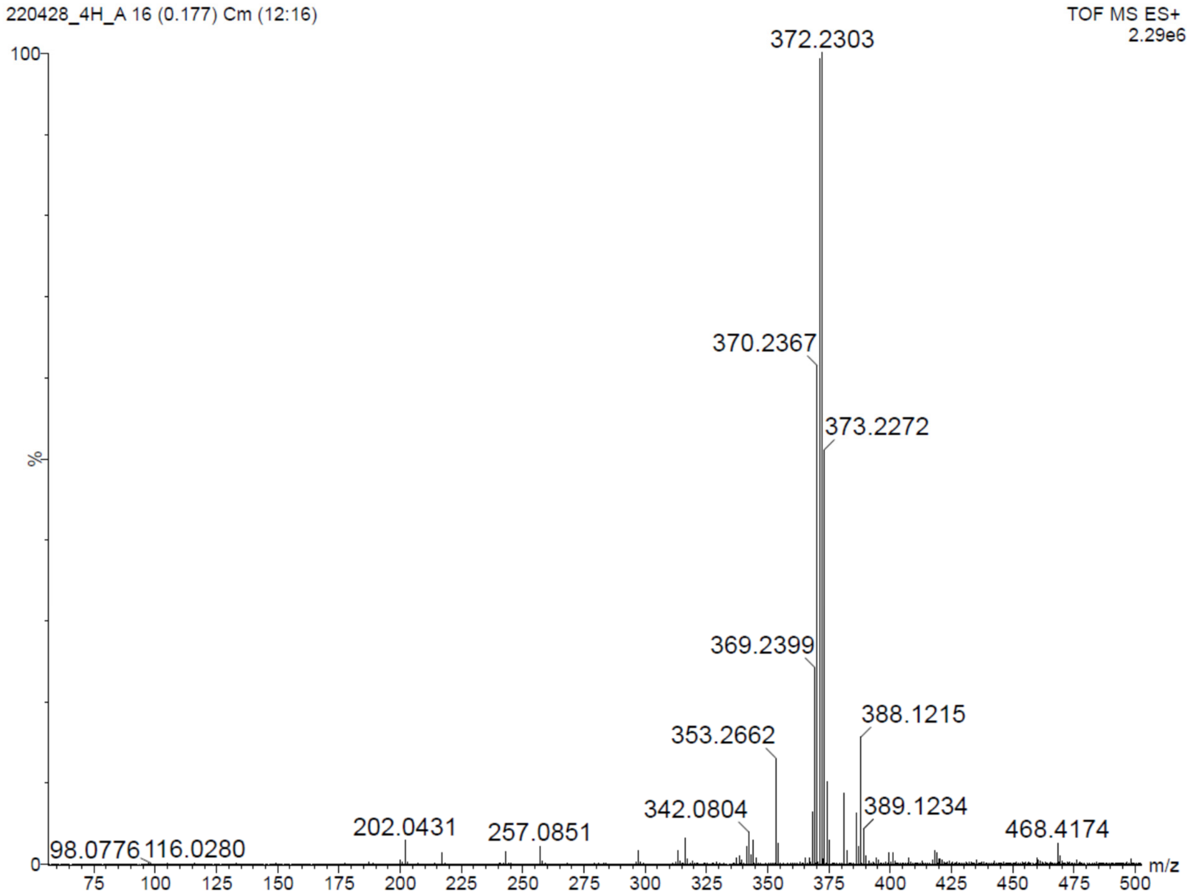
220428_4H_A 16 (0.177) Cm (12:16)
TOF MS ES+
2.29e6
100
%
0
372.2303
370.2367
373.2272
369.2399
388.1215
353.2662
342.0804
389.1234
202.0431
257.0851
468.4174
98.0776
116.0280
75
100
125
150
175
200
225
250
275
300
325
350
375
400
425
450
475
500
m/z

Compound 4i

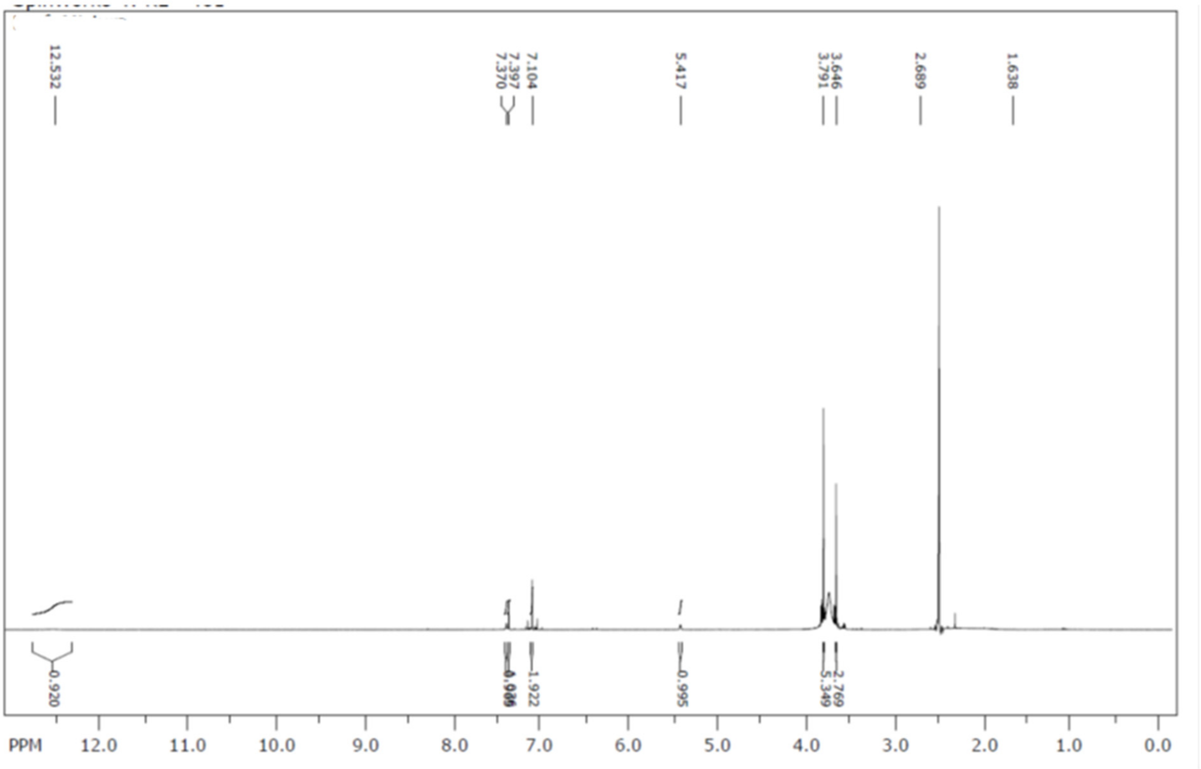
12.532
7.397
7.370
7.104
5.417
3.791
3.646
2.689
1.638
0.920
1.922
0.995
5.349
2.769
PPM
12.0
11.0
10.0
9.0
8.0
7.0
6.0
5.0
4.0
3.0
2.0
1.0
0.0

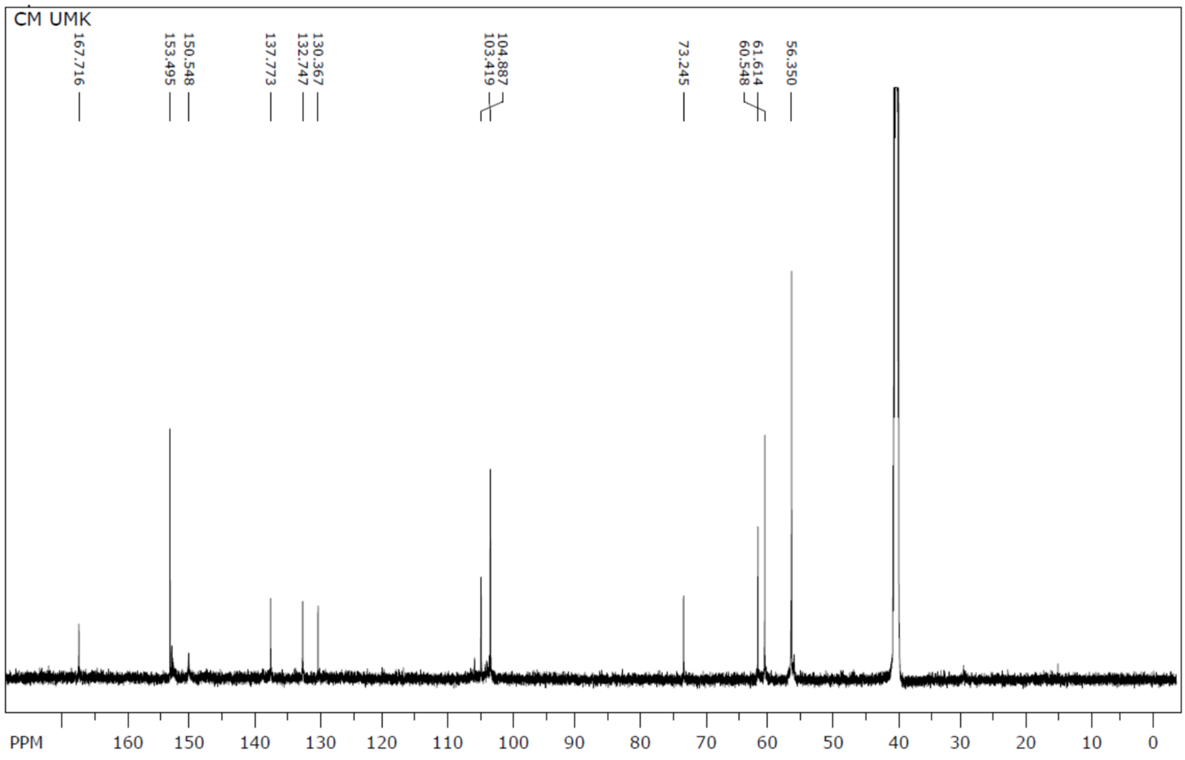
CM UMK
167.716
153.495
150.548
137.773
132.747
130.367
104.887
103.419
73.245
61.614
60.548
56.350
PPM
160 150 140 130 120 110 100 90 80 70 60 50 40 30 20 10 0

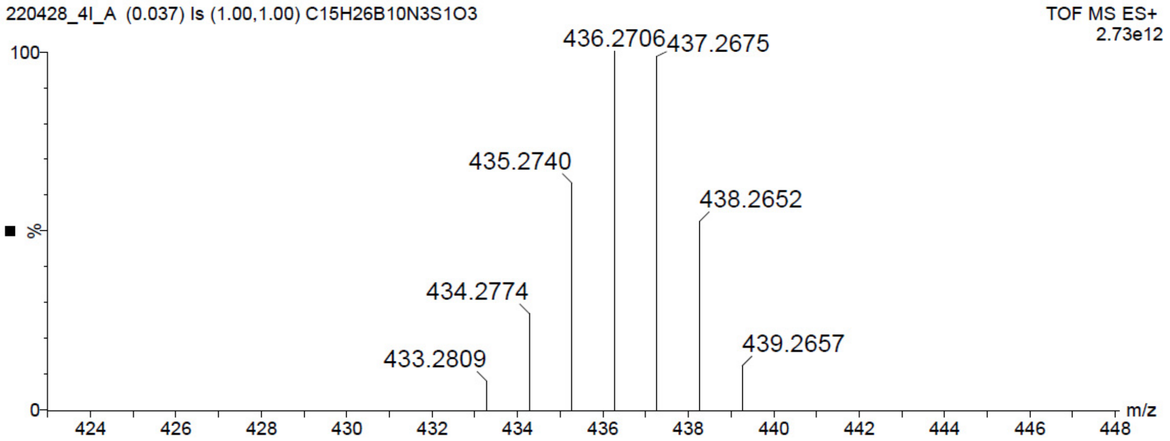
220428_4I_A (0.037) Is (1.00,1.00) C15H26B10N3S1O3
TOF MS ES+
2.73e12
436.2706
437.2675
435.2740
438.2652
434.2774
433.2809
439.2657
%
m/z
424 426 428 430 432 434 436 438 440 442 444 446 448

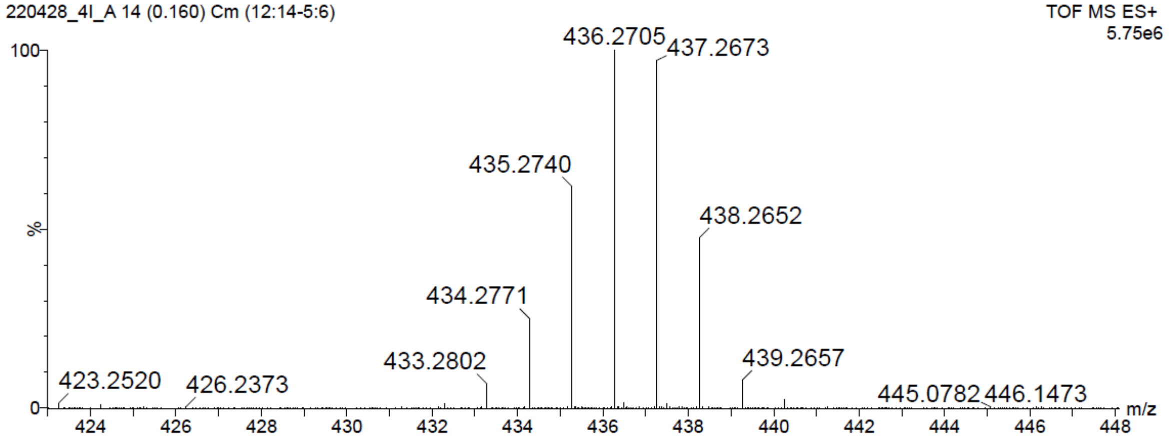
220428_4I_A 14 (0.160) Cm (12:14-5:6)
TOF MS ES+
5.75e6
436.2705
437.2673
435.2740
438.2652
434.2771
433.2802
439.2657
423.2520
426.2373
445.0782
446.1473
%
m/z
424 426 428 430 432 434 436 438 440 442 444 446 448

Compound 4j

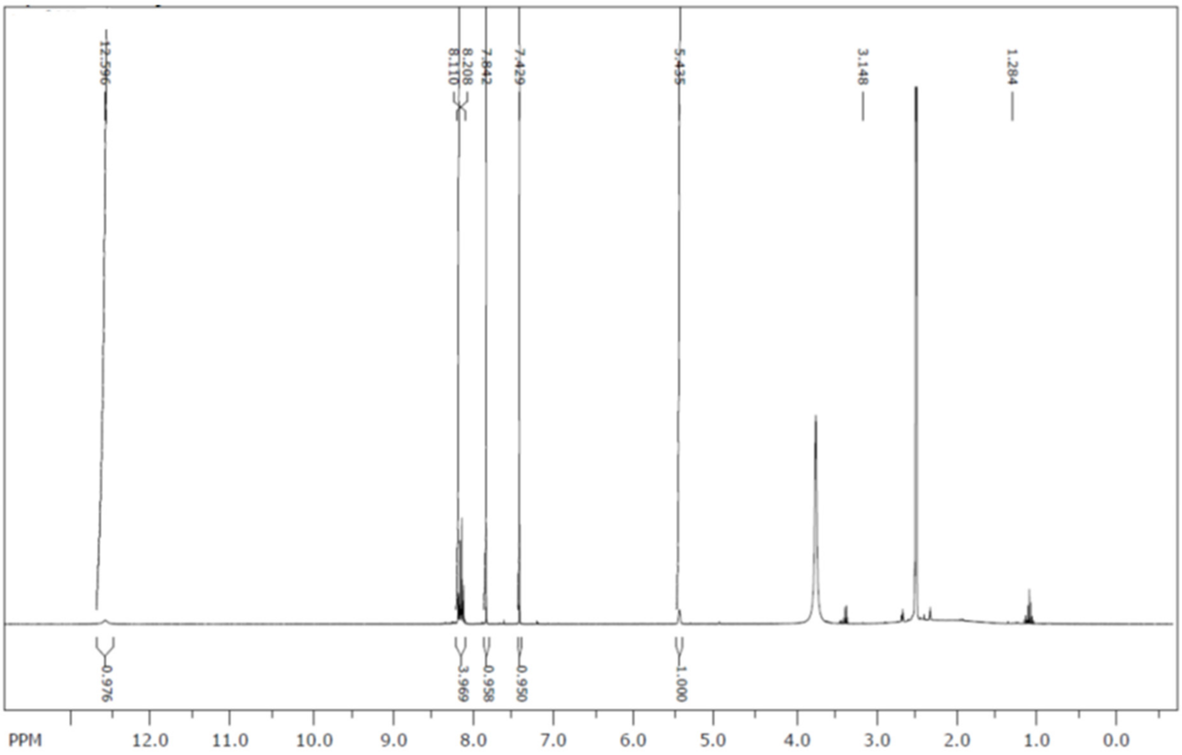
12.596
8.208
8.110
7.842
7.429
5.435
3.148
1.284
0.976
3.969
0.958
0.950
1.000
PPM
12.0
11.0
10.0
9.0
8.0
7.0
6.0
5.0
4.0
3.0
2.0
1.0
0.0

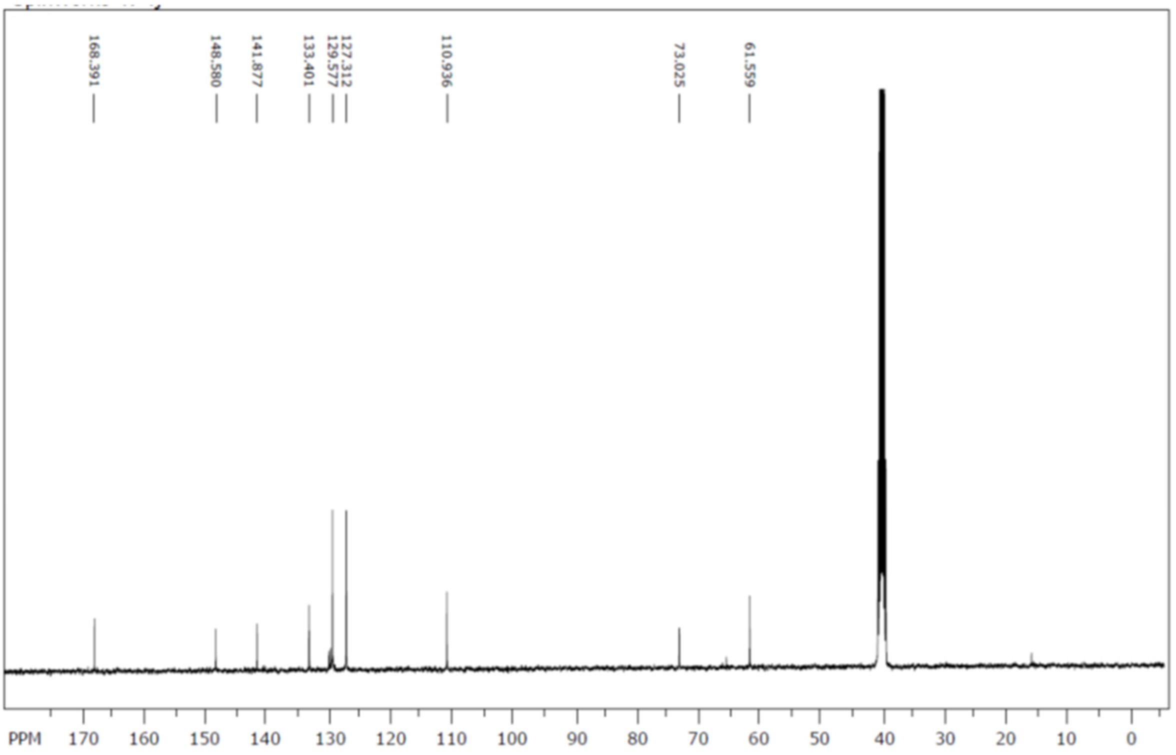
168.391
148.580
141.877
133.401
129.577
127.312
110.936
73.025
61.559
PPM
170
160
150
140
130
120
110
100
90
80
70
60
50
40
30
20
10
0

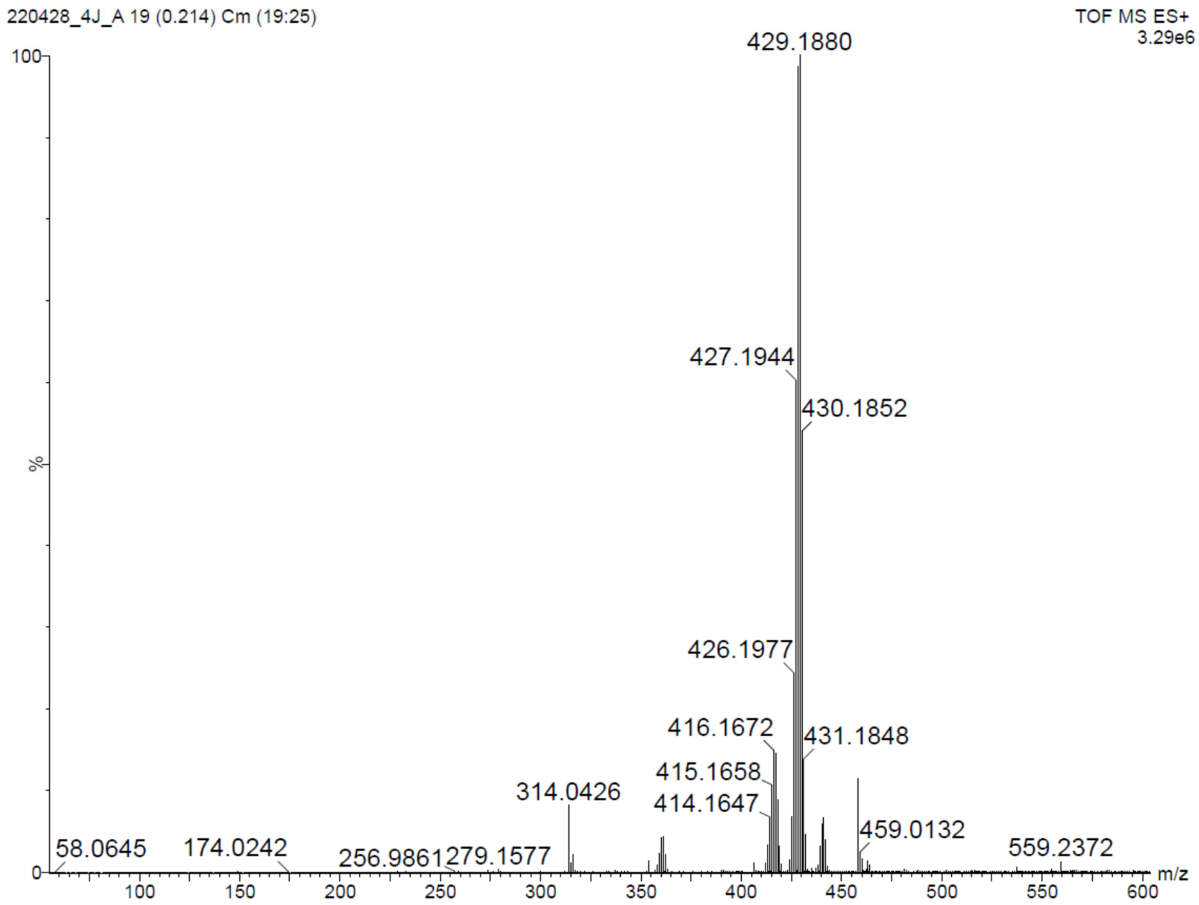

220428_4J_A 19 (0.214) Cm (19:25)
TOF MS ES+
3.29e6
429.1880
427.1944
430.1852
426.1977
416.1672
431.1848
415.1658
414.1647
314.0426
459.0132
58.0645
174.0242
256.9861
279.1577
559.2372
m/z
%

Compound 4k

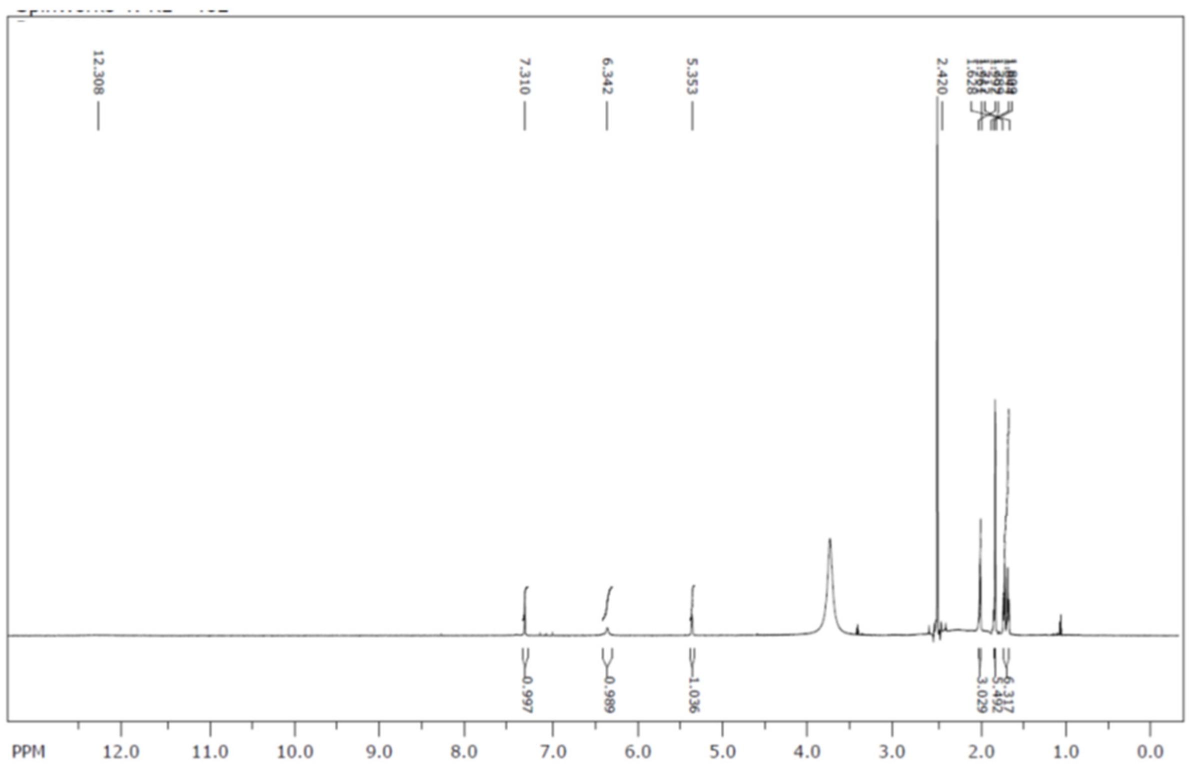

12.308
7.310
6.342
5.353
2.420
0.997
0.989
1.036
3.029
3.402
6.317
PPM

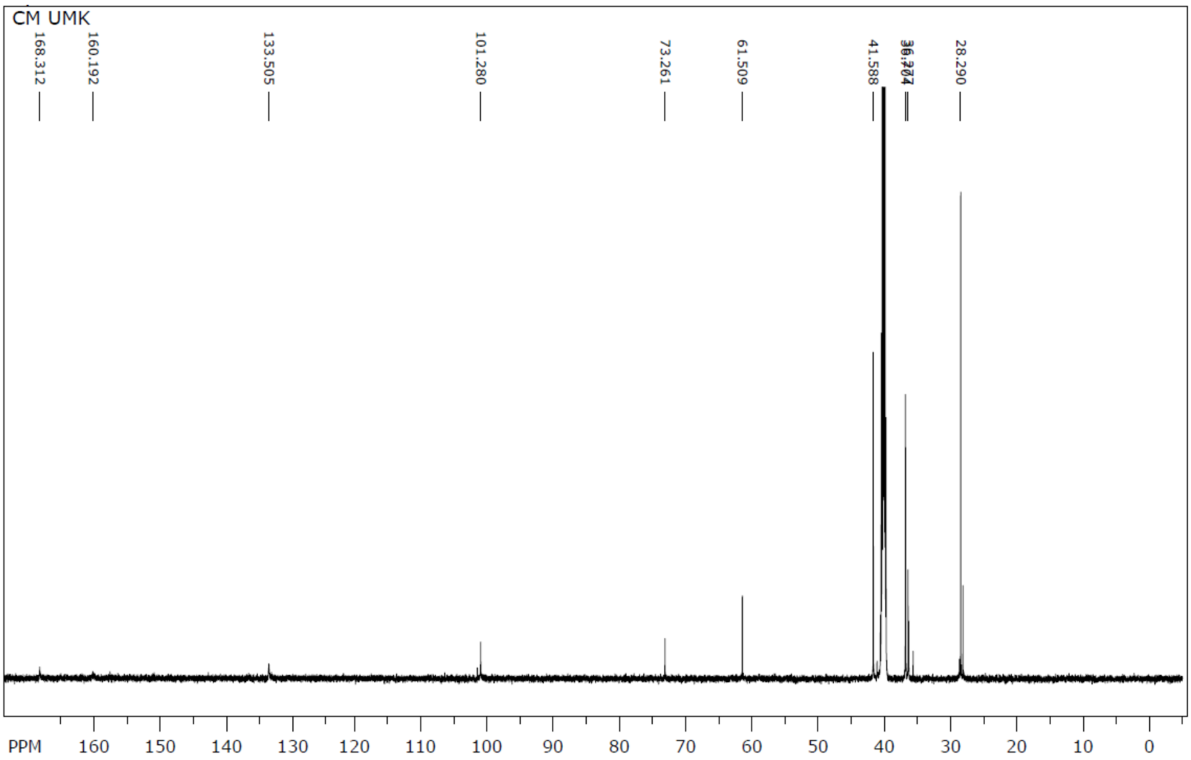
CM UMK
168.312
160.192
133.505
101.280
73.261
61.509
41.588
28.290
PPM
160 150 140 130 120 110 100 90 80 70 60 50 40 30 20 10 0

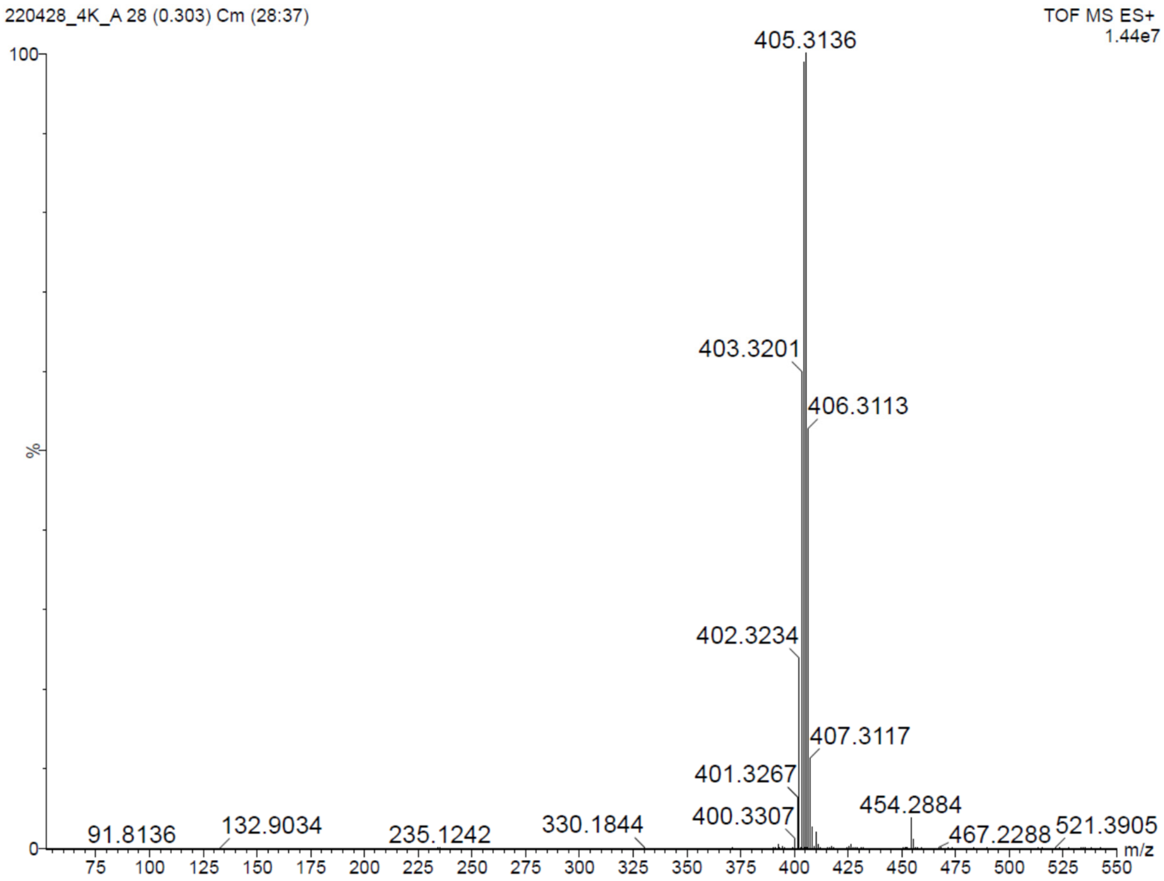
220428_4K_A 28 (0.303) Cm (28:37)
TOF MS ES+
1.44e7
405.3136
403.3201
406.3113
402.3234
407.3117
401.3267
454.2884
400.3307
91.8136
132.9034
235.1242
330.1844
467.2288
521.3905
100
%
0
m/z
75 100 125 150 175 200 225 250 275 300 325 350 375 400 425 450 475 500 525 550

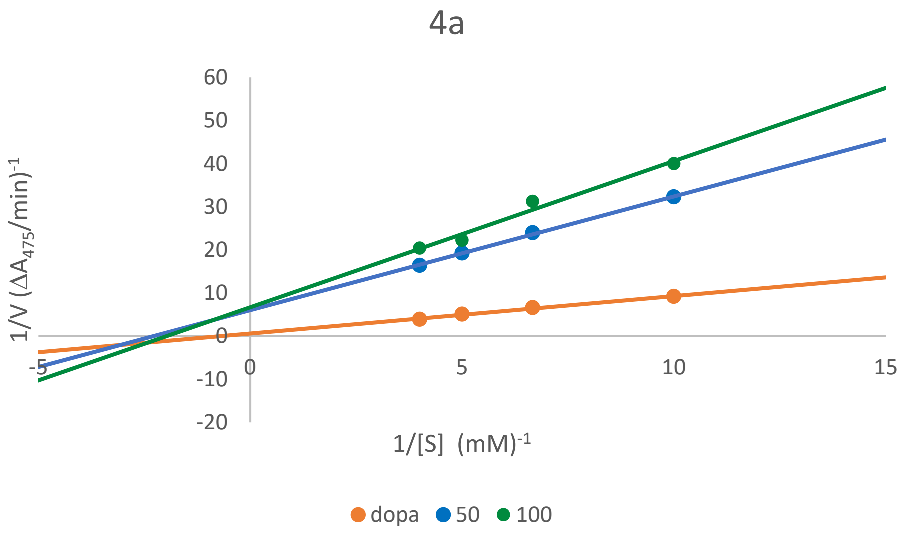
4a
60
50
40
30
20
10
0
-10
-20
-5
0
5
10
15
1/V (ΔA475/min)-1
1/[S] (mM)-1
dopa
50
100

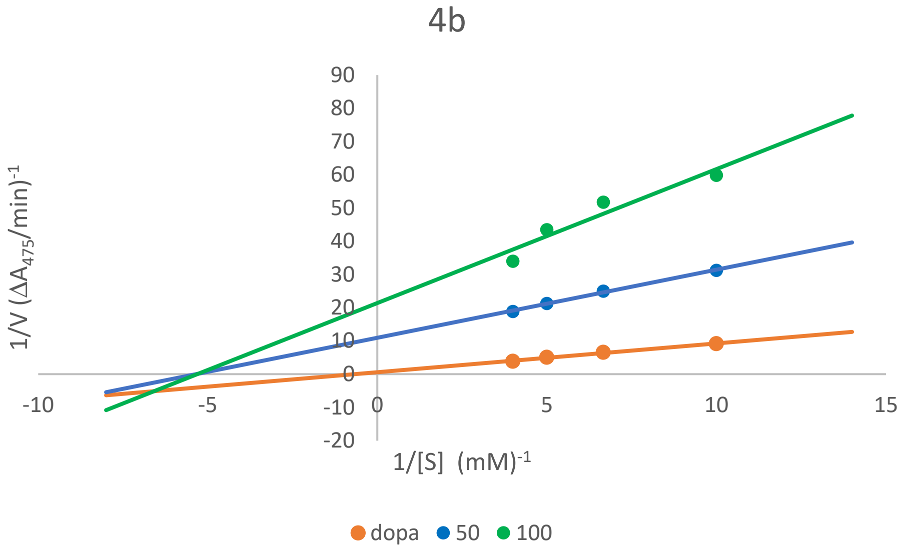
4b
90
80
70
60
50
40
30
20
10
0
-10
-20
-10
-5
0
5
10
15
1/V (ΔA475/min)-1
1/[S] (mM)-1
dopa
50
100

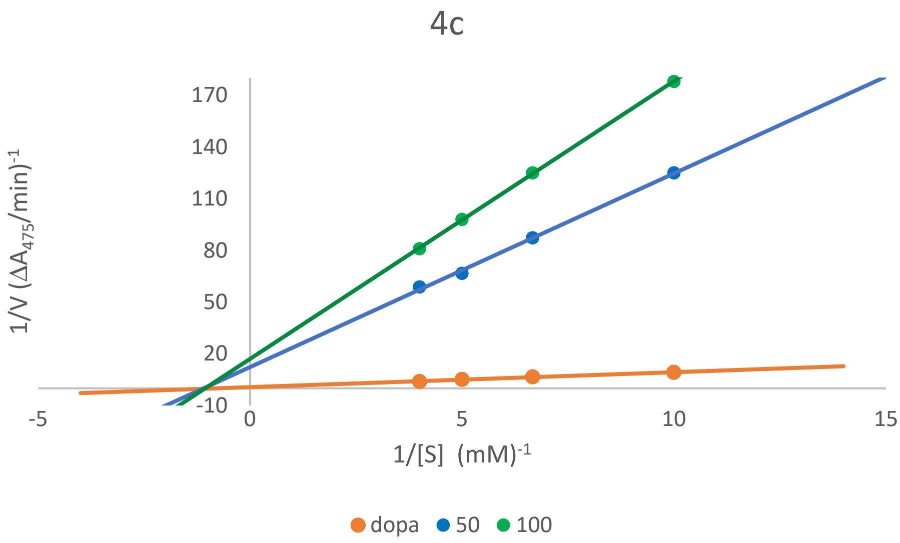
4c
170
140
110
80
50
20
-10
-5
0
5
10
15
1/V (ΔA475/min)-1
1/[S] (mM)-1
dopa
50
100

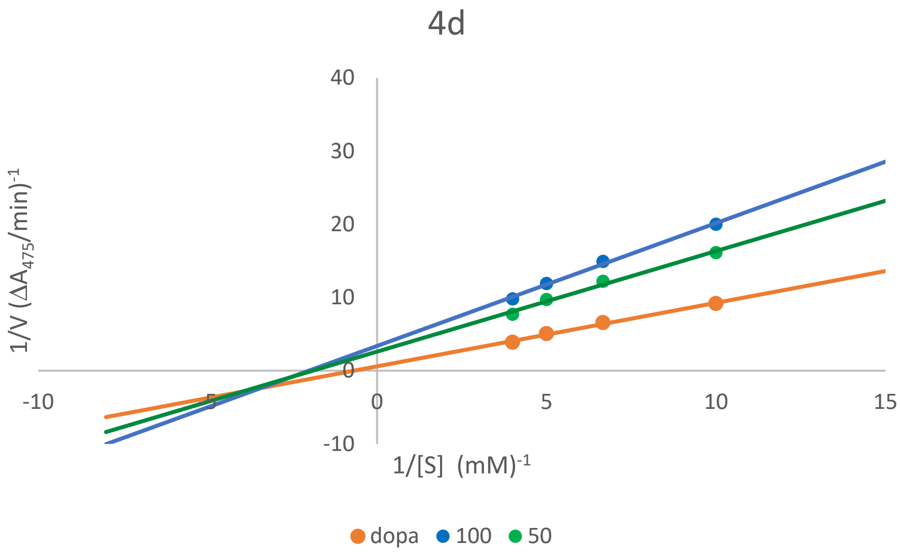
4d
40
30
20
10
0
-10
-10
-5
0
5
10
15
1/V (ΔA475/min)-1
1/[S] (mM)-1
dopa
100
50

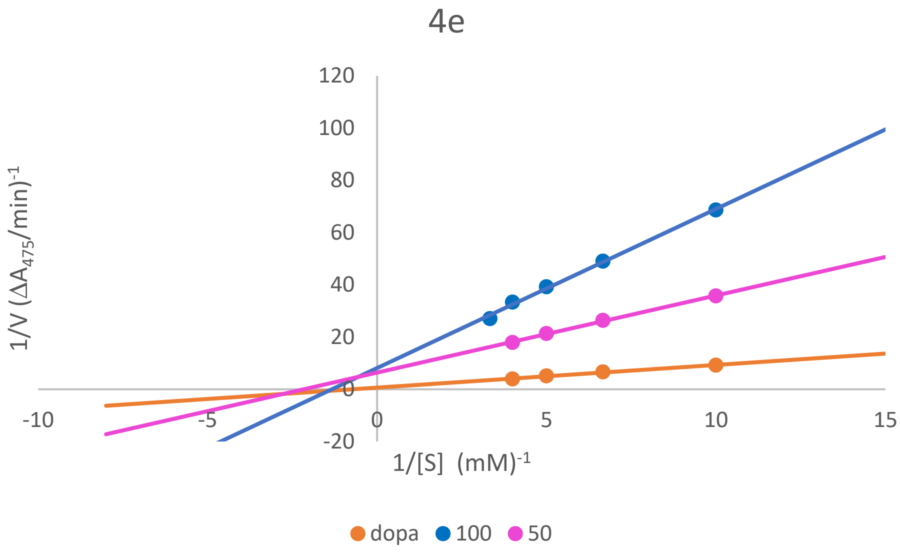
4e
120
100
80
60
40
20
0
-20
-10
-5
0
5
10
15
1/V (ΔA475/min)-1
1/[S] (mM)-1
dopa
100
50

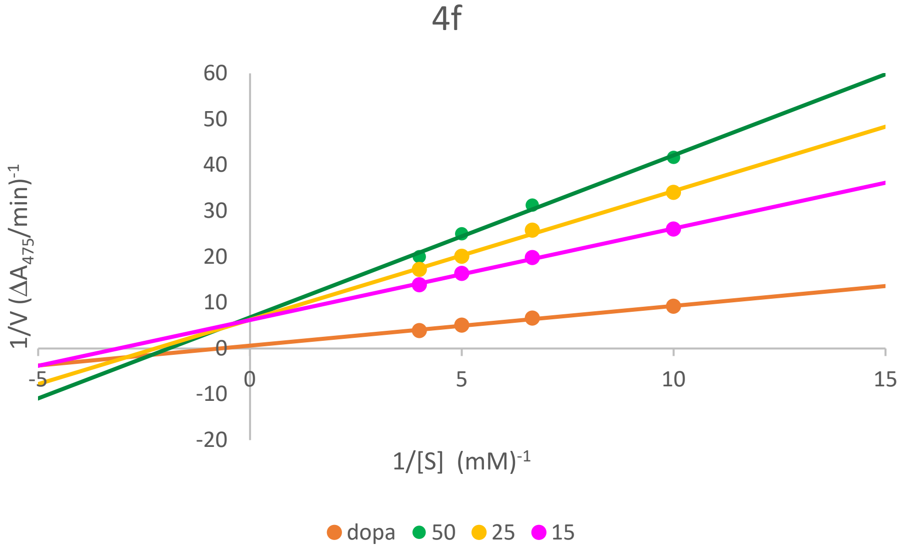
4f
60
50
40
30
20
10
0
-10
-20
-5
0
5
10
15
1/V (ΔA475/min)-1
1/[S] (mM)-1
dopa
50
25
15

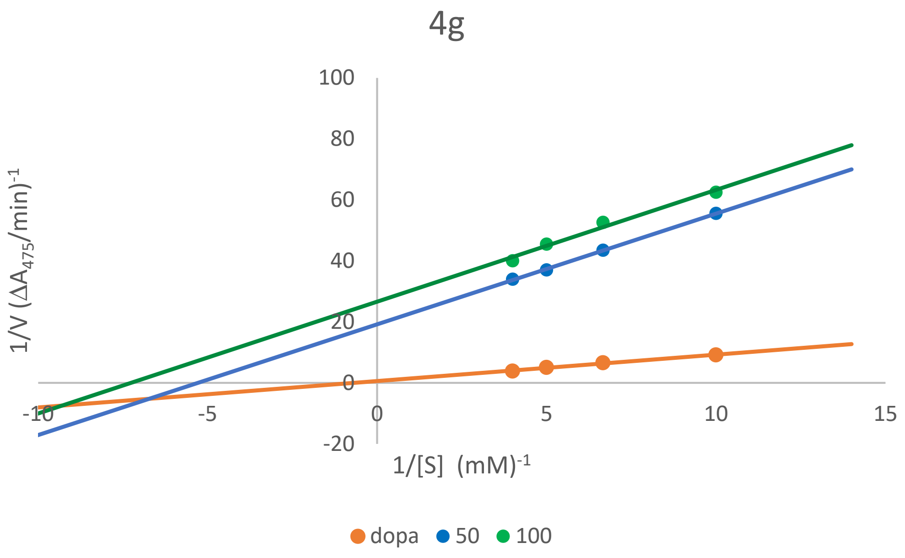
4g
1/V (ΔA475/min)-1
1/[S] (mM)-1
100
80
60
40
20
0
-20
-10
-5
0
5
10
15
dopa
50
100

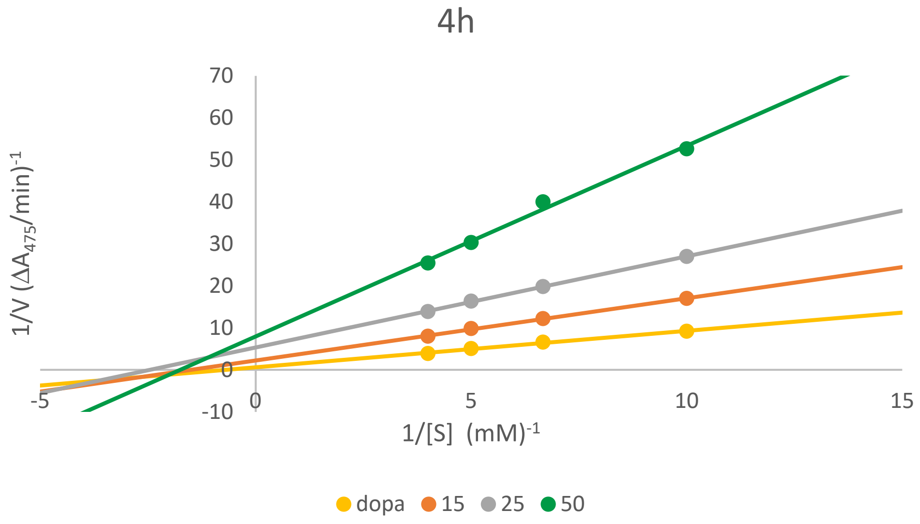
4h
1/V (ΔA475/min)-1
1/[S] (mM)-1
70
60
50
40
30
20
10
0
-10
-5
0
5
10
15
dopa
15
25
50

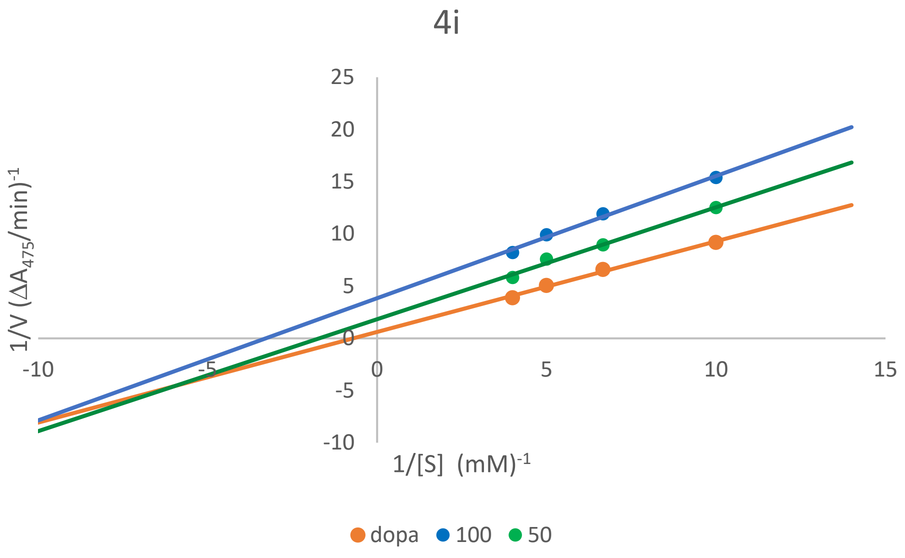
4i
1/V (ΔA475/min)-1
1/[S] (mM)-1
25
20
15
10
5
0
-5
-10
-10
-5
0
5
10
15
dopa
100
50

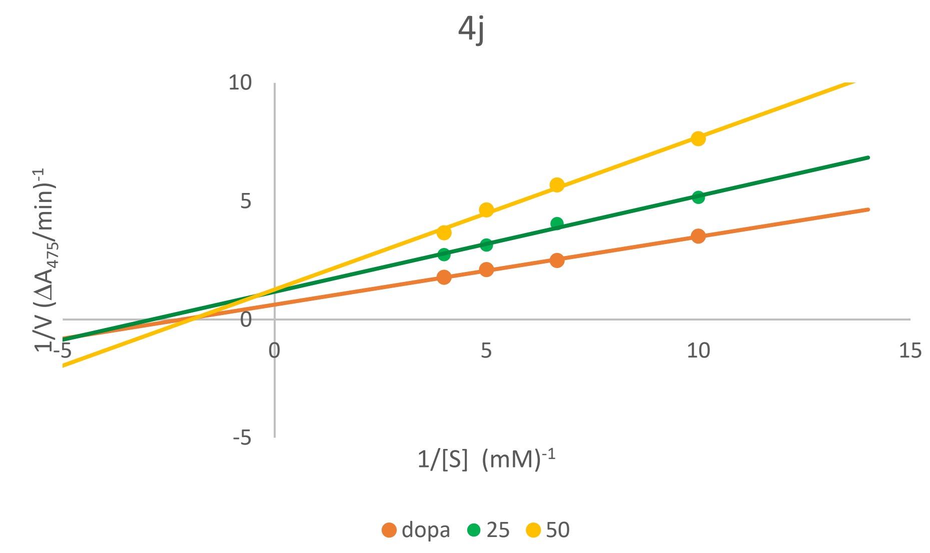
4j
10
5
0
-5
1/V (ΔA475/min)-1
-5
0
5
10
15
1/[S] (mM)-1
dopa
25
50

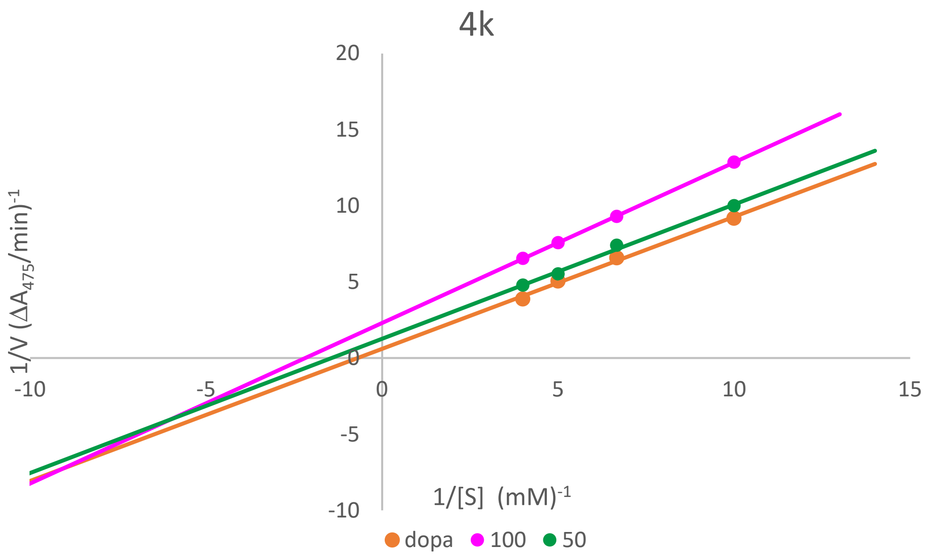
4k
20
15
10
5
0
-5
-10
1/V (ΔA475/min)-1
-10
-5
0
5
10
15
1/[S] (mM)-1
dopa
100
50

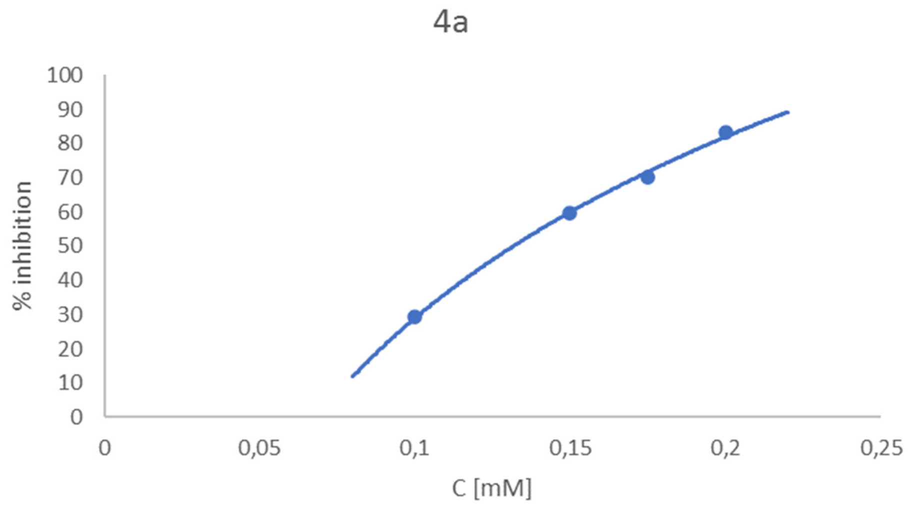
4a
100
90
80
70
60
50
40
30
20
10
0
% inhibition
0
0,05
0,1
0,15
0,2
0,25
C [mM]

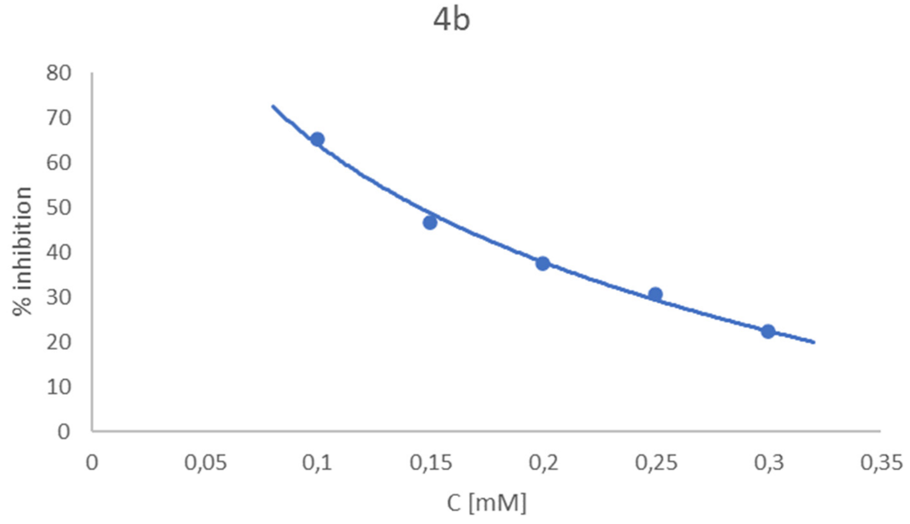
4b
% inhibition
0
10
20
30
40
50
60
70
80
0
0,05
0,1
0,15
0,2
0,25
0,3
0,35
C [mM]

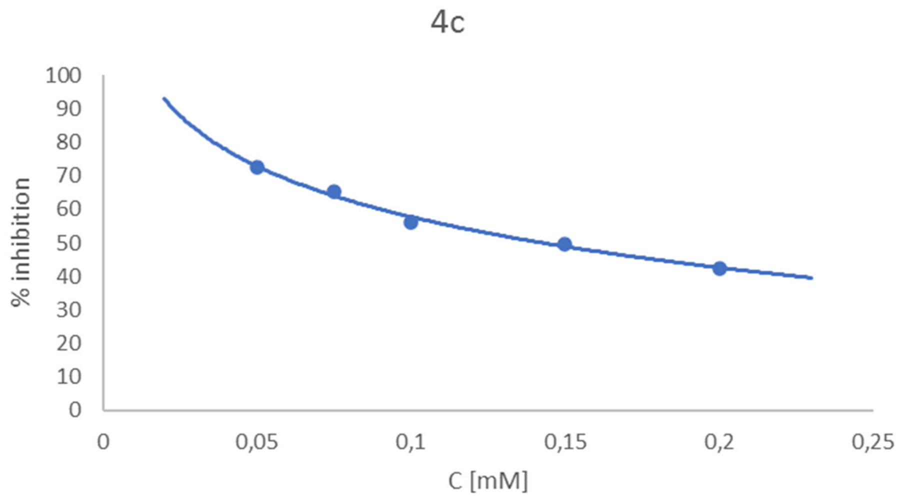
4c
% inhibition
0
10
20
30
40
50
60
70
80
90
100
0
0,05
0,1
0,15
0,2
0,25
C [mM]

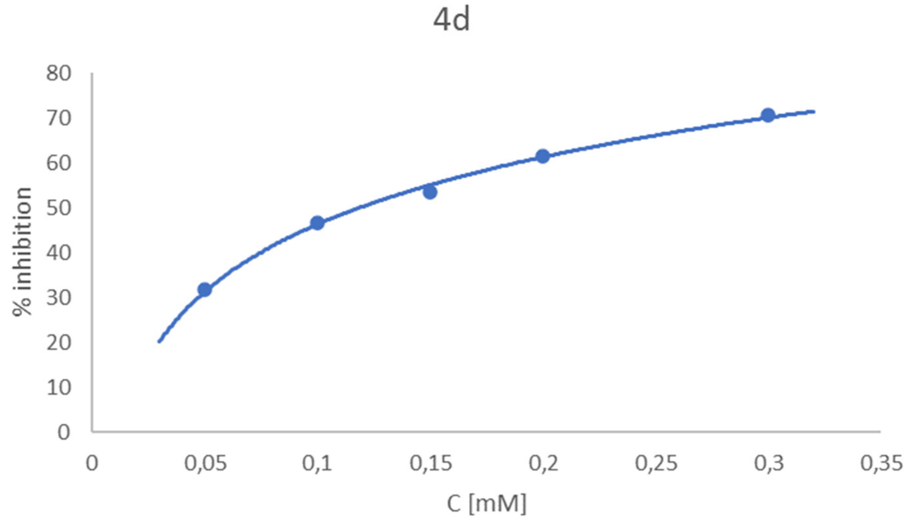
4d
% inhibition
0
10
20
30
40
50
60
70
80
0
0,05
0,1
0,15
0,2
0,25
0,3
0,35
C [mM]

4e

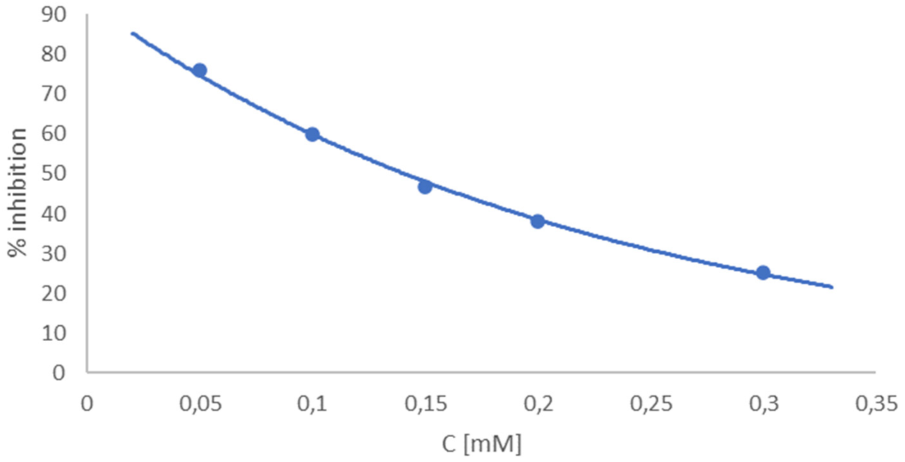

4f

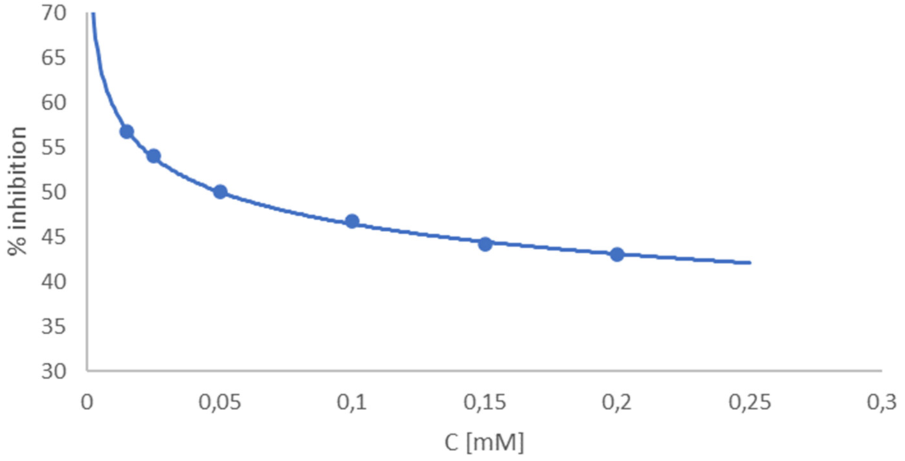

4g

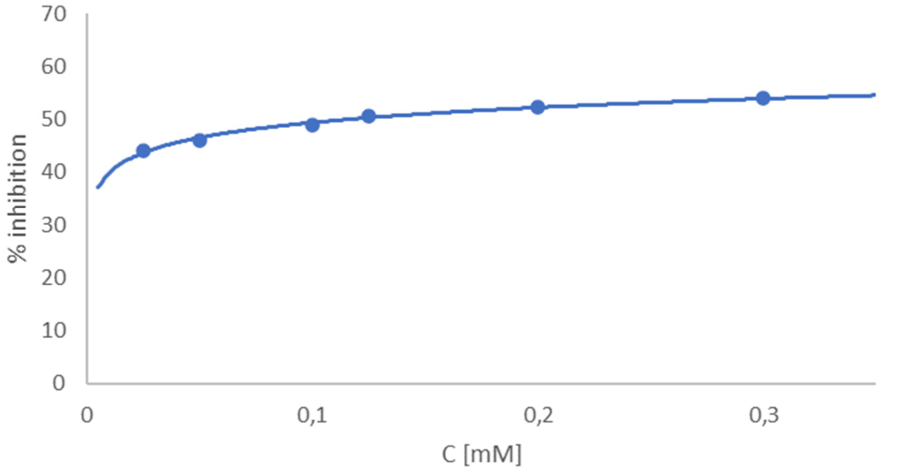

4h

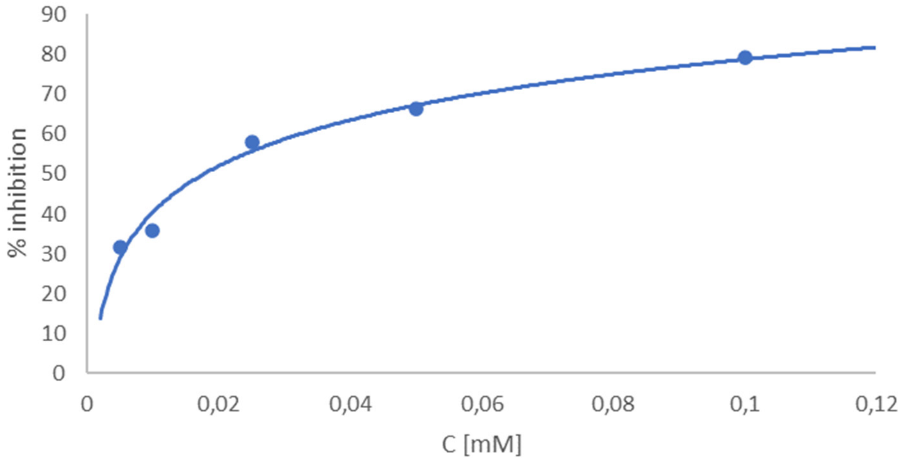
90
80
70
60
50
40
30
20
10
0
% inhibition
0
0,02
0,04
0,06
0,08
0,1
0,12
C [mM]

4i

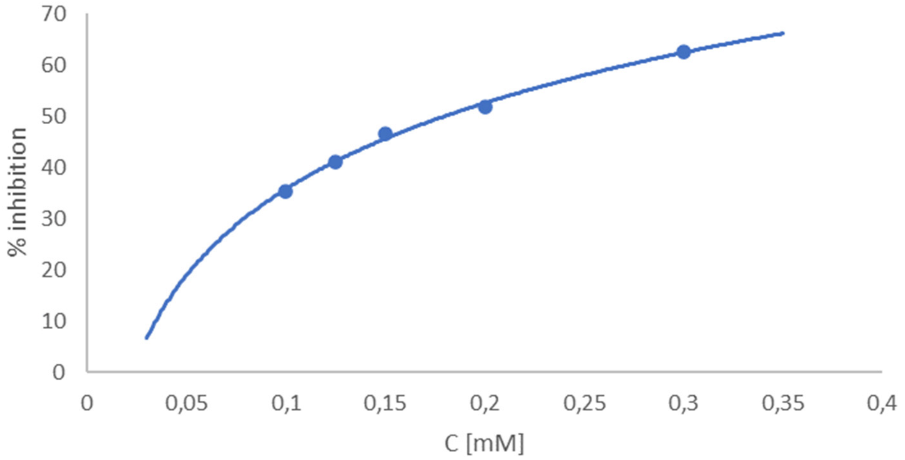
70
60
50
40
30
20
10
0
% inhibition
0
0,05
0,1
0,15
0,2
0,25
0,3
0,35
0,4
C [mM]

4j

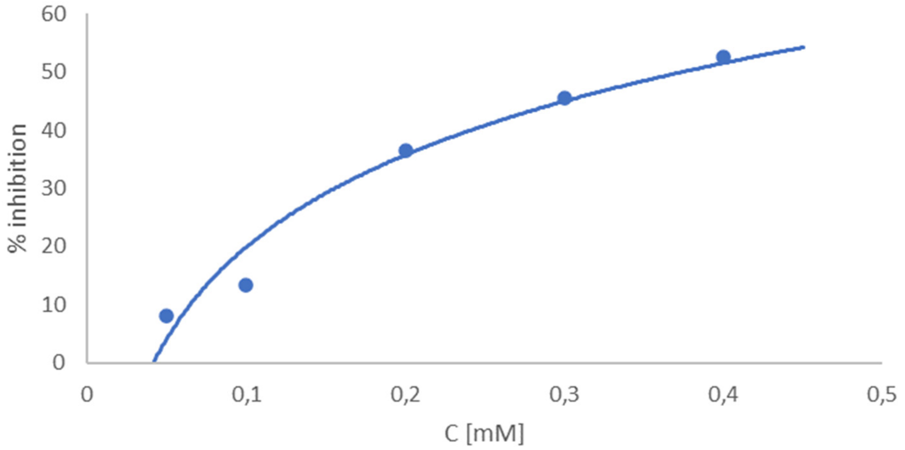
60
50
40
30
20
10
0
% inhibition
0
0,1
0,2
0,3
0,4
0,5
C [mM]

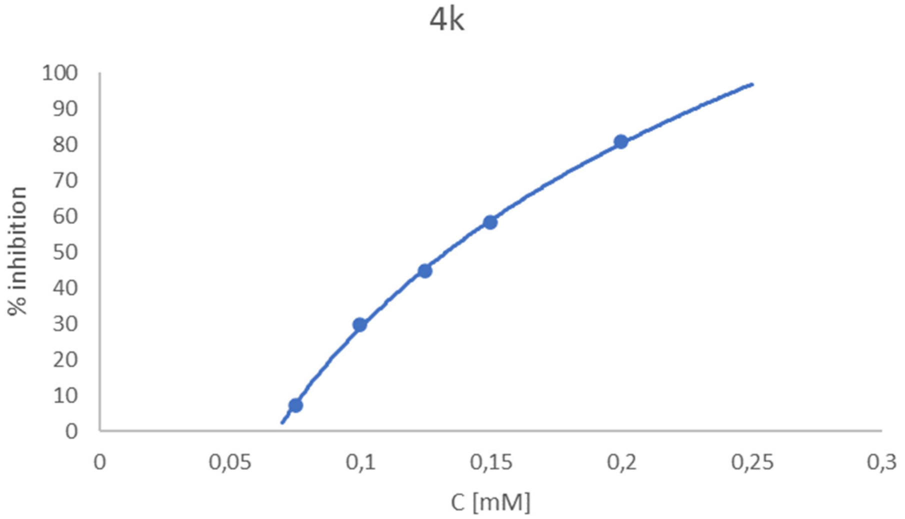
4k
% inhibition
100
90
80
70
60
50
40
30
20
10
0
0
0,05
0,1
0,15
0,2
0,25
0,3
C [mM]

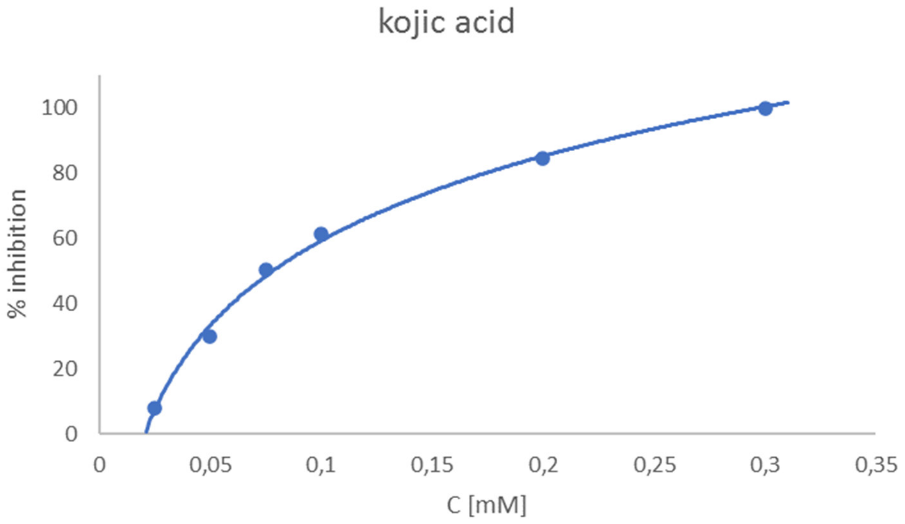
kojic acid
% inhibition
100
80
60
40
20
0
0
0,05
0,1
0,15
0,2
0,25
0,3
0,35
C [mM]

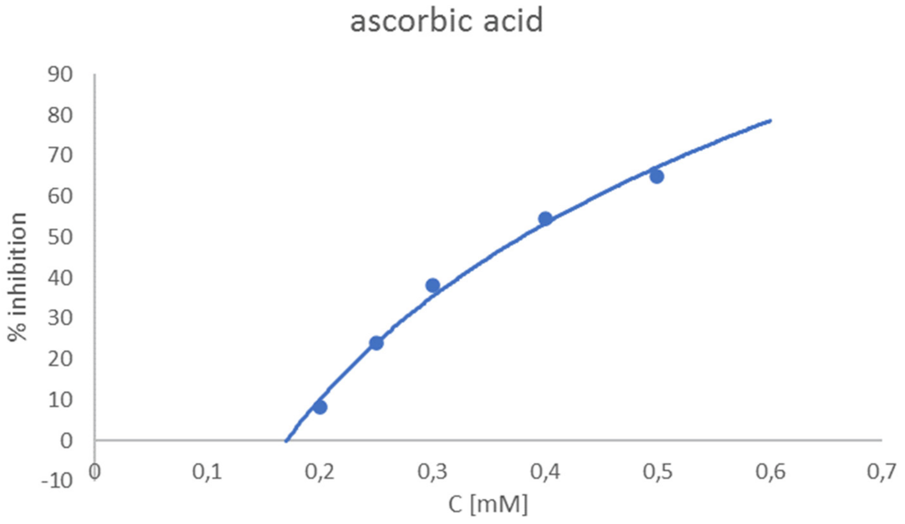
ascorbic acid
% inhibition
90
80
70
60
50
40
30
20
10
0
-10
0
0,1
0,2
0,3
0,4
0,5
0,6
0,7
C [mM]

4f

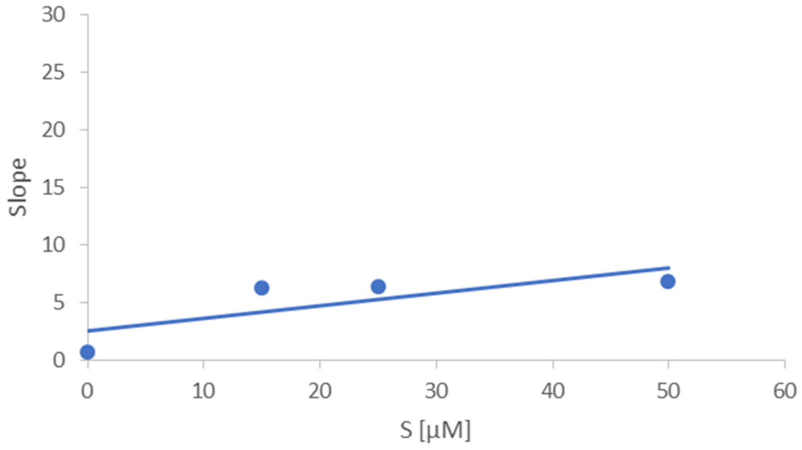
30
25
20
15
10
5
0
Slope
0
10
20
30
40
50
60
S [µM]

4h

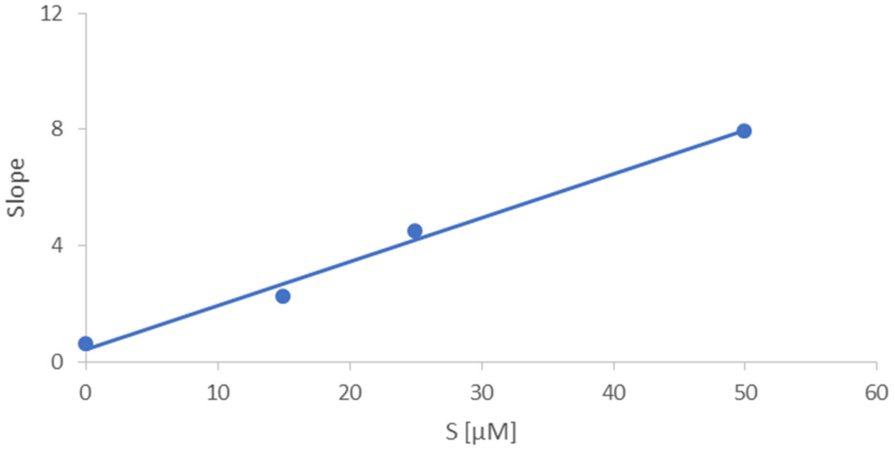
12
8
4
0
Slope
0
10
20
30
40
50
60
S [µM]
